# Supplementary material for: The transaminase-ω-amidase pathway senses oxidative stress to control glutamine metabolism and α-ketoglutarate levels in endothelial cells
Source: EMBO J. 2025 Dec 17;45(3):820–55. doi: 10.1038/s44318-025-00642-7 (PMC12864753; doi:10.1038/s44318-025-00642-7)
Supplement: Supplementary file 9 — Source data Fig. 2 [file 44318_2025_642_MOESM9_ESM.zip › Figure 2/Fig. 2C.pptx]

## Slide 1
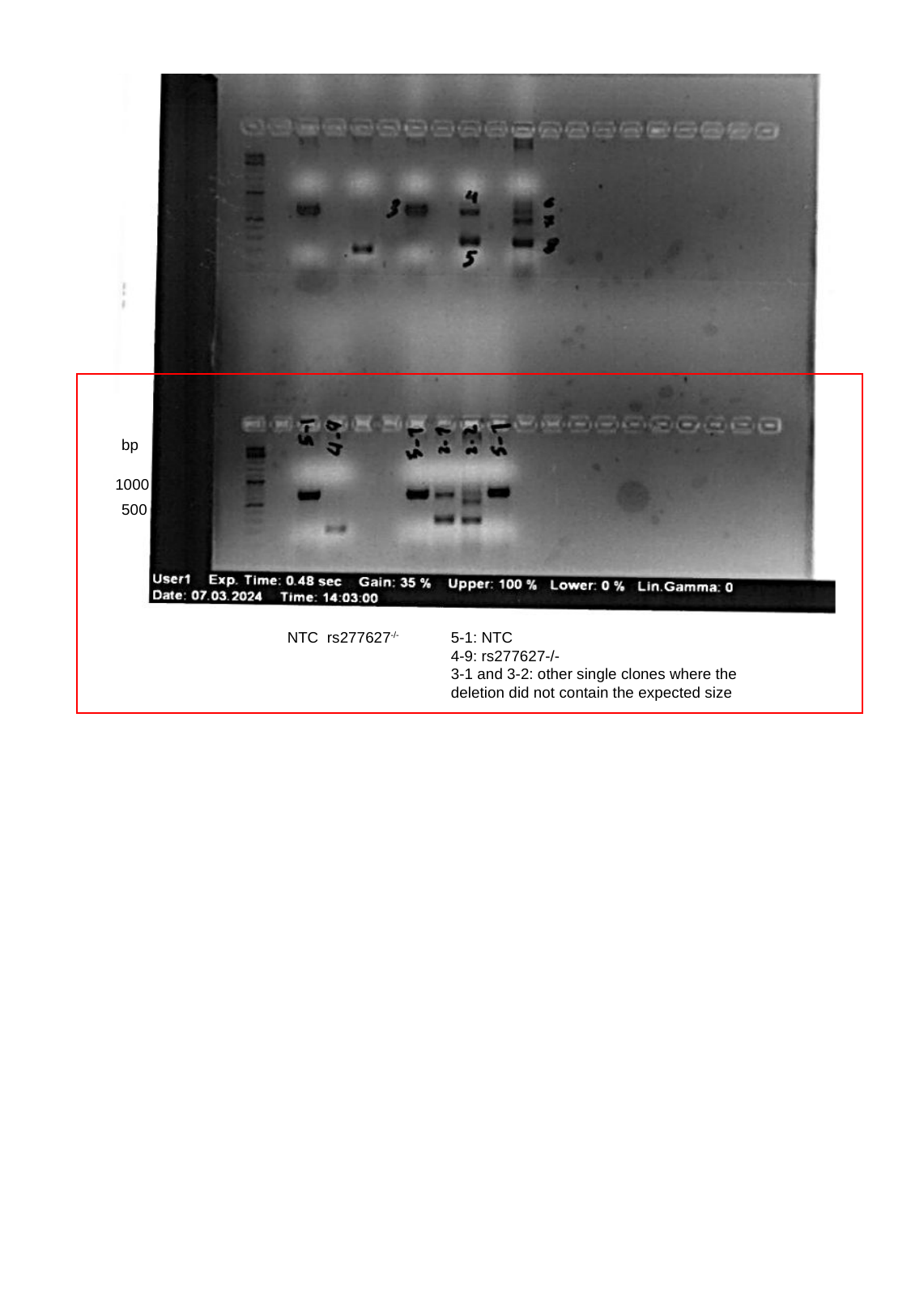

bp
1000
500
NTC
rs277627-/-
5-1: NTC
4-9: rs277627-/-
3-1 and 3-2: other single clones where the deletion did not contain the expected size
