# Supplementary figures and images for: The transaminase-ω-amidase pathway senses oxidative stress to control glutamine metabolism and α-ketoglutarate levels in endothelial cells
Source: EMBO J. 2025 Dec 17;45(3):820–55. doi: 10.1038/s44318-025-00642-7 (PMC12864753; doi:10.1038/s44318-025-00642-7)

## Slide 1
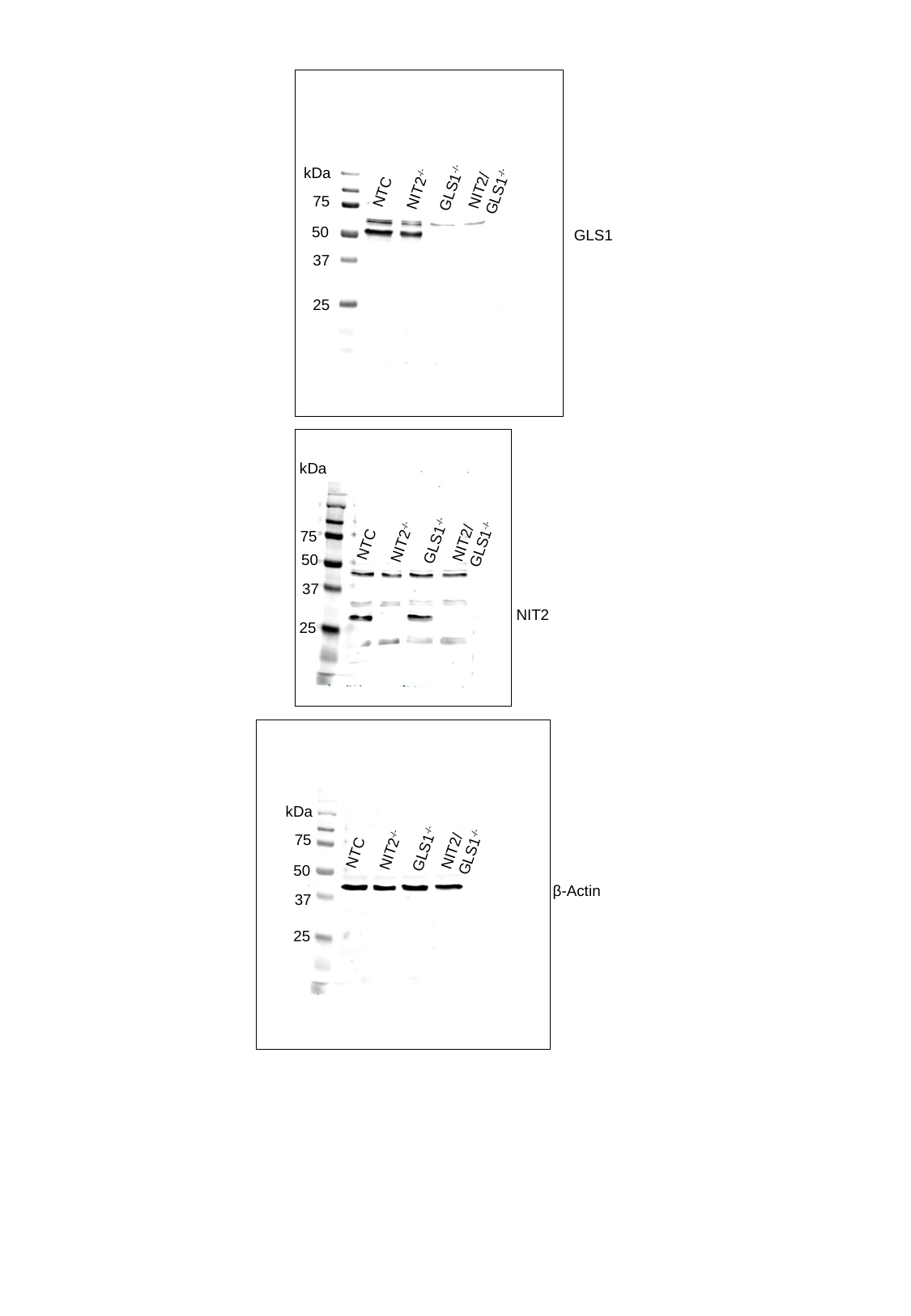

NIT2/
GLS1-/-
kDa
GLS1-/-
NIT2-/-
NTC
75
50
GLS1
37
25
kDa
NIT2/
GLS1-/-
75
GLS1-/-
NIT2-/-
NTC
50
37
NIT2
25
NIT2/
GLS1-/-
kDa
75
GLS1-/-
NIT2-/-
NTC
50
β-Actin
37
25

Supplement: Supplementary file 10 — Source data Fig. 3 [file 44318_2025_642_MOESM10_ESM.zip › Figure 3/Fig. 3A.pptx]

## Slide 1
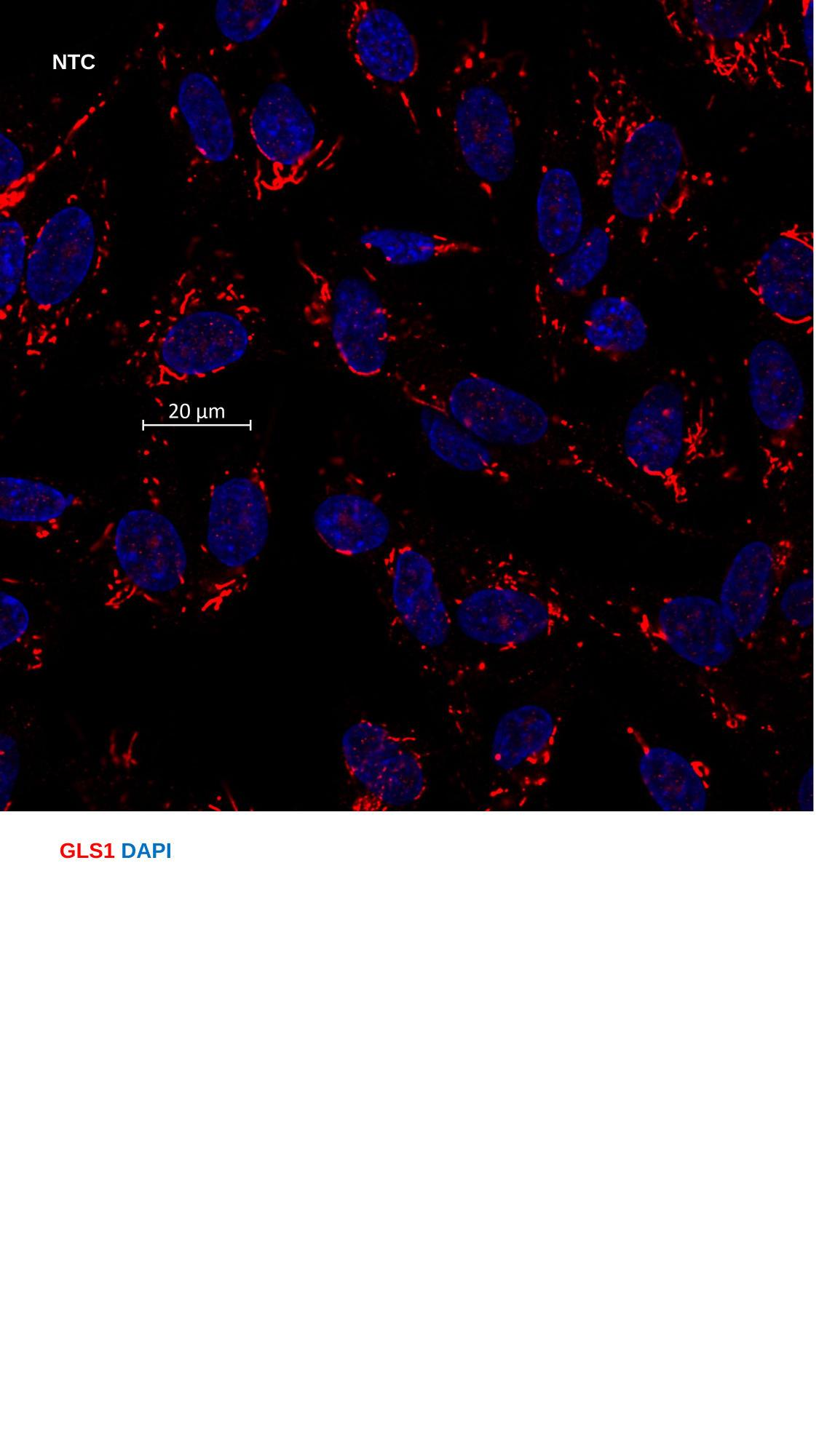

NTC
GLS1 DAPI

## Slide 2
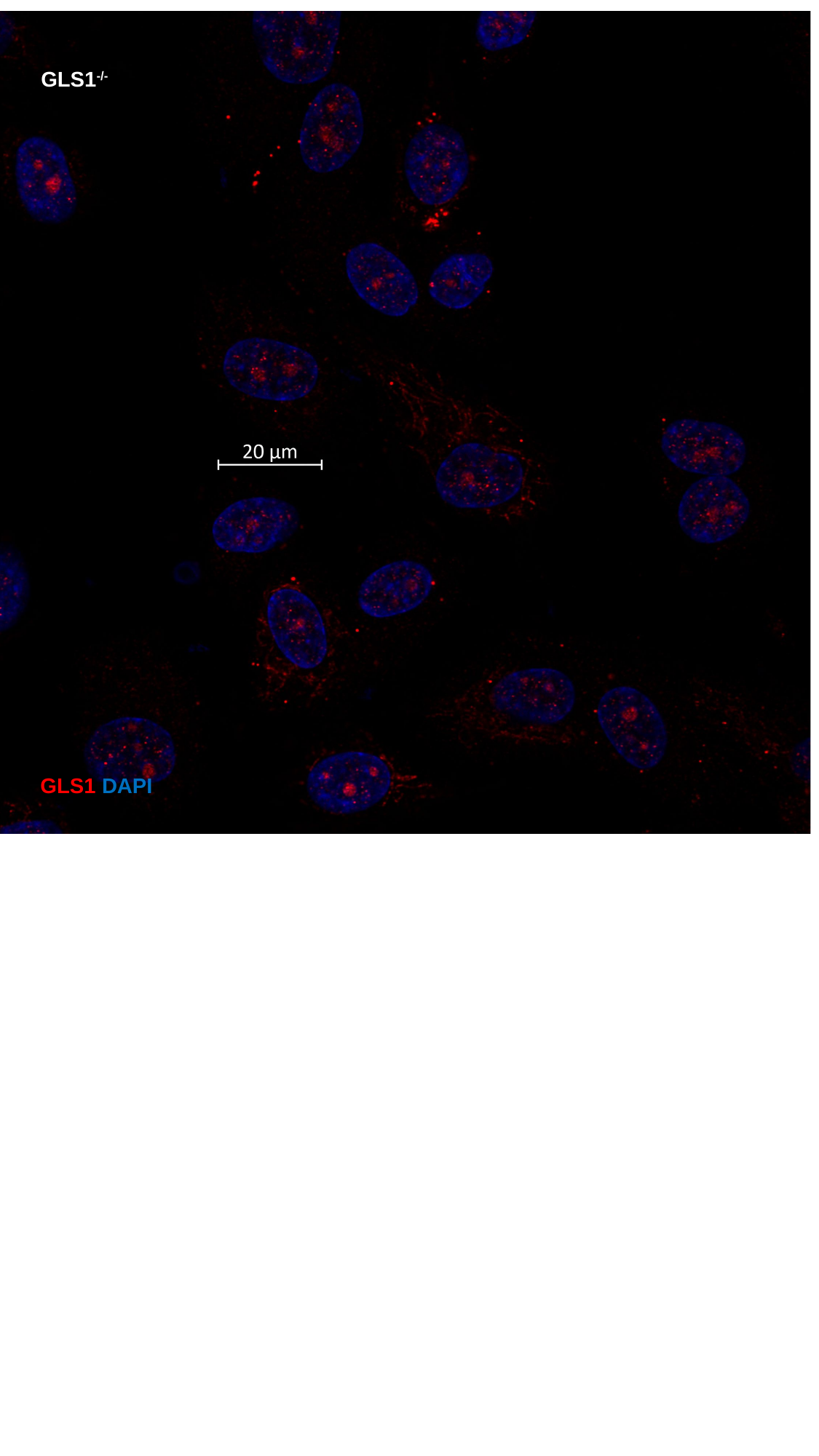

GLS1-/-
GLS1 DAPI

## Slide 3
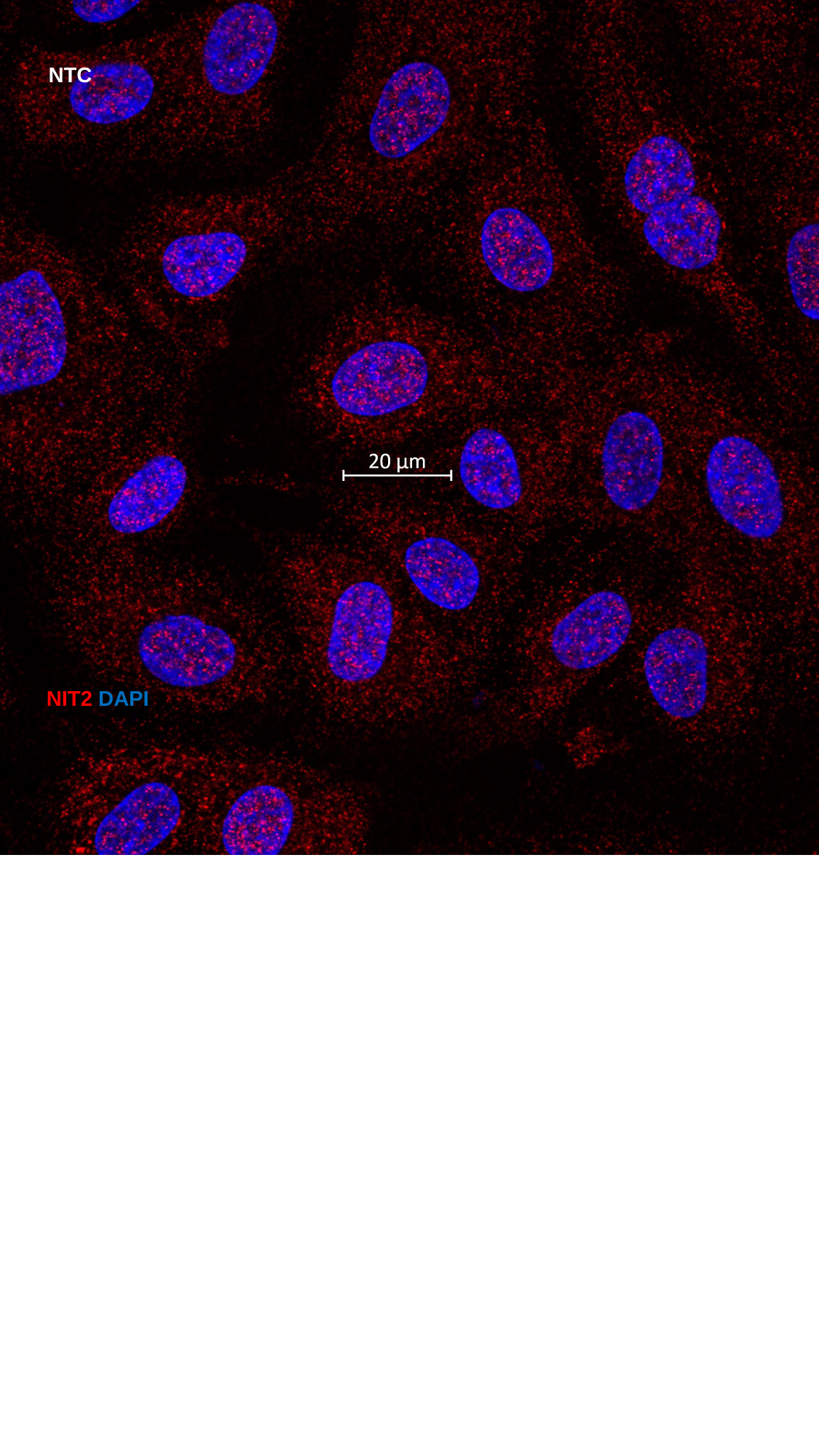

NTC
NIT2 DAPI

## Slide 4
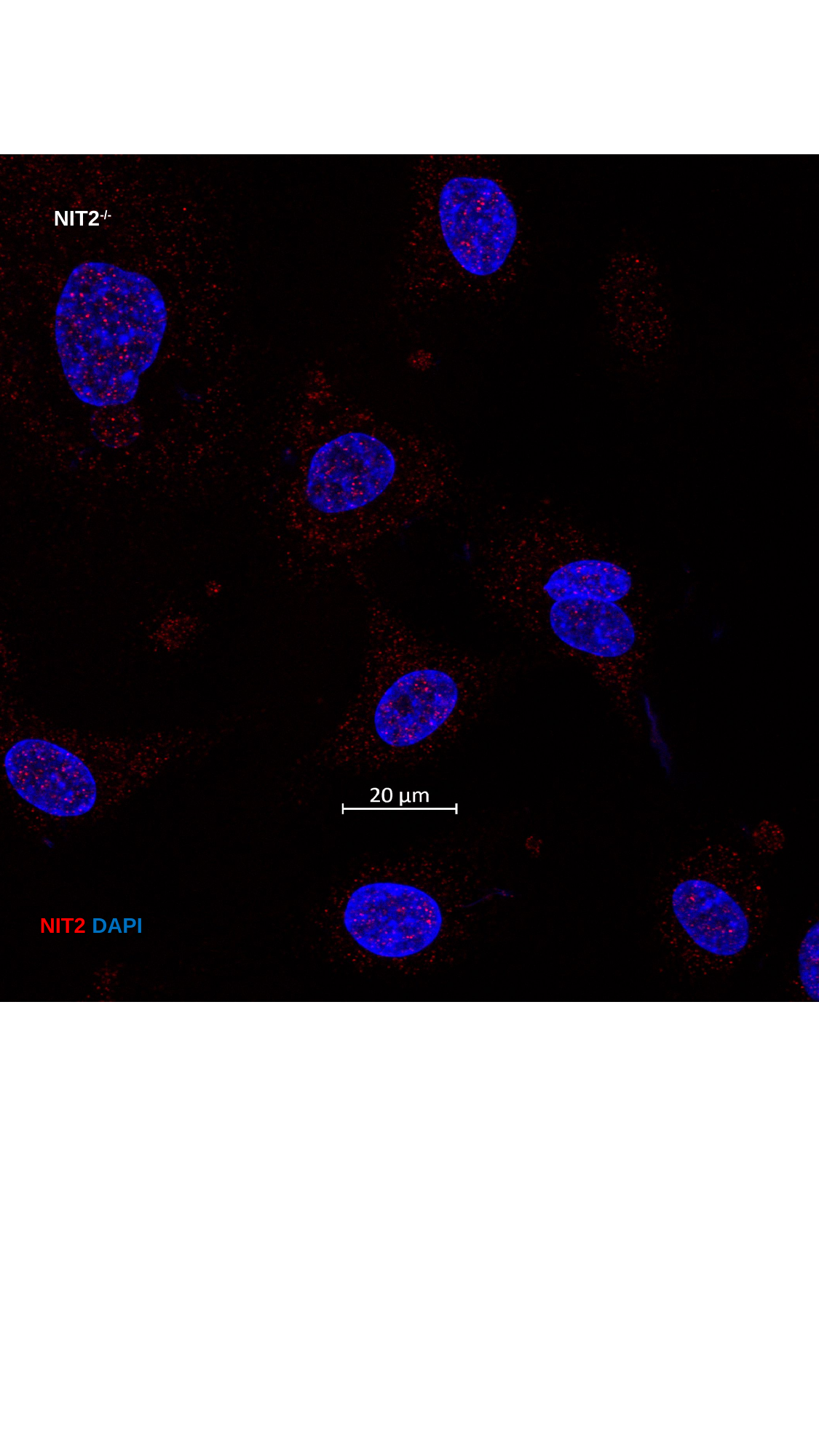

NIT2-/-
NIT2 DAPI

Supplement: Supplementary file 10 — Source data Fig. 3 [file 44318_2025_642_MOESM10_ESM.zip › Figure 3/Fig. 3B.pptx]

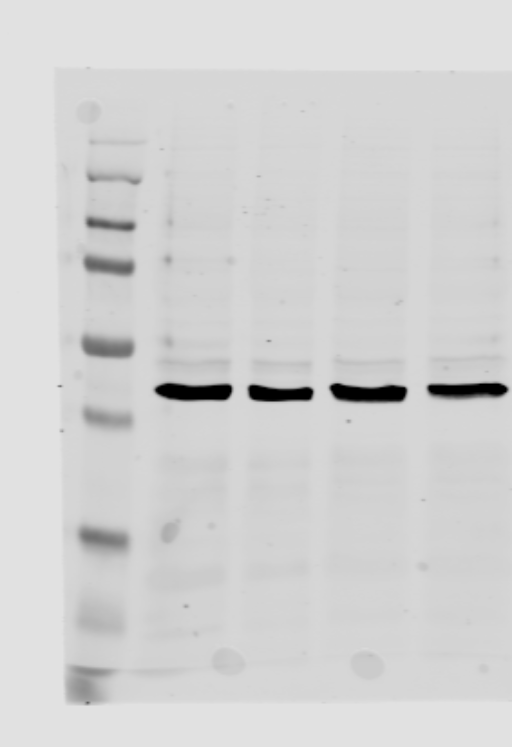

Supplement: Supplementary file 10 — Source data Fig. 3 [file 44318_2025_642_MOESM10_ESM.zip › Figure 3/TIFF Files WB/NM206 actin.tif]

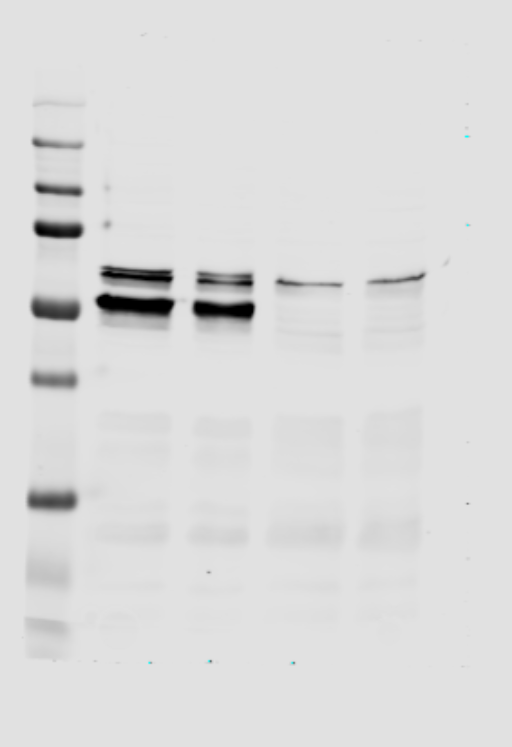

Supplement: Supplementary file 10 — Source data Fig. 3 [file 44318_2025_642_MOESM10_ESM.zip › Figure 3/TIFF Files WB/NM206 GLS1.tif]

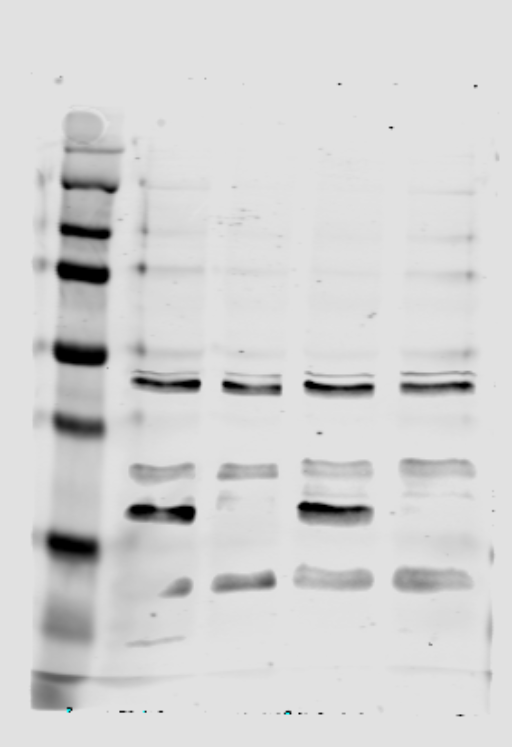

Supplement: Supplementary file 10 — Source data Fig. 3 [file 44318_2025_642_MOESM10_ESM.zip › Figure 3/TIFF Files WB/NM206 NIT2.tif]

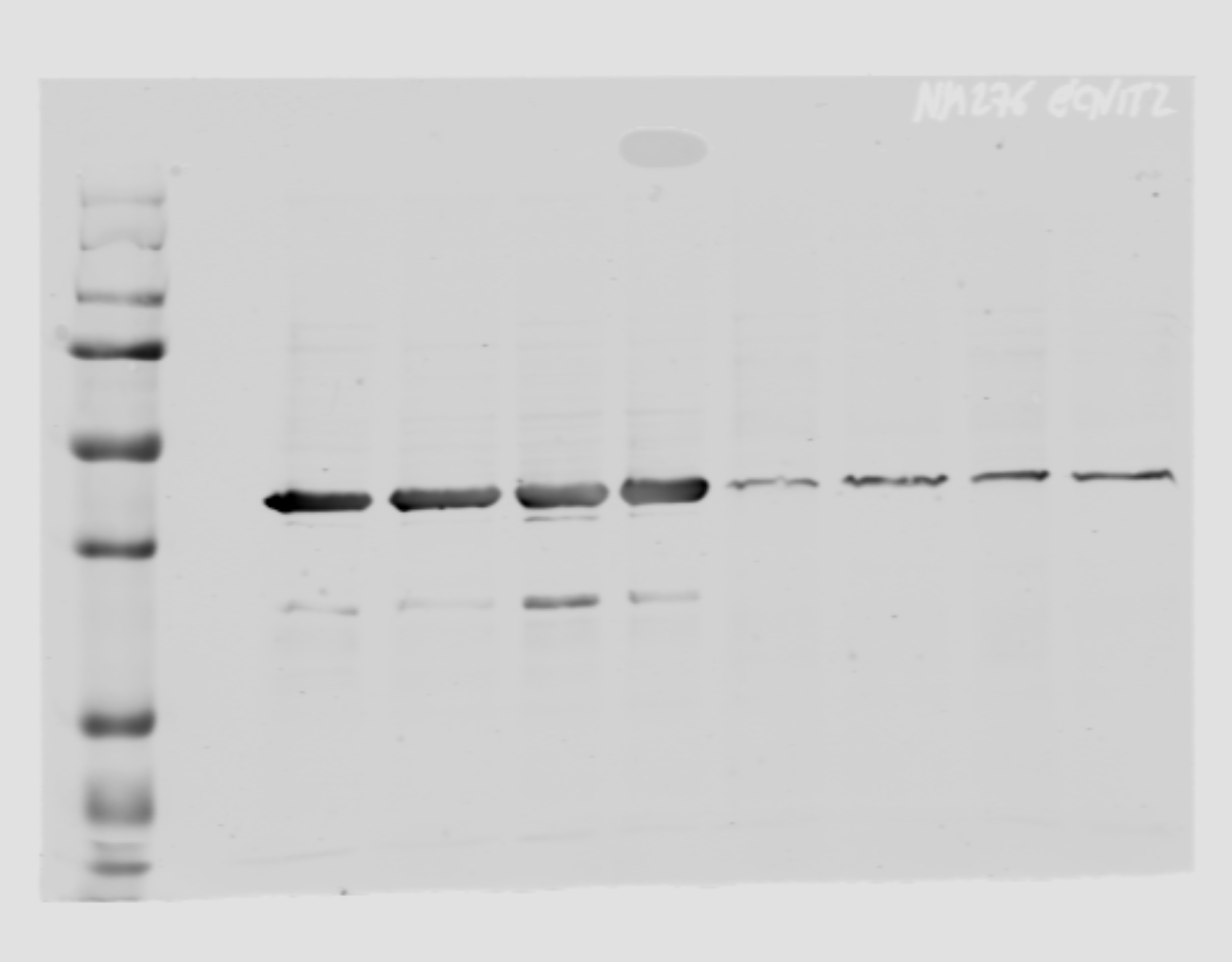

Supplement: Supplementary file 11 — Source data Fig. 4 [file 44318_2025_642_MOESM11_ESM.zip › Figure 4/TIFF Files WB images/NM276_ecNIT2 KO mouse_aSMCactin antibody 700.tif]

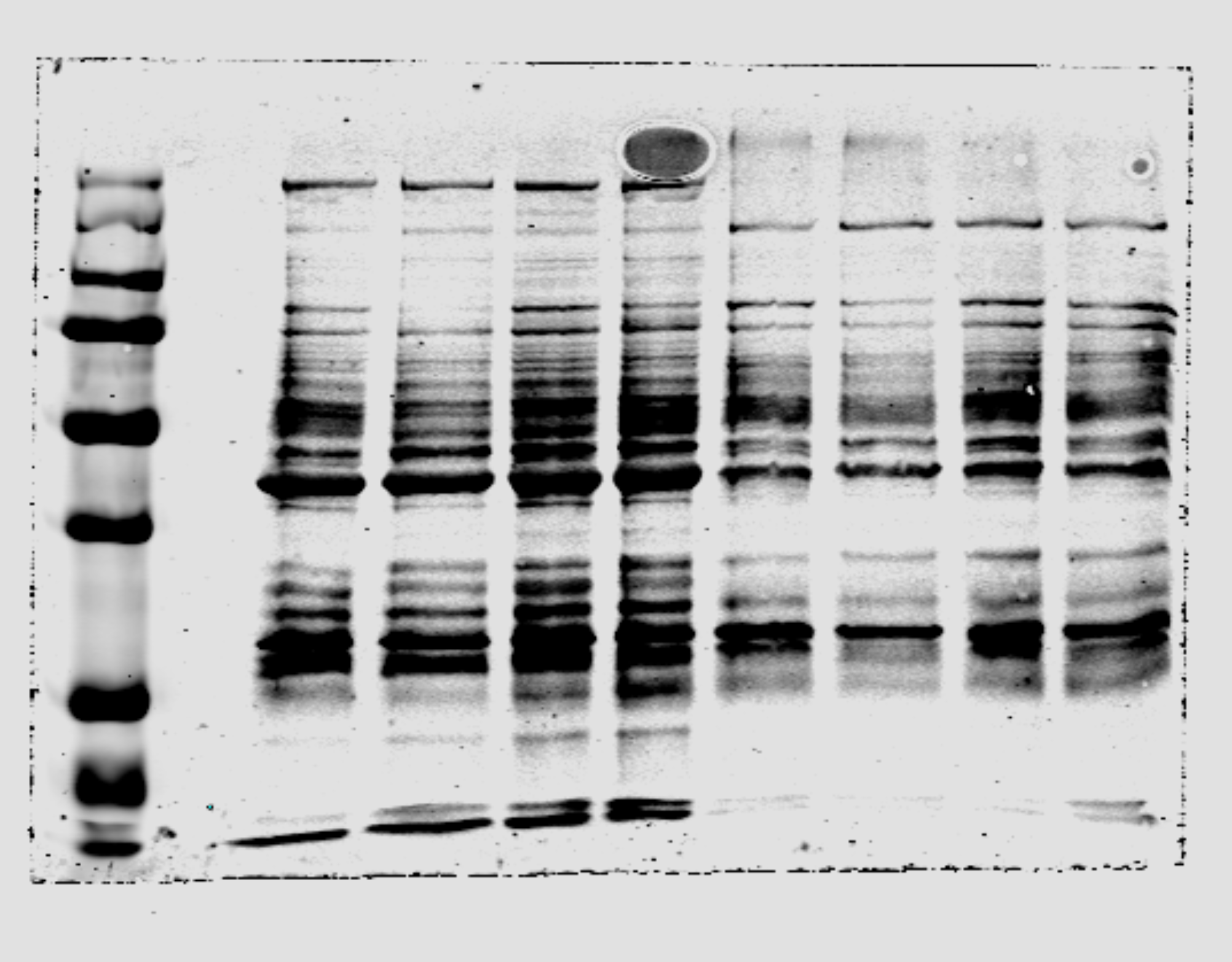

Supplement: Supplementary file 11 — Source data Fig. 4 [file 44318_2025_642_MOESM11_ESM.zip › Figure 4/TIFF Files WB images/NM276_ecNIT2 KO mouse_eNOS antibody.tif]

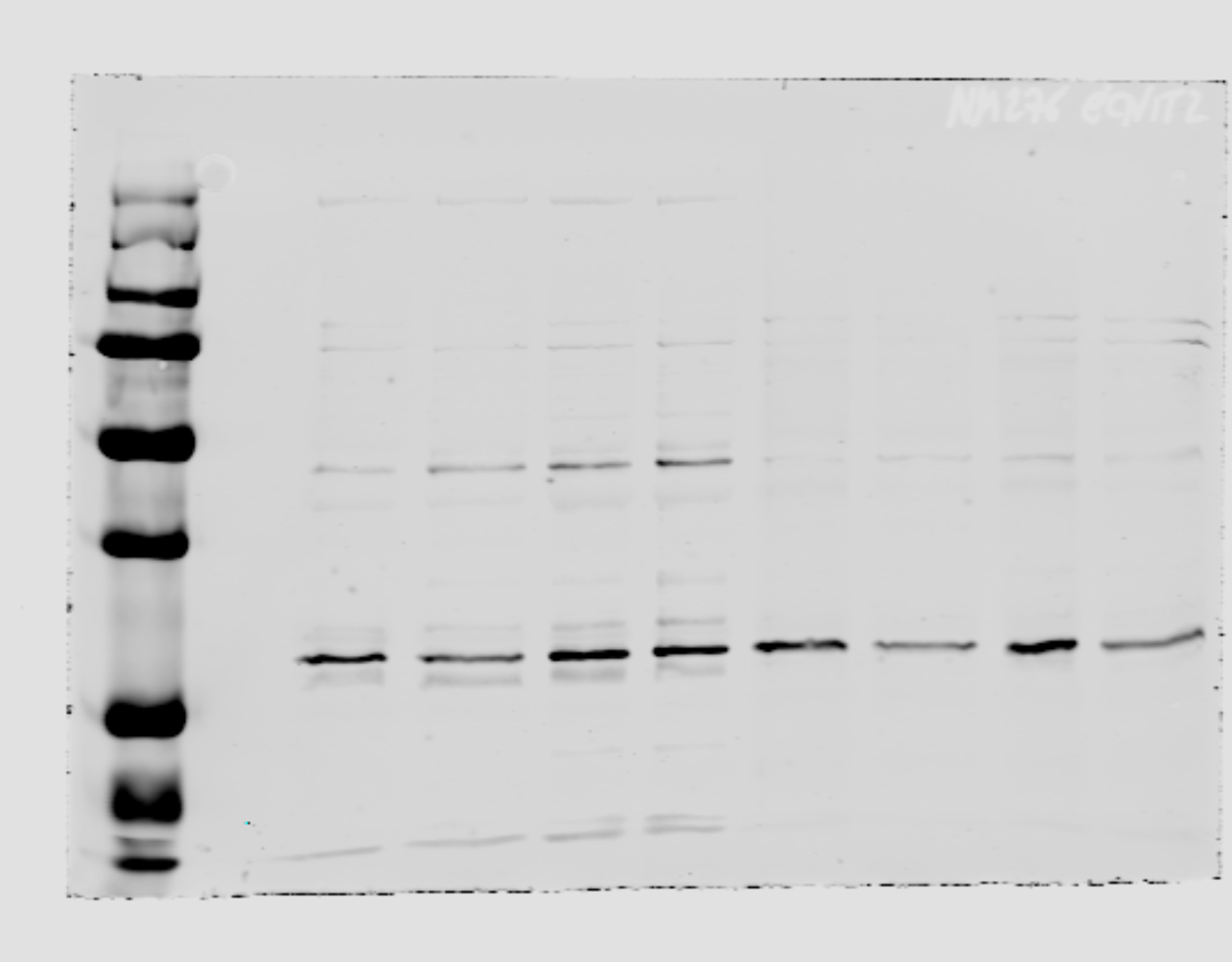

Supplement: Supplementary file 11 — Source data Fig. 4 [file 44318_2025_642_MOESM11_ESM.zip › Figure 4/TIFF Files WB images/NM276_ecNIT2 KO mouse_NIT2 antibody.tif]

## Slide 1
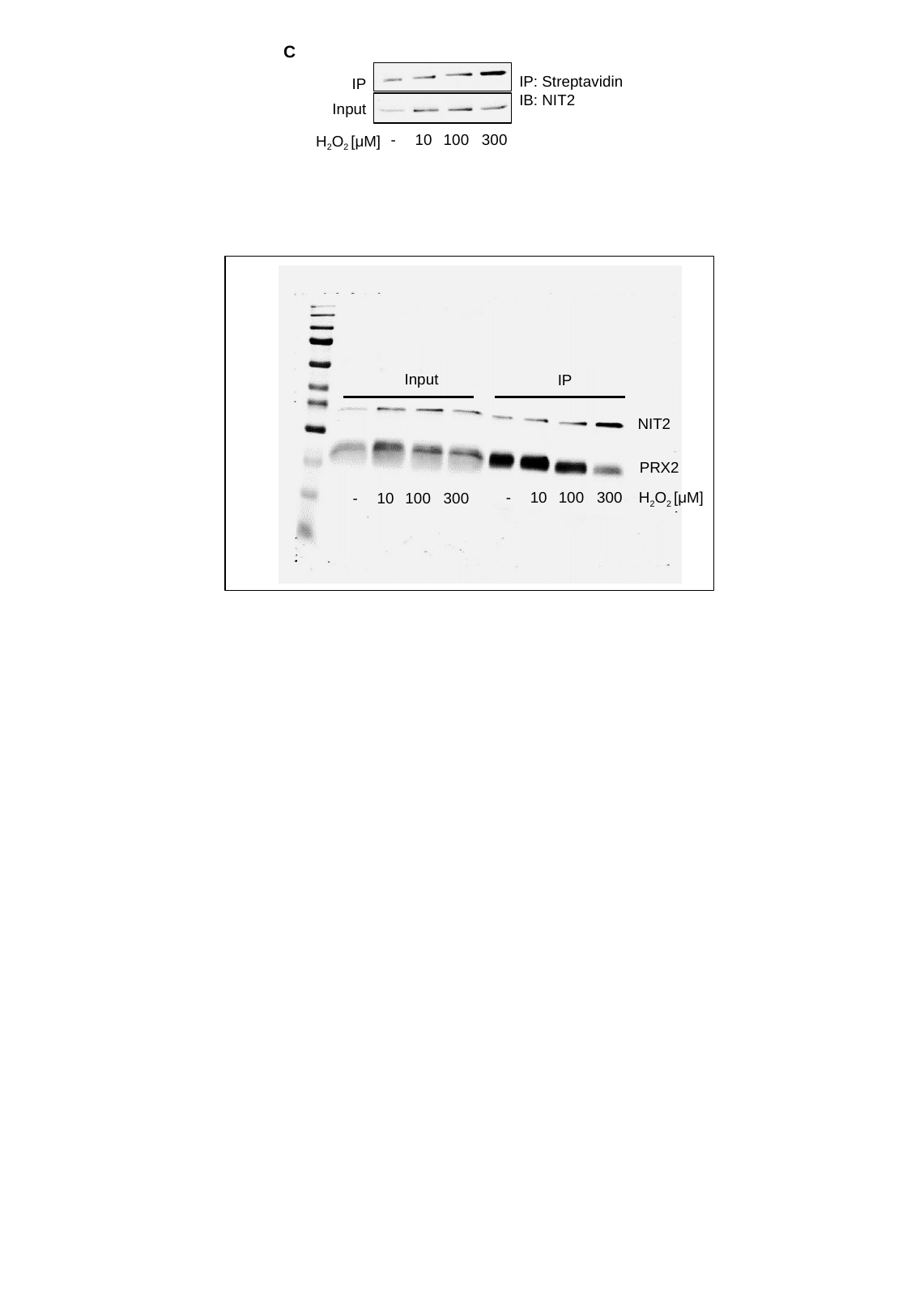

C
IP: Streptavidin
IB: NIT2
IP
Input
-
10
100
300
H2O2 [μM]
Input
IP
NIT2
PRX2
-
10
100
300
H2O2 [μM]
-
10
100
300

Supplement: Supplementary file 13 — Source data Fig. 6 [file 44318_2025_642_MOESM13_ESM.zip › Figure 6/Fig. 6C.pptx]

## Slide 1
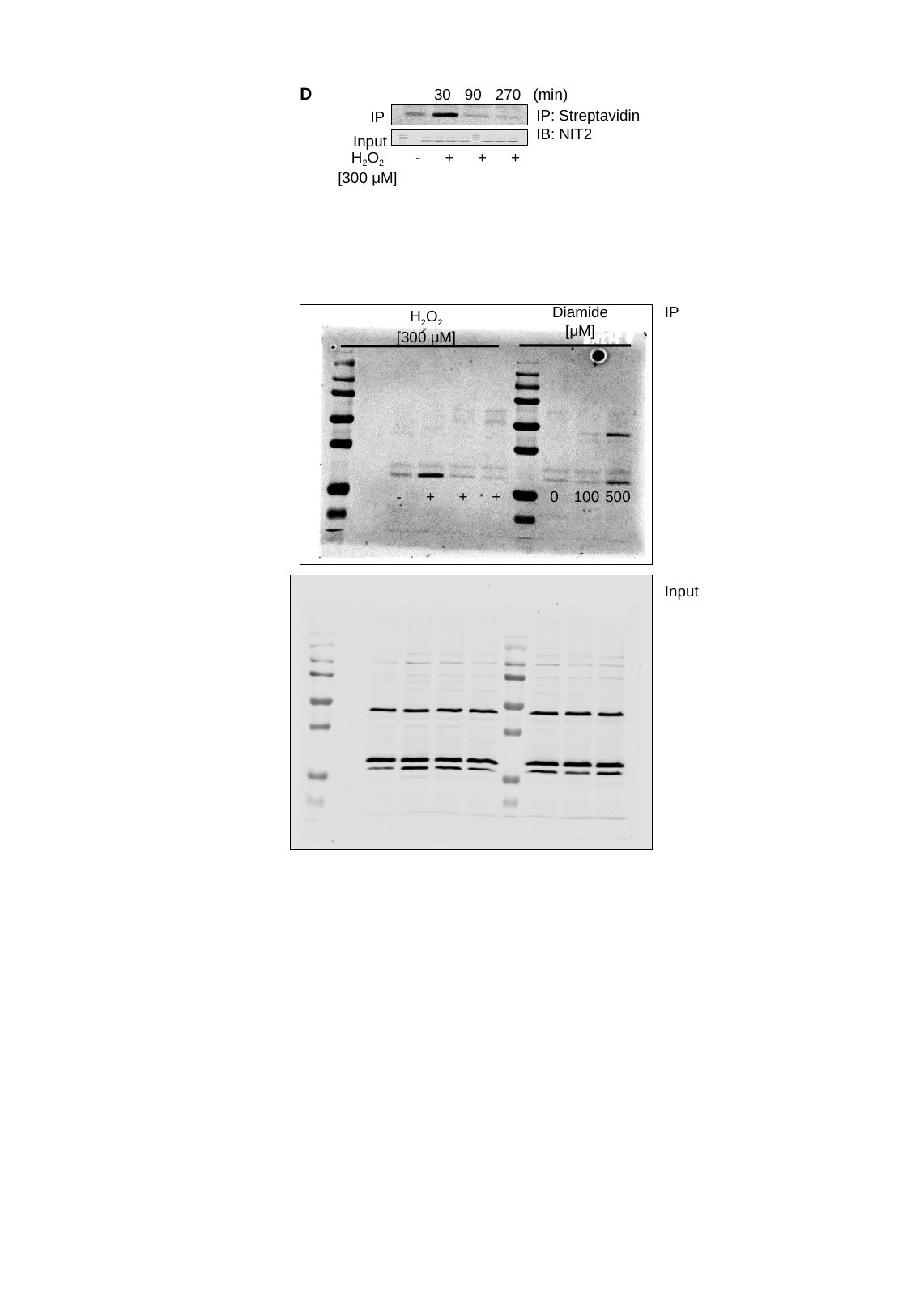

D
30
90
270
(min)
IP: Streptavidin
IB: NIT2
IP
Input
H2O2
[300 μM]
-
+
+
+
IP
Diamide
[μM]
H2O2
[300 μM]
-
+
+
+
0
100
500
Input

Supplement: Supplementary file 13 — Source data Fig. 6 [file 44318_2025_642_MOESM13_ESM.zip › Figure 6/Fig. 6D.pptx]

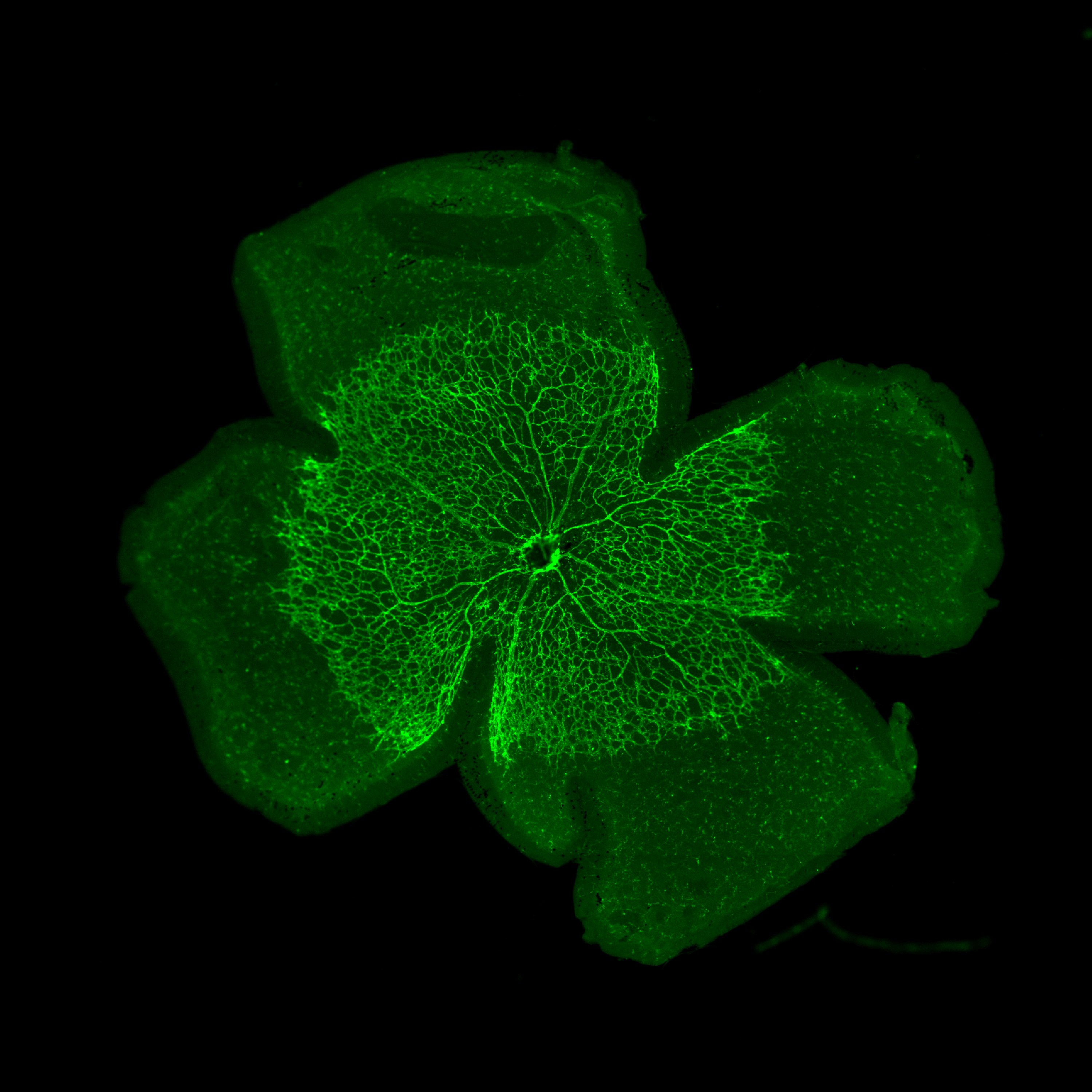

Supplement: Supplementary file 15 — Figure EV4 Source Data [file 44318_2025_642_MOESM15_ESM.zip › EV_4/EV 4F/GlobalNIT2_1_KO.tif]

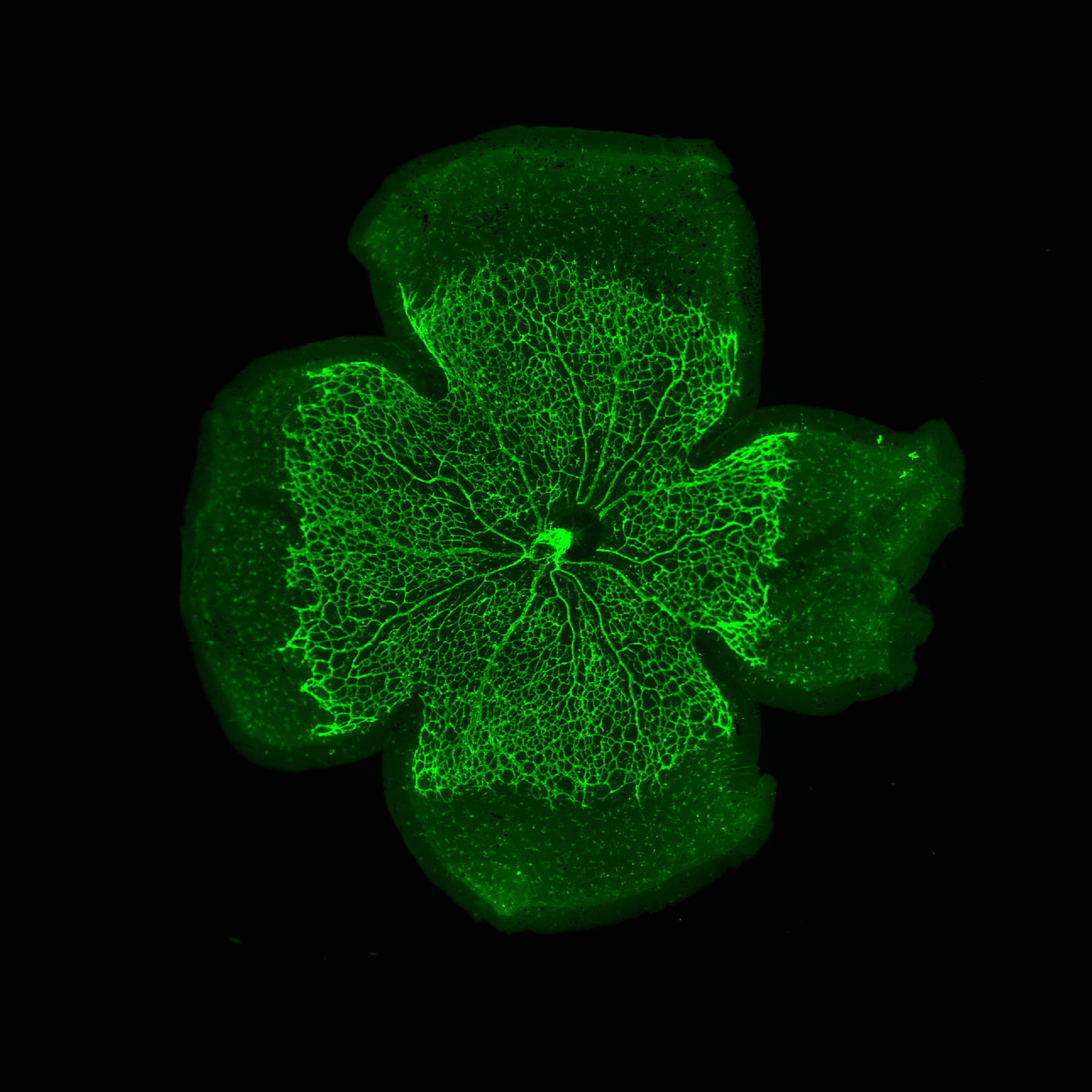

Supplement: Supplementary file 15 — Figure EV4 Source Data [file 44318_2025_642_MOESM15_ESM.zip › EV_4/EV 4F/GlobalNIT2_1_WT.tif]

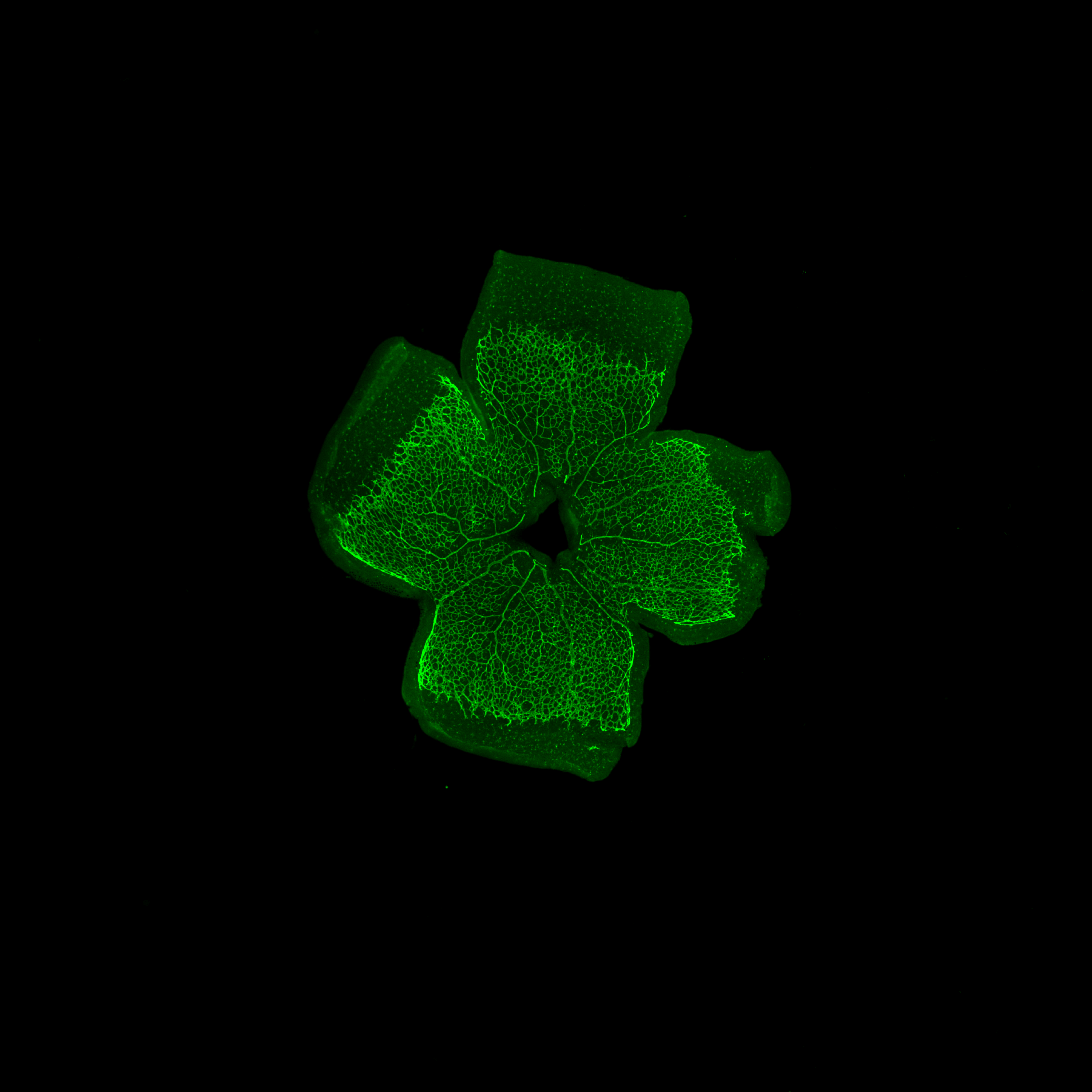

Supplement: Supplementary file 15 — Figure EV4 Source Data [file 44318_2025_642_MOESM15_ESM.zip › EV_4/EV 4F/GlobalNIT2_2_KO.tif]

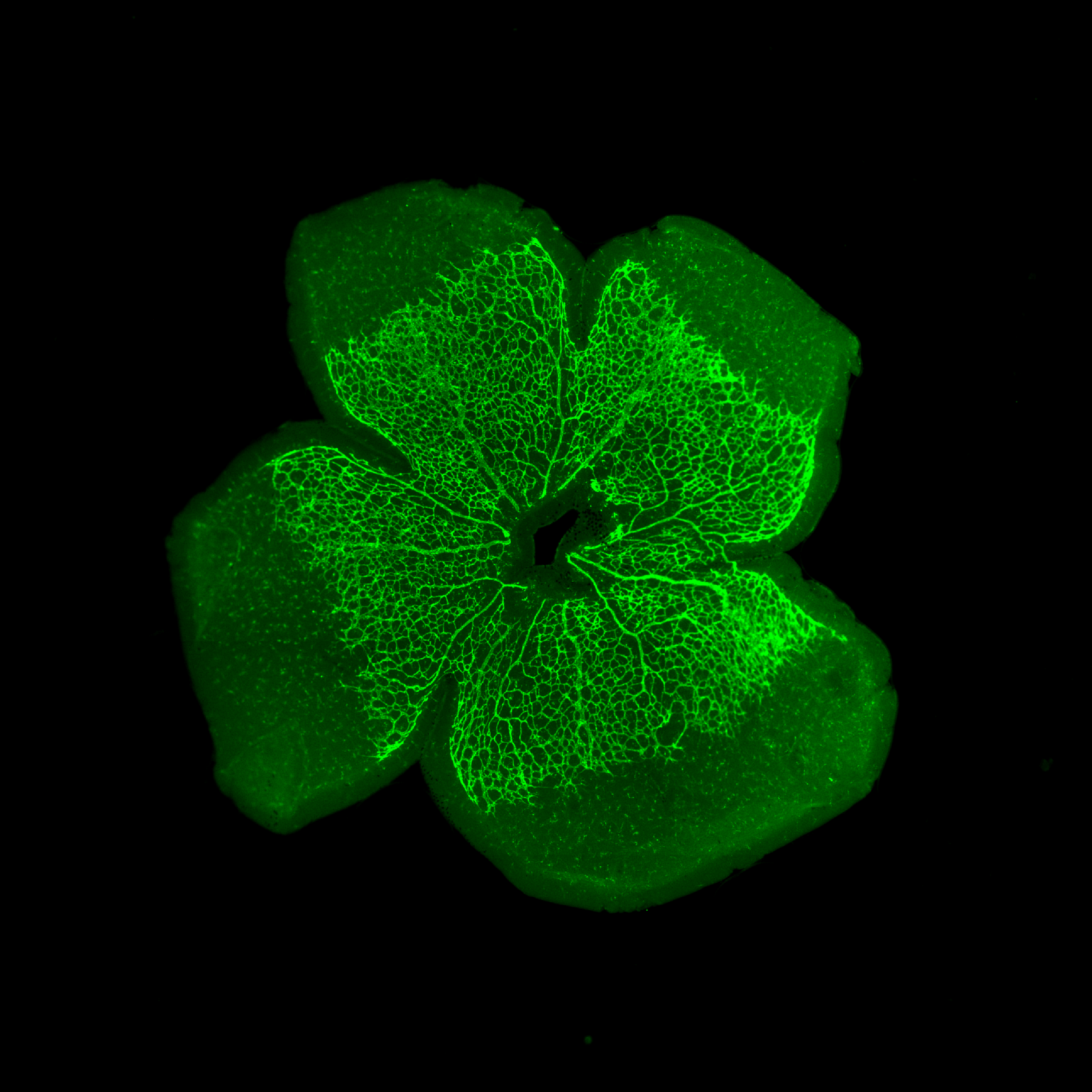

Supplement: Supplementary file 15 — Figure EV4 Source Data [file 44318_2025_642_MOESM15_ESM.zip › EV_4/EV 4F/GlobalNIT2_2_WT.tif]

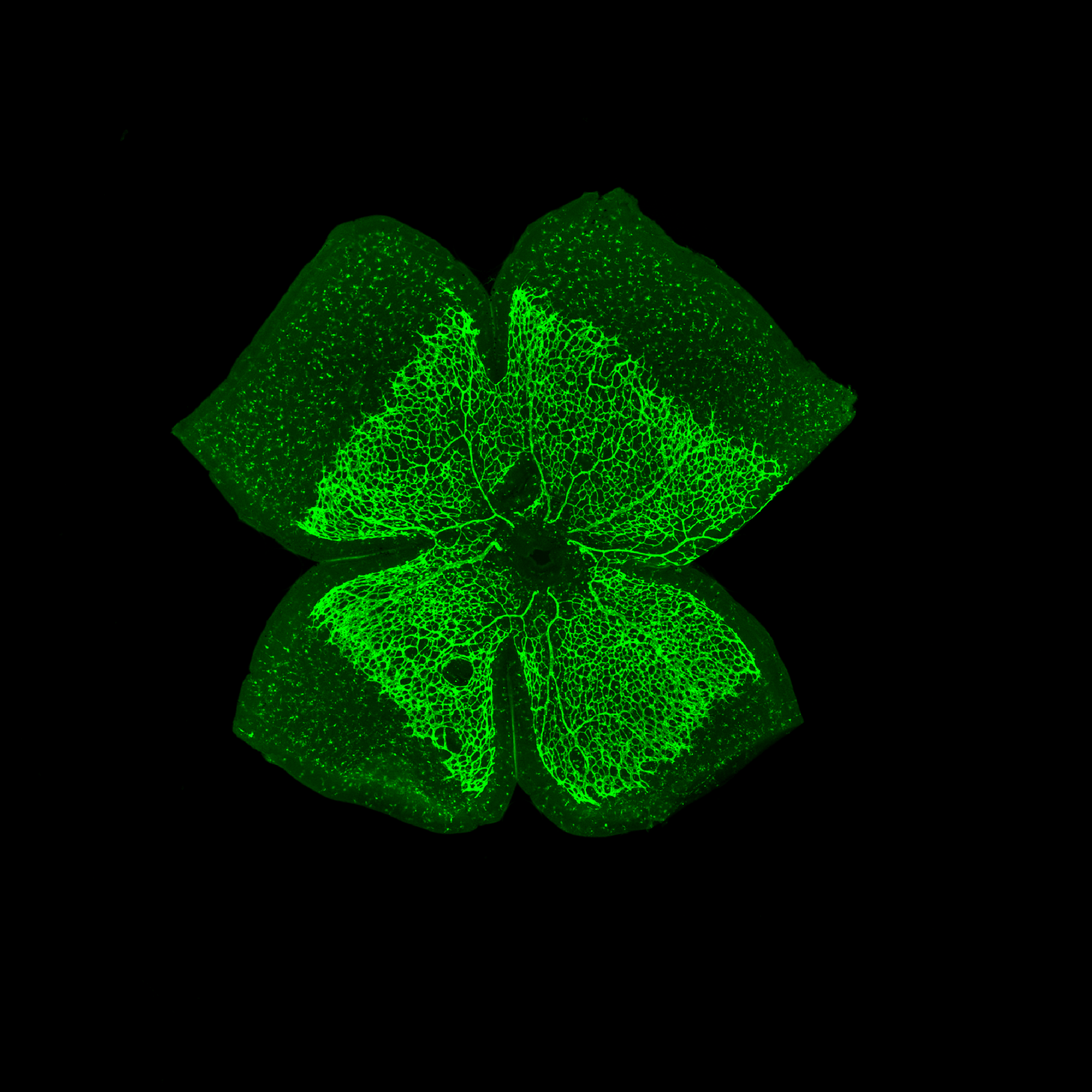

Supplement: Supplementary file 15 — Figure EV4 Source Data [file 44318_2025_642_MOESM15_ESM.zip › EV_4/EV 4F/GlobalNIT2_3_KO.tif]

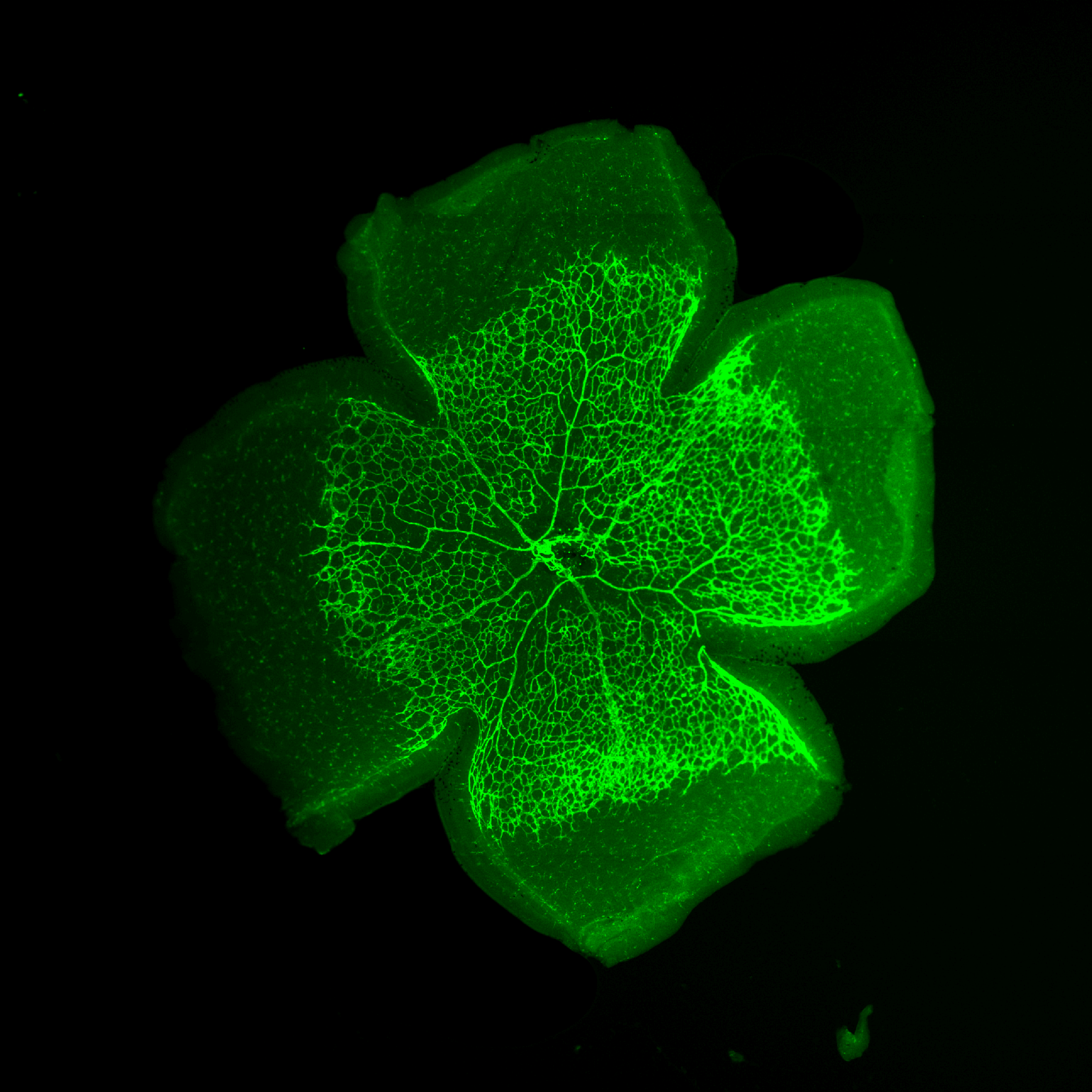

Supplement: Supplementary file 15 — Figure EV4 Source Data [file 44318_2025_642_MOESM15_ESM.zip › EV_4/EV 4F/GlobalNIT2_3_WT.tif]

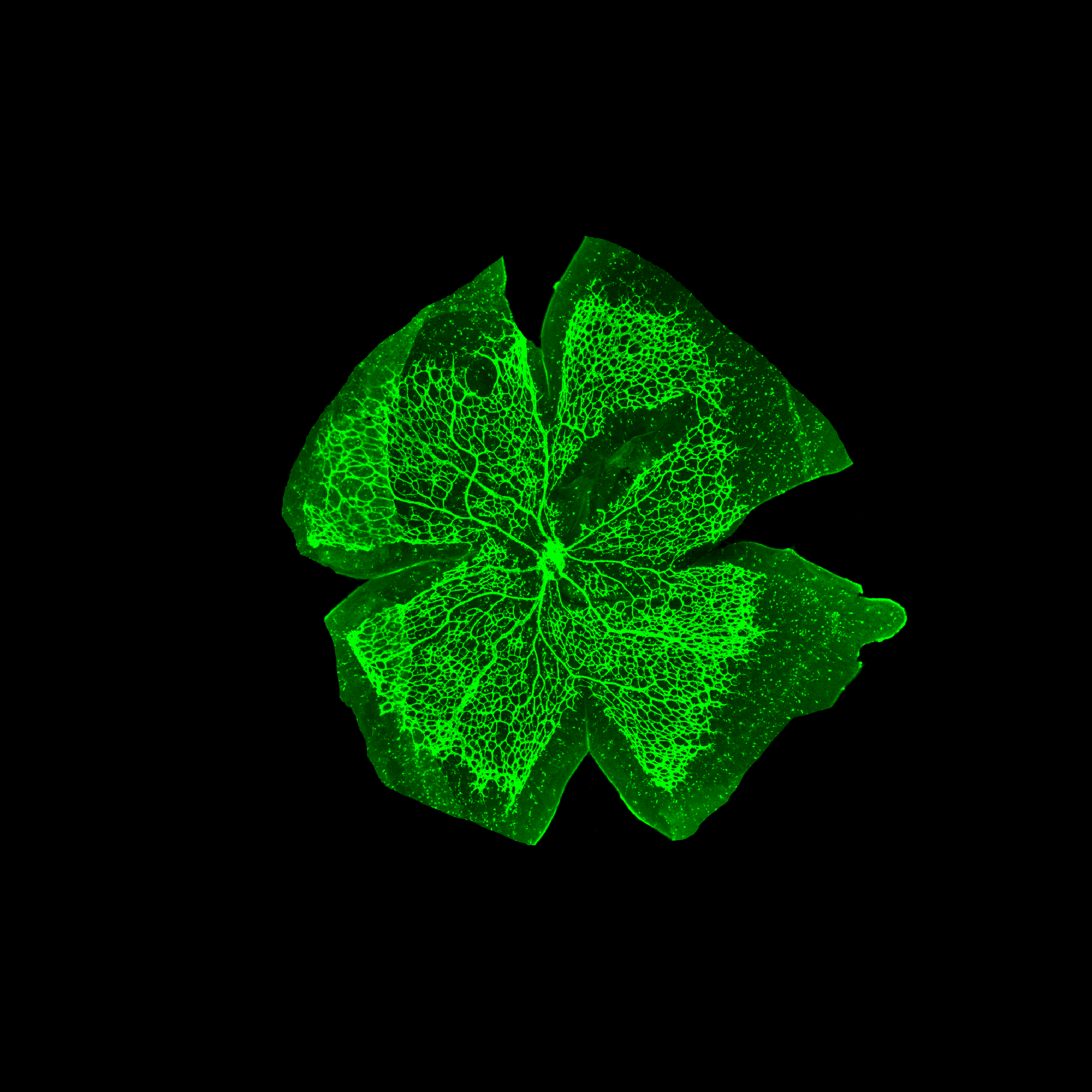

Supplement: Supplementary file 15 — Figure EV4 Source Data [file 44318_2025_642_MOESM15_ESM.zip › EV_4/EV 4F/GlobalNIT2_4_KO.tif]

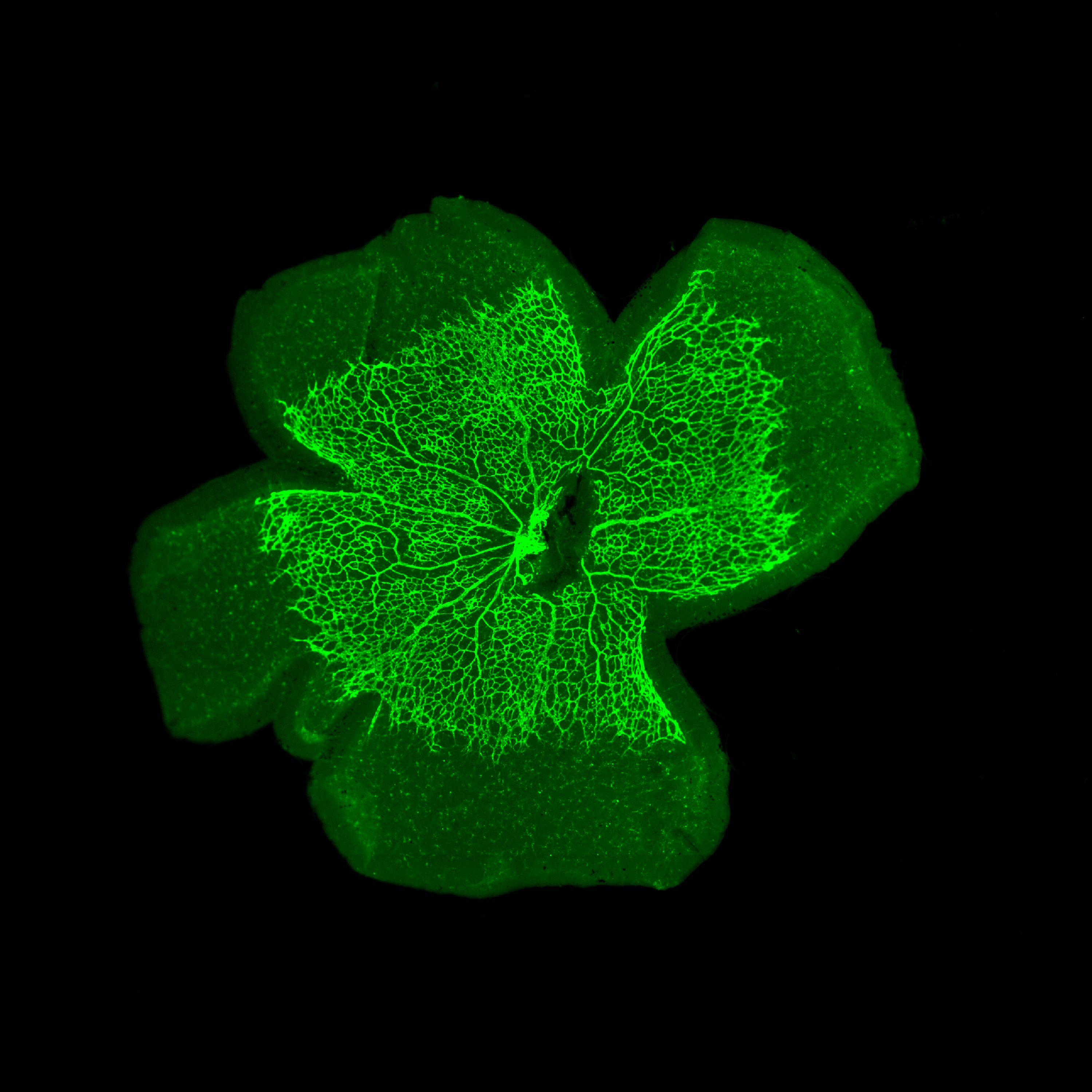

Supplement: Supplementary file 15 — Figure EV4 Source Data [file 44318_2025_642_MOESM15_ESM.zip › EV_4/EV 4F/GlobalNIT2_4_WT.tif]

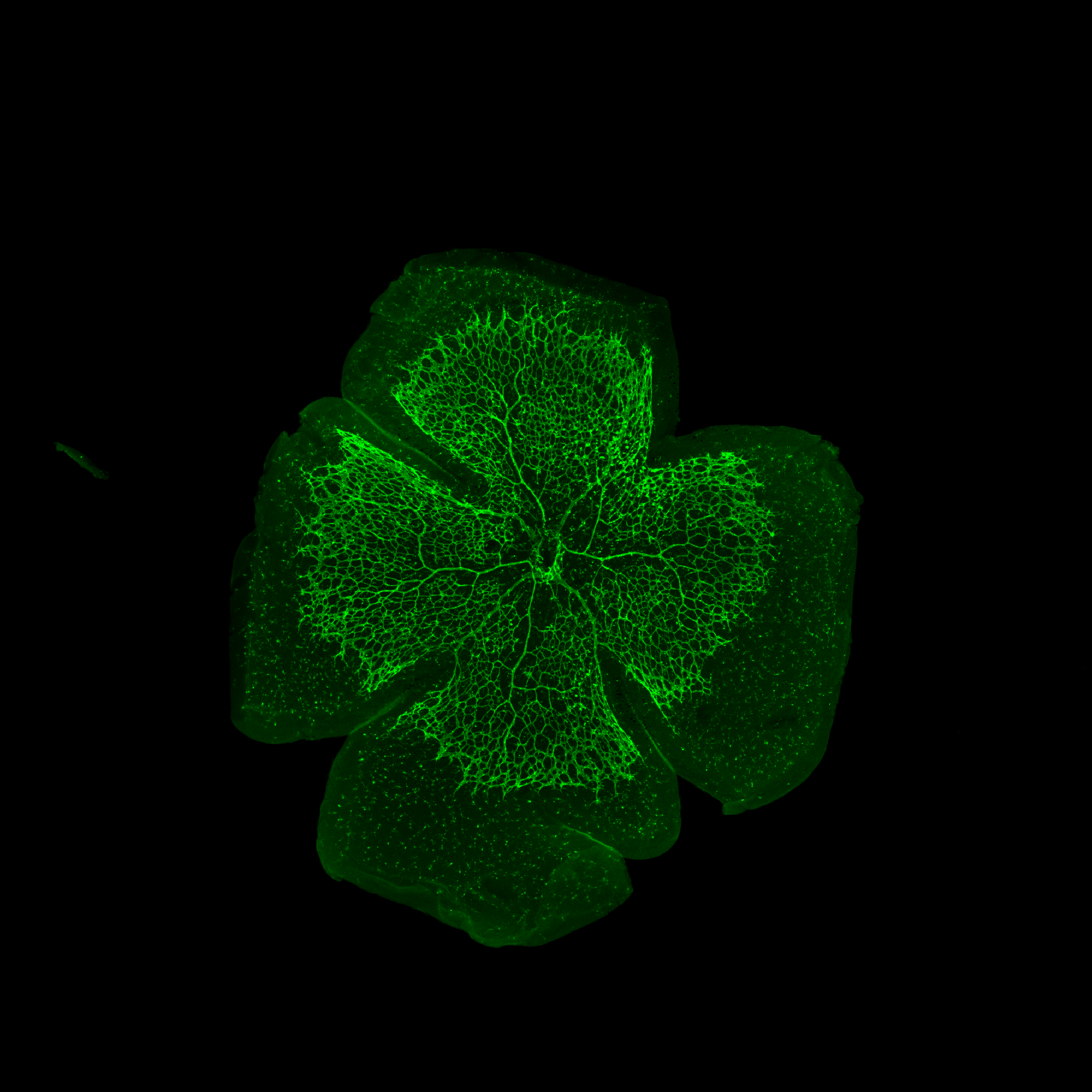

Supplement: Supplementary file 15 — Figure EV4 Source Data [file 44318_2025_642_MOESM15_ESM.zip › EV_4/EV 4F/GlobalNIT2_5_KO.tif]

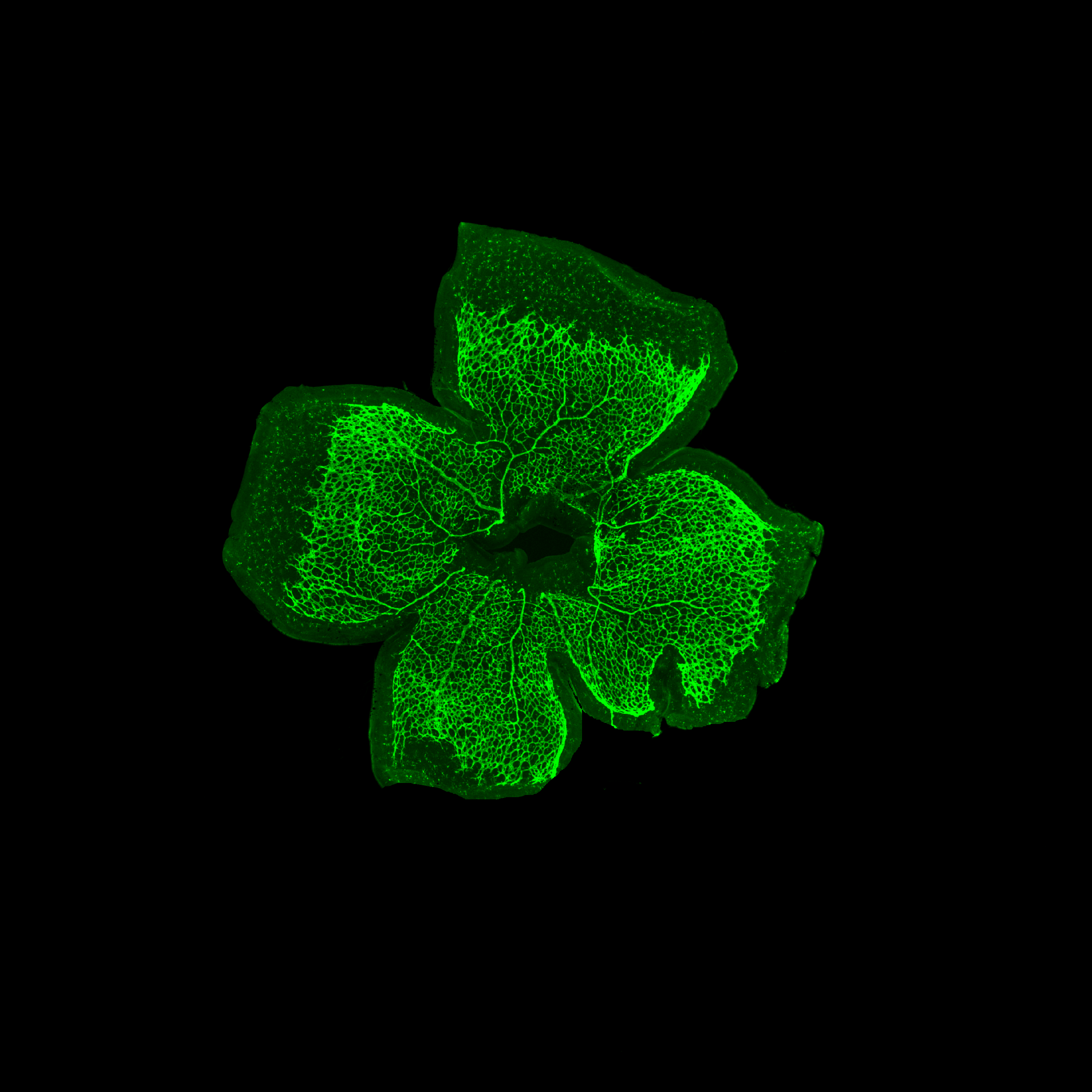

Supplement: Supplementary file 15 — Figure EV4 Source Data [file 44318_2025_642_MOESM15_ESM.zip › EV_4/EV 4F/GlobalNIT2_5_WT.tif]

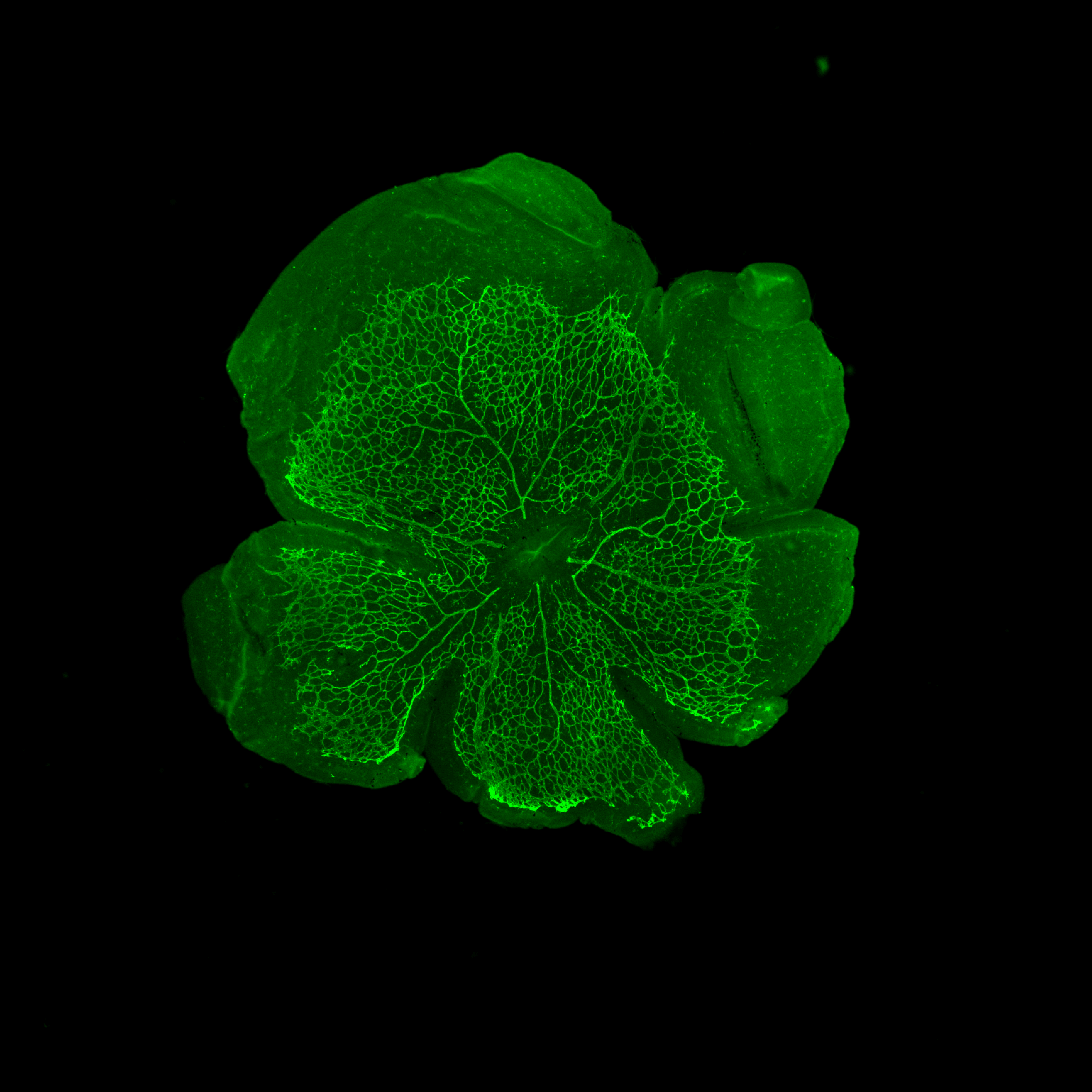

Supplement: Supplementary file 15 — Figure EV4 Source Data [file 44318_2025_642_MOESM15_ESM.zip › EV_4/EV 4F/GlobalNIT2_6_KO.tif]

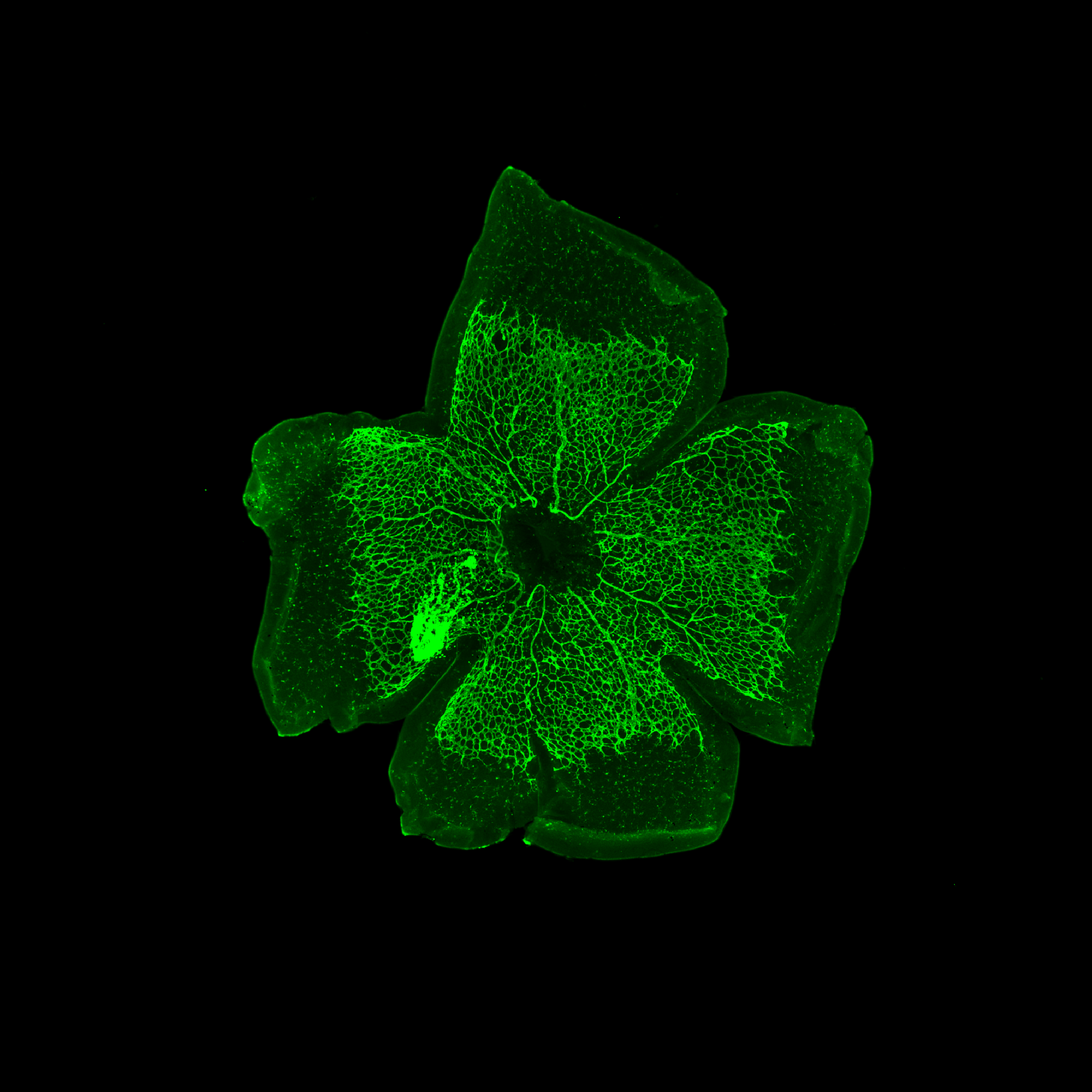

Supplement: Supplementary file 15 — Figure EV4 Source Data [file 44318_2025_642_MOESM15_ESM.zip › EV_4/EV 4F/GlobalNIT2_6_WT.tif]

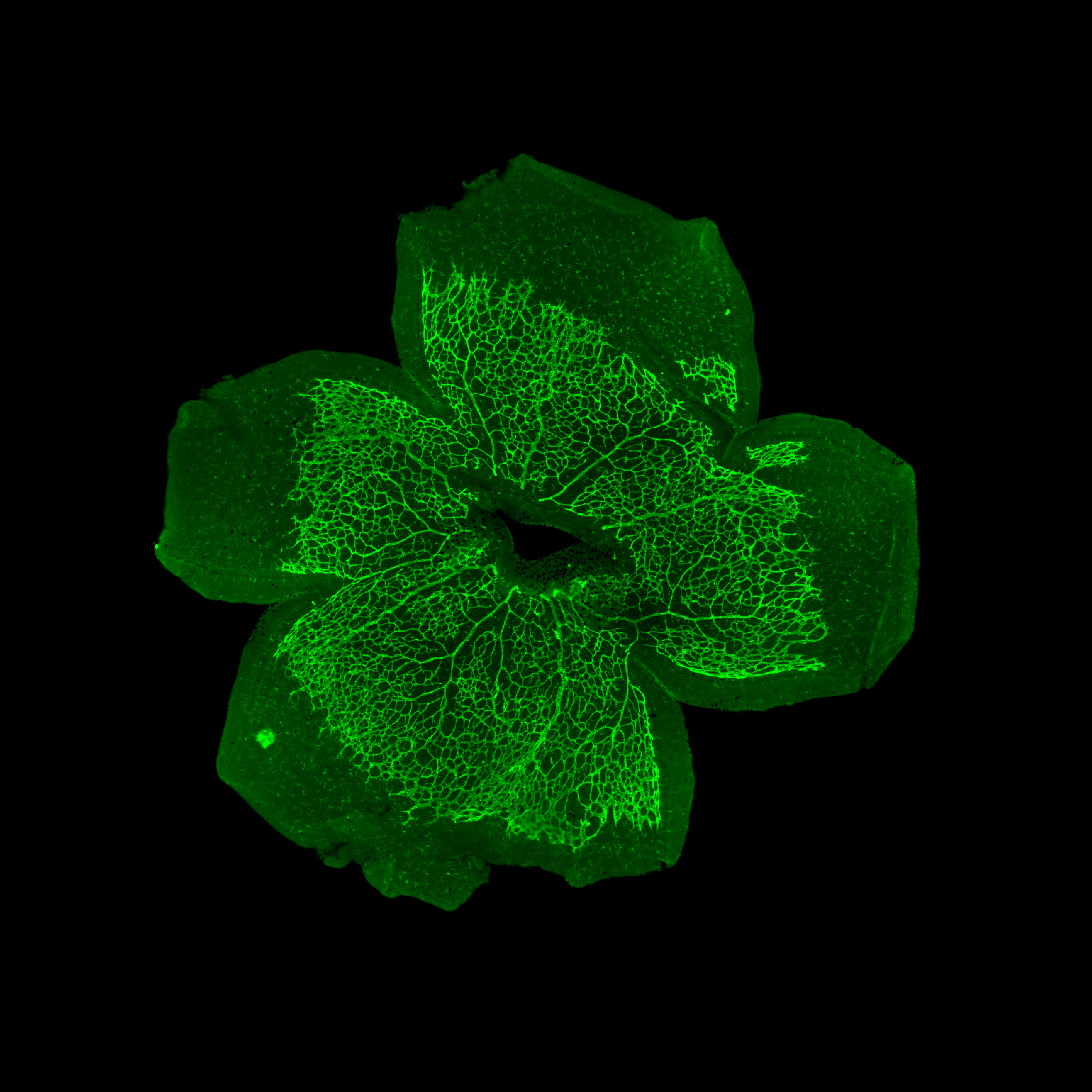

Supplement: Supplementary file 15 — Figure EV4 Source Data [file 44318_2025_642_MOESM15_ESM.zip › EV_4/EV 4F/GlobalNIT2_7_KO.tif]

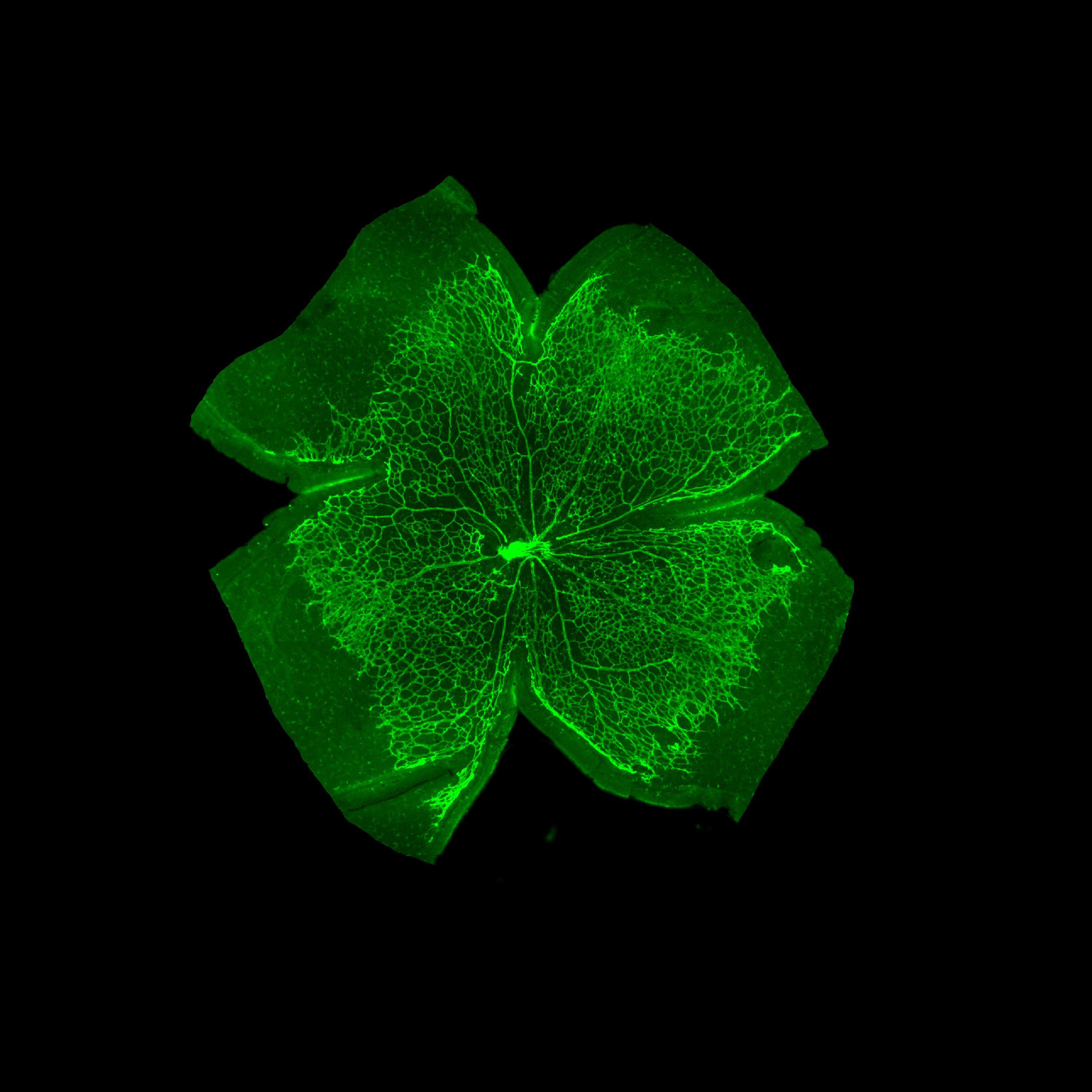

Supplement: Supplementary file 15 — Figure EV4 Source Data [file 44318_2025_642_MOESM15_ESM.zip › EV_4/EV 4F/GlobalNIT2_7_WT.tif]

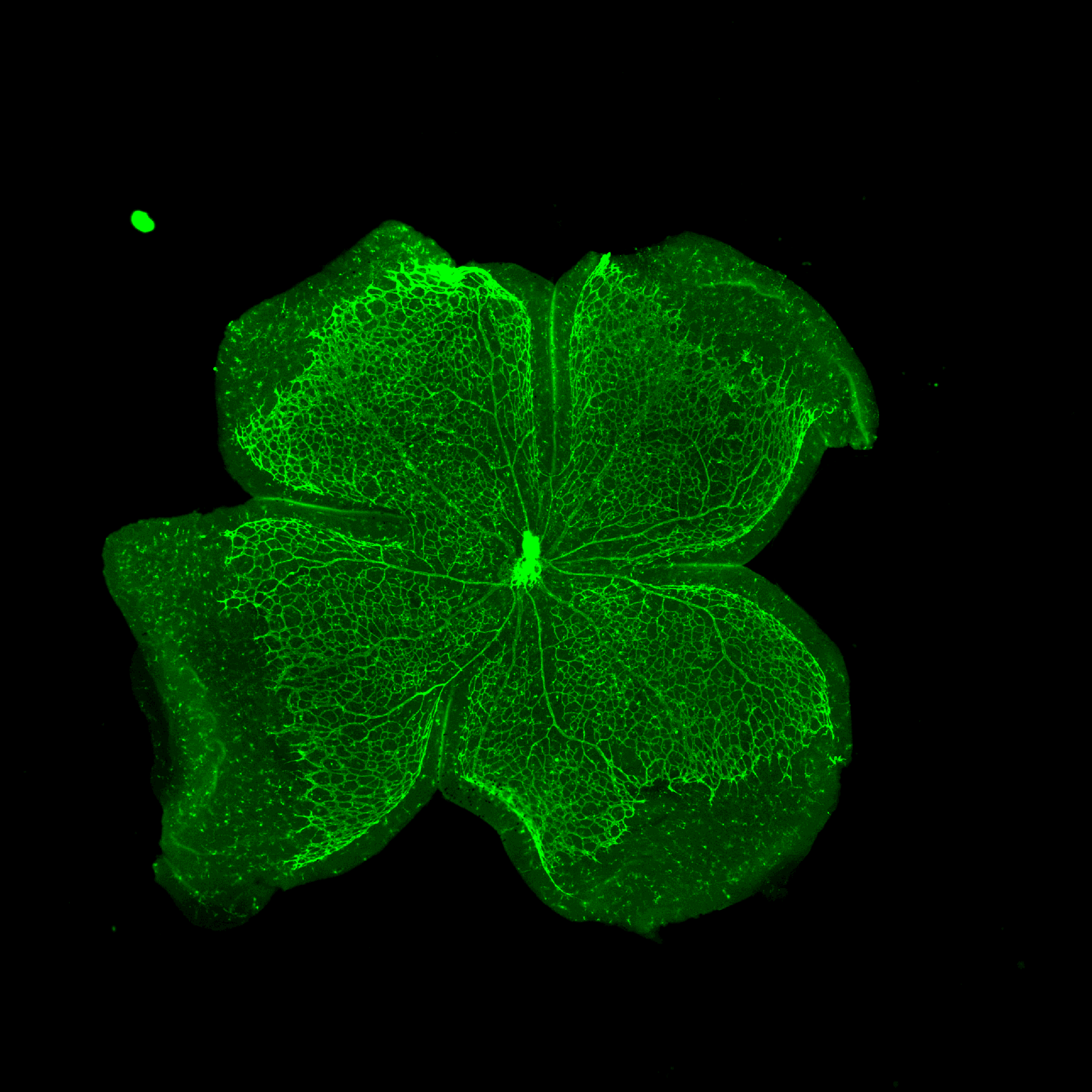

Supplement: Supplementary file 15 — Figure EV4 Source Data [file 44318_2025_642_MOESM15_ESM.zip › EV_4/EV 4F/GlobalNIT2_8_WT.tif]

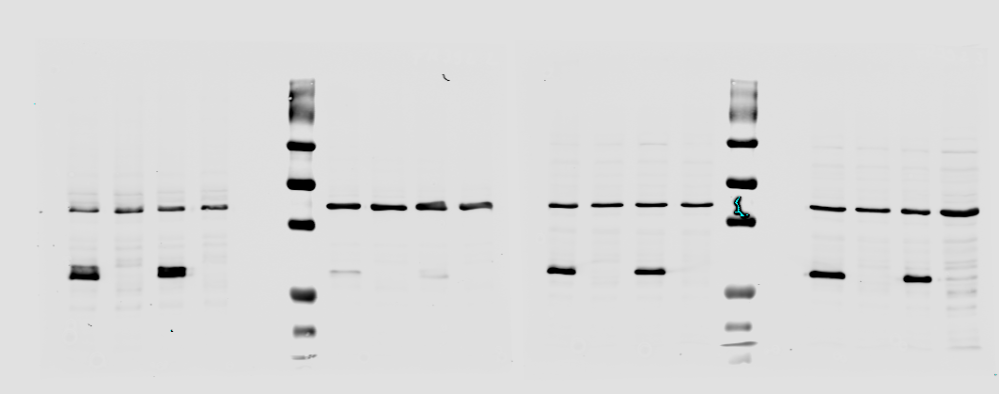

Supplement: Supplementary file 15 — Figure EV4 Source Data [file 44318_2025_642_MOESM15_ESM.zip › EV_4/EV4E Nit2 KO mouse.tif]

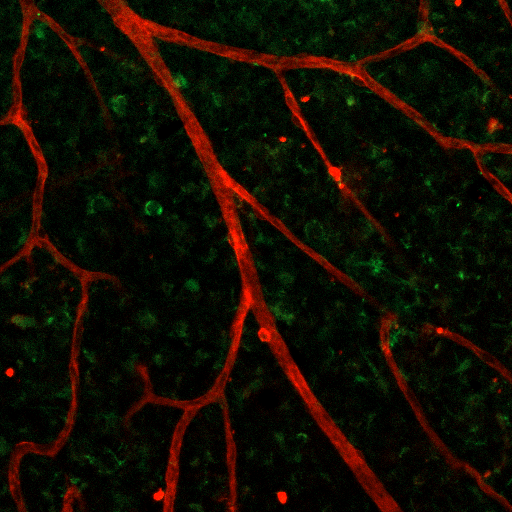

Supplement: Supplementary file 16 — Figure EV6 Source Data [file 44318_2025_642_MOESM16_ESM.zip › EV_6/Fig_EV6/EV_6A/Fig. A_CTL.tif]

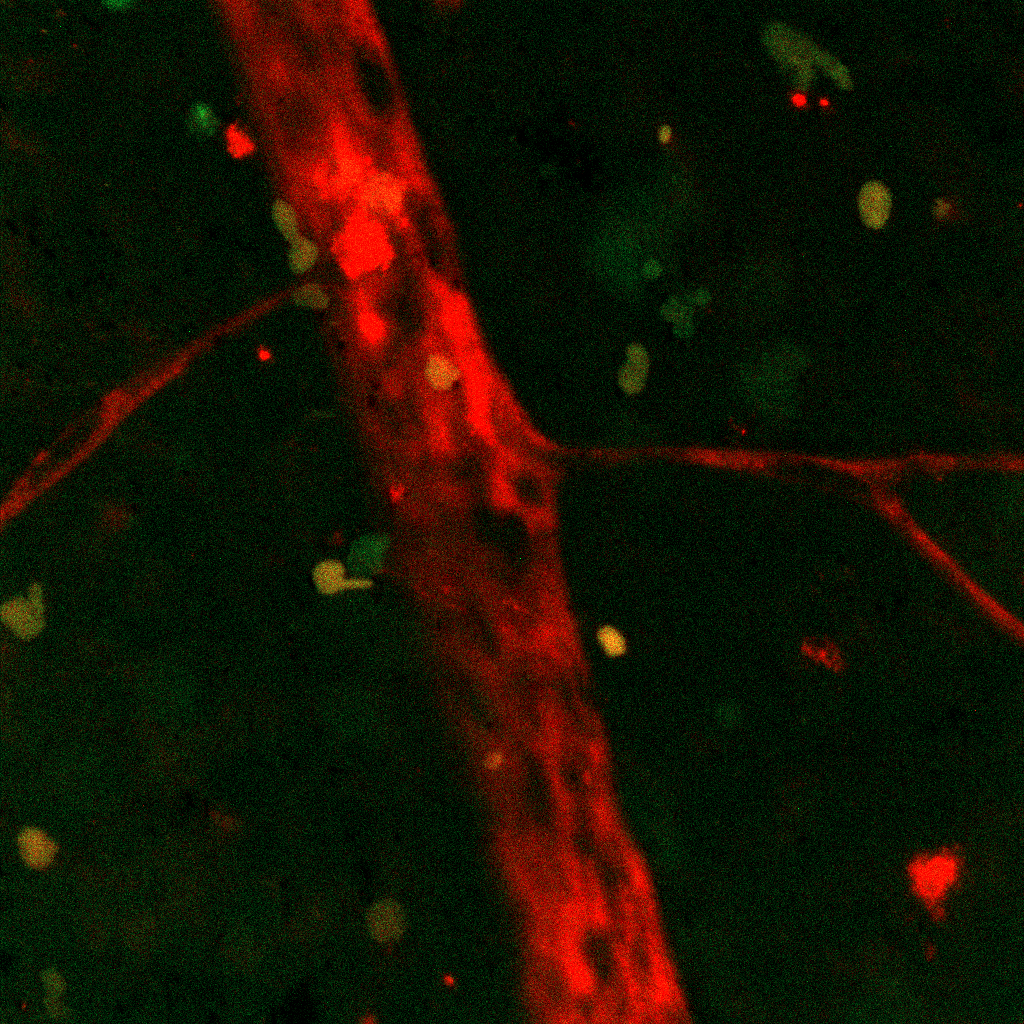

Supplement: Supplementary file 16 — Figure EV6 Source Data [file 44318_2025_642_MOESM16_ESM.zip › EV_6/Fig_EV6/EV_6A/Fig. A_ecNIT2_k.o..tif]

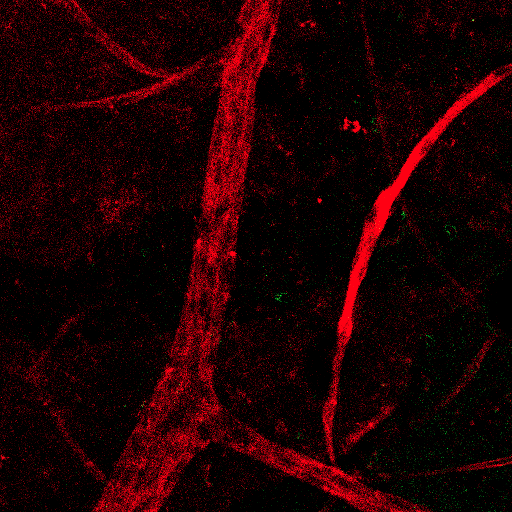

Supplement: Supplementary file 16 — Figure EV6 Source Data [file 44318_2025_642_MOESM16_ESM.zip › EV_6/Fig_EV6/EV_6B/Aged/Fig. B_Aged_C575I6J_no_CellEvent.tif]

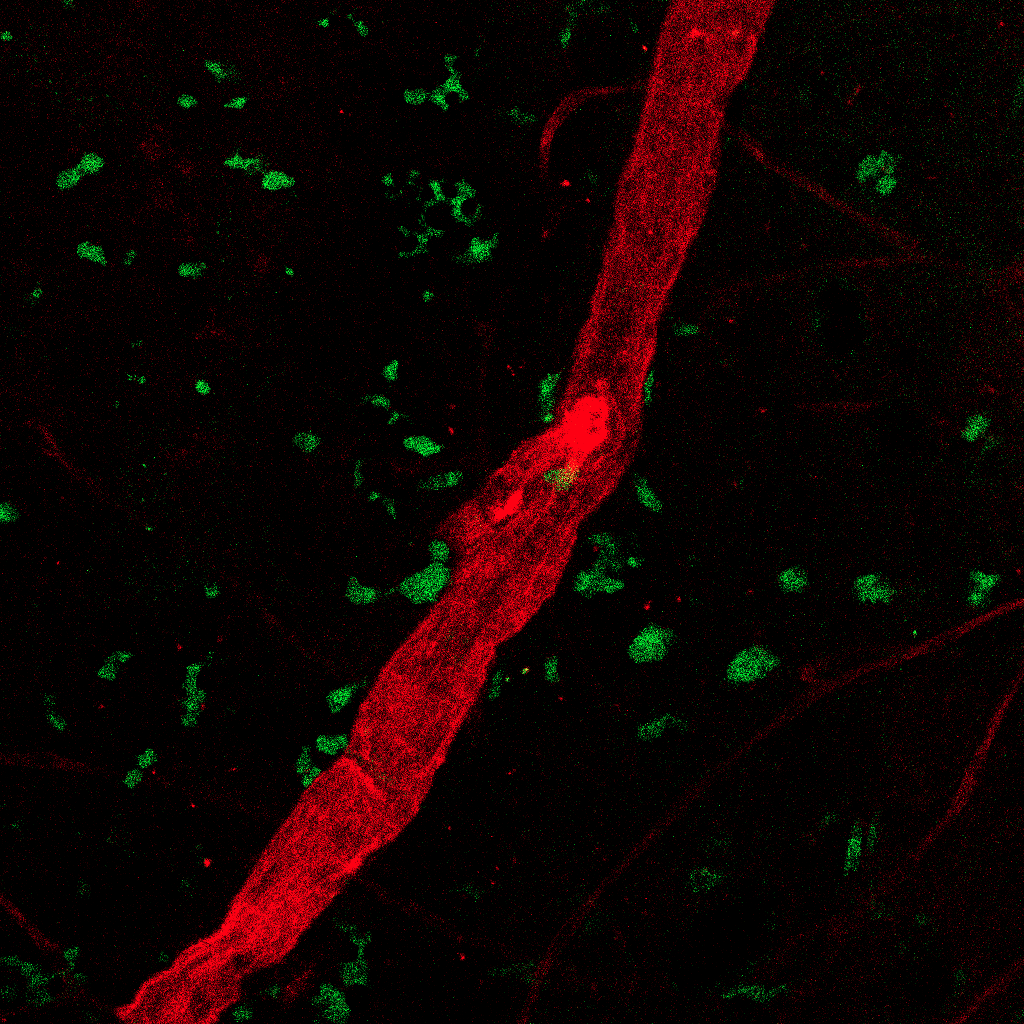

Supplement: Supplementary file 16 — Figure EV6 Source Data [file 44318_2025_642_MOESM16_ESM.zip › EV_6/Fig_EV6/EV_6B/Aged/Fig. B_Aged_Nit2_ko_ko.tif]

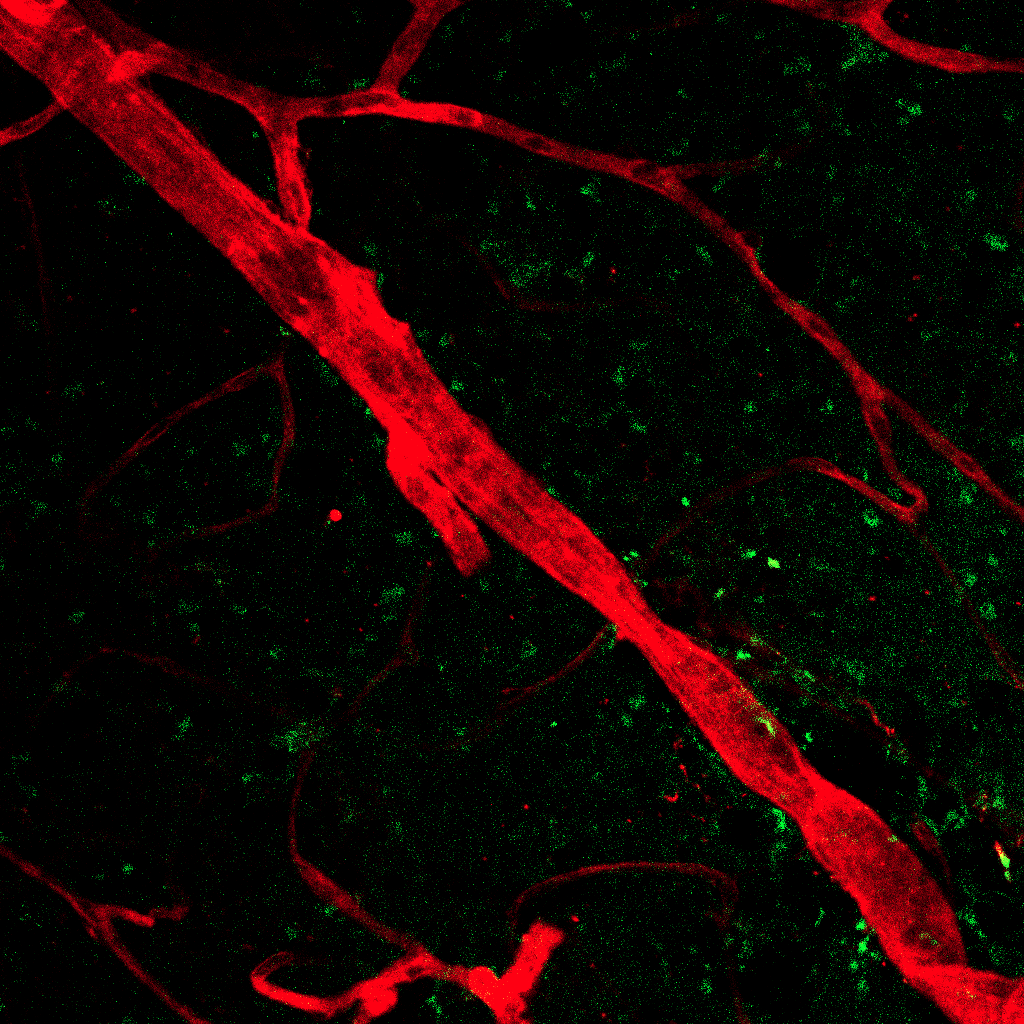

Supplement: Supplementary file 16 — Figure EV6 Source Data [file 44318_2025_642_MOESM16_ESM.zip › EV_6/Fig_EV6/EV_6B/Aged/Fig. B_Aged_Nit2_wt_wt.tif]

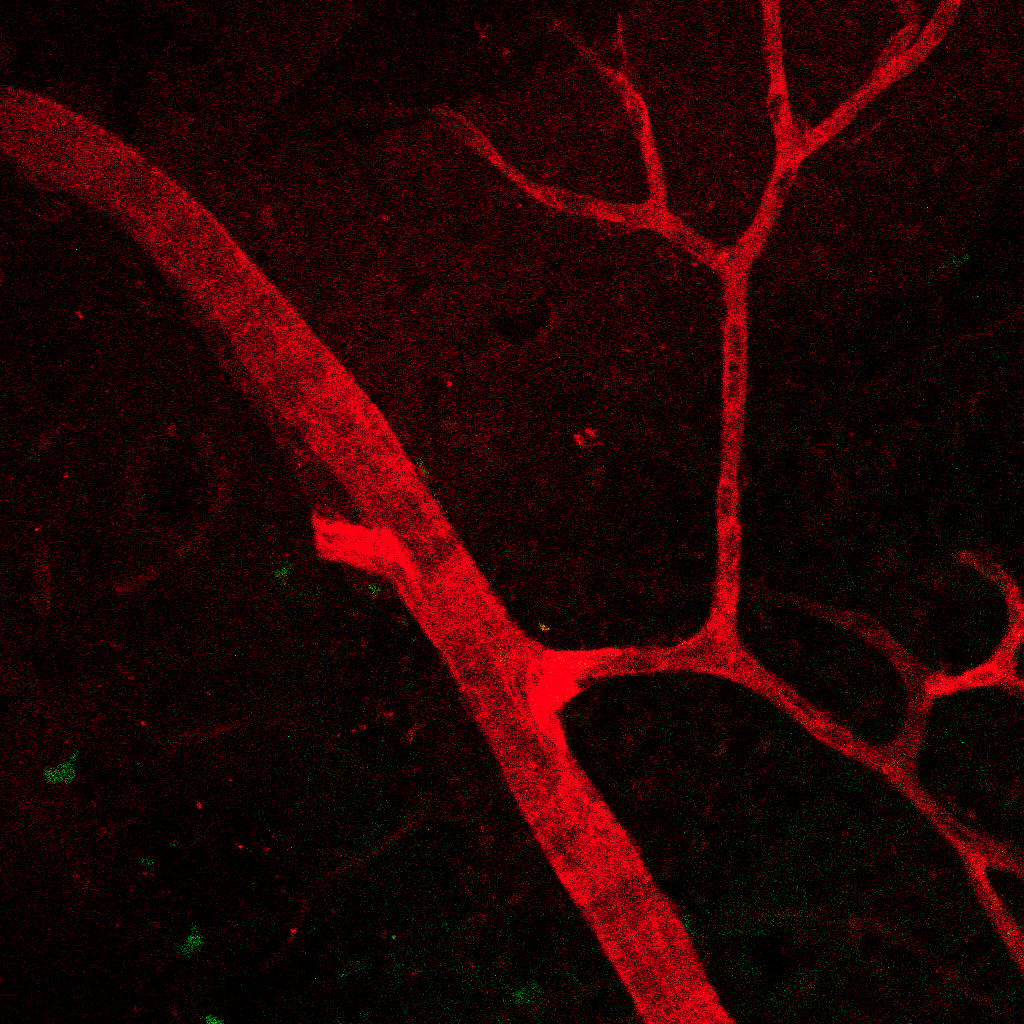

Supplement: Supplementary file 16 — Figure EV6 Source Data [file 44318_2025_642_MOESM16_ESM.zip › EV_6/Fig_EV6/EV_6B/Young/Fig. B_Young_C575I6J_no_CellEvent.tif]

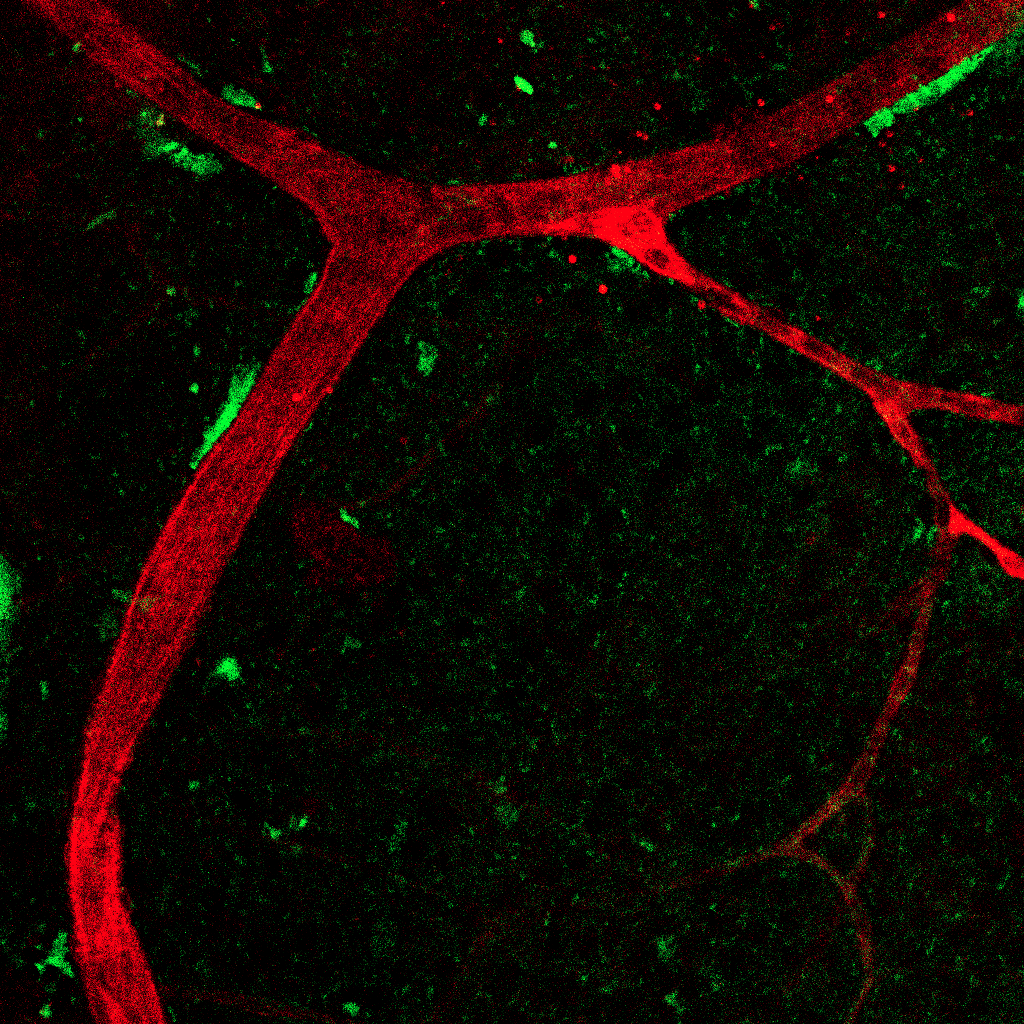

Supplement: Supplementary file 16 — Figure EV6 Source Data [file 44318_2025_642_MOESM16_ESM.zip › EV_6/Fig_EV6/EV_6B/Young/Fig. B_Young_Nit2_ko_ko.tif]

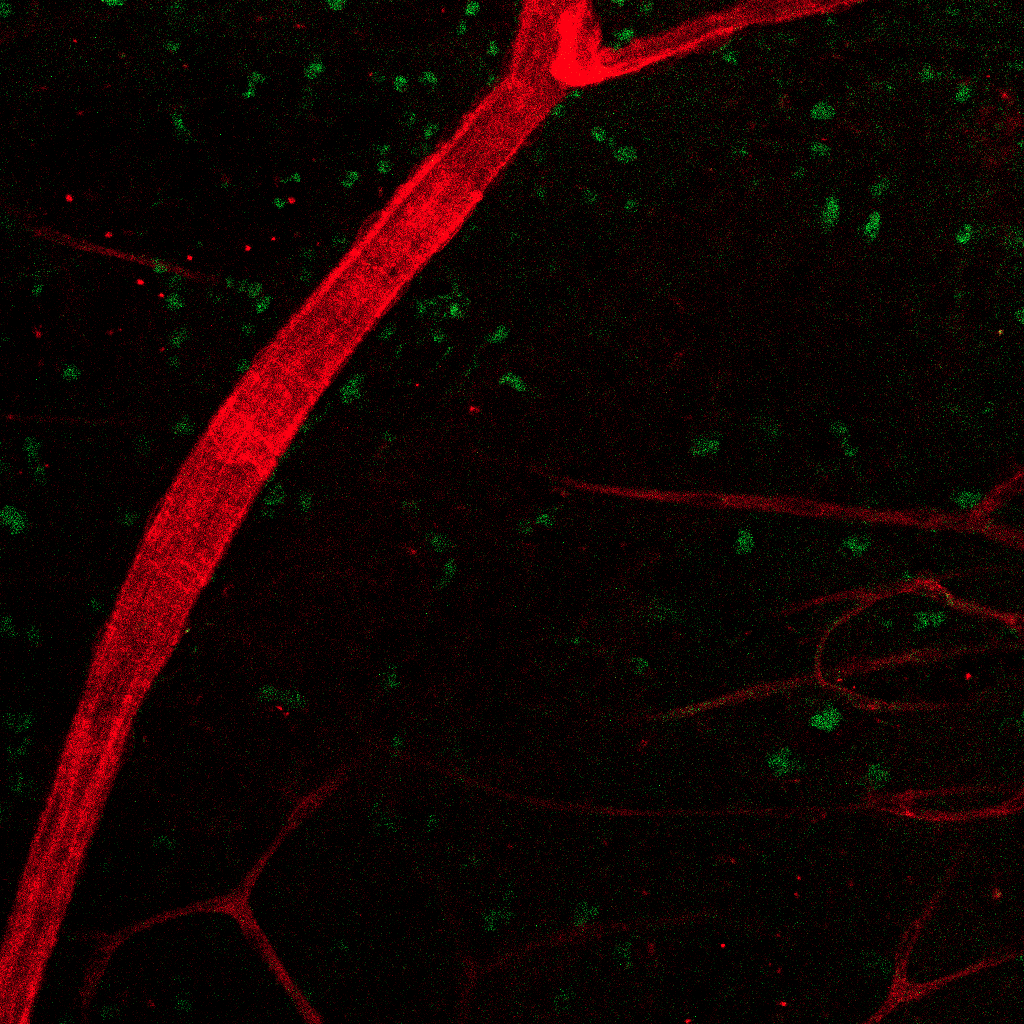

Supplement: Supplementary file 16 — Figure EV6 Source Data [file 44318_2025_642_MOESM16_ESM.zip › EV_6/Fig_EV6/EV_6B/Young/Fig. B_Young_Nit2_wt_wt.tif]

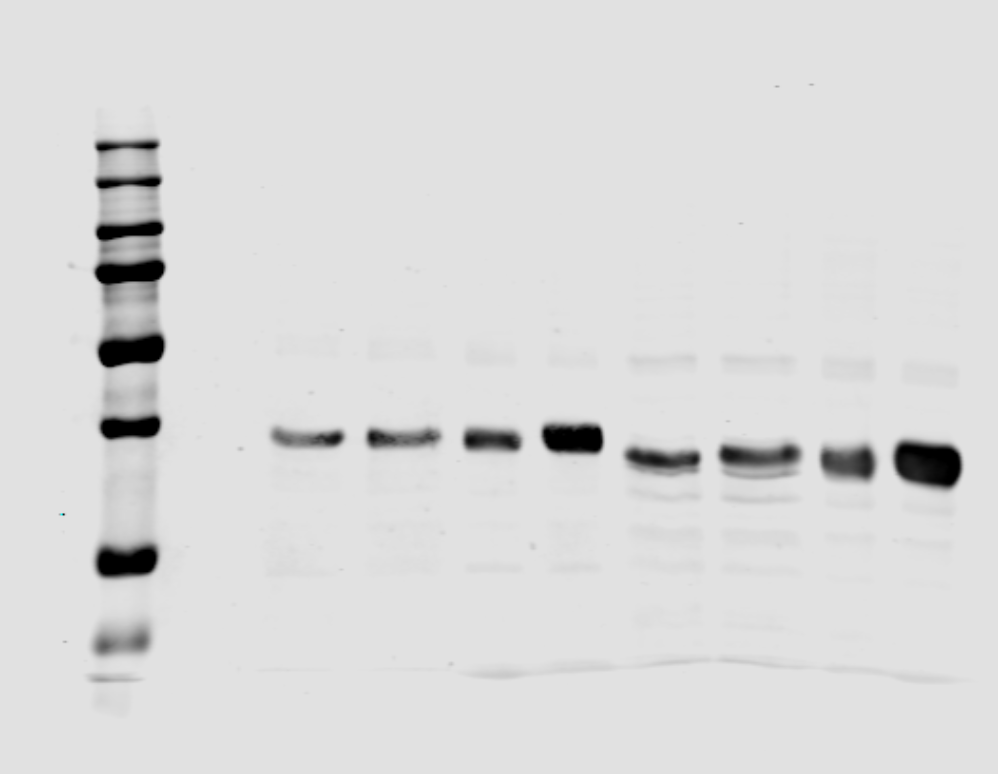

Supplement: Supplementary file 17 — Figure EV7 Source Data [file 44318_2025_642_MOESM17_ESM.zip › EV_7/EV 7 D and E_NM284 SMC Fib GAPDH.tif]

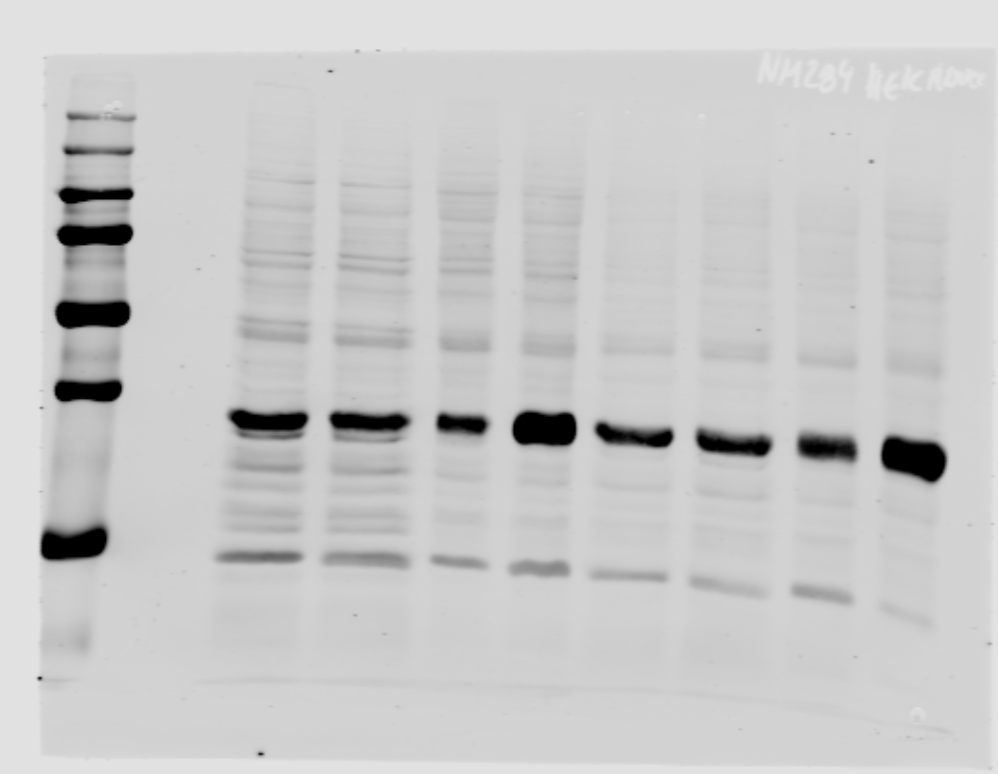

Supplement: Supplementary file 17 — Figure EV7 Source Data [file 44318_2025_642_MOESM17_ESM.zip › EV_7/EV 7A and F_NM284 HEK HUVEC GAPDH.tif]

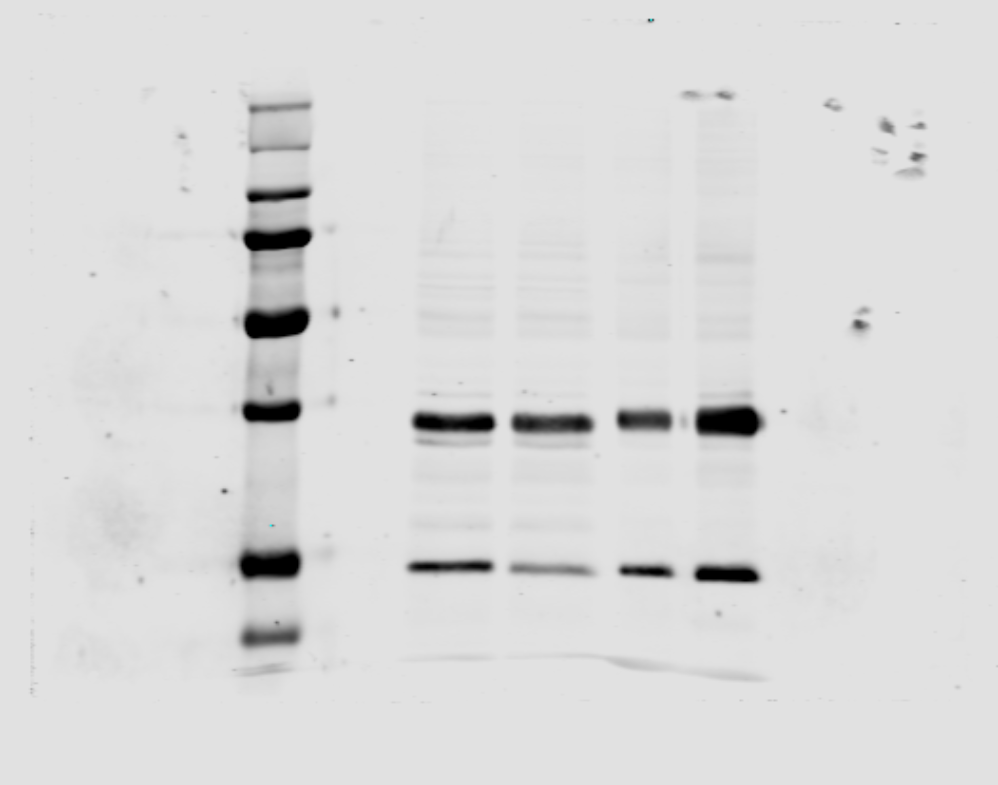

Supplement: Supplementary file 17 — Figure EV7 Source Data [file 44318_2025_642_MOESM17_ESM.zip › EV_7/EV 7B.tif]

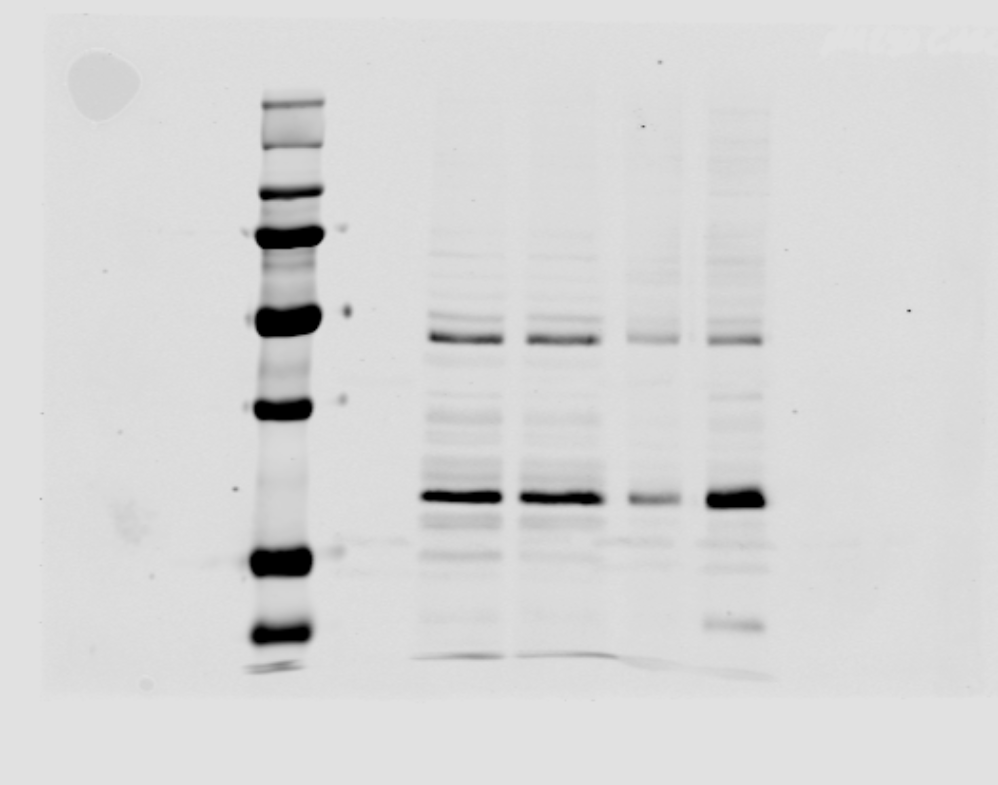

Supplement: Supplementary file 17 — Figure EV7 Source Data [file 44318_2025_642_MOESM17_ESM.zip › EV_7/EV 7B_NM290 hCAEC.tif]

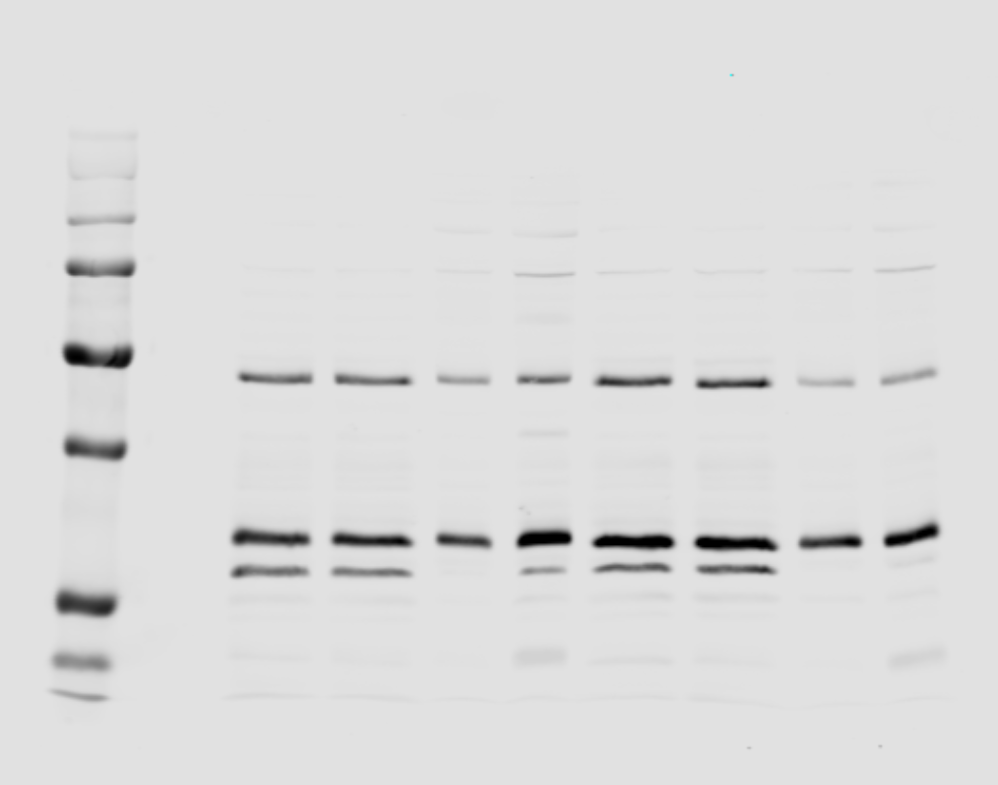

Supplement: Supplementary file 17 — Figure EV7 Source Data [file 44318_2025_642_MOESM17_ESM.zip › EV_7/EV 7C_NM290 huvec haec nit2.tif]

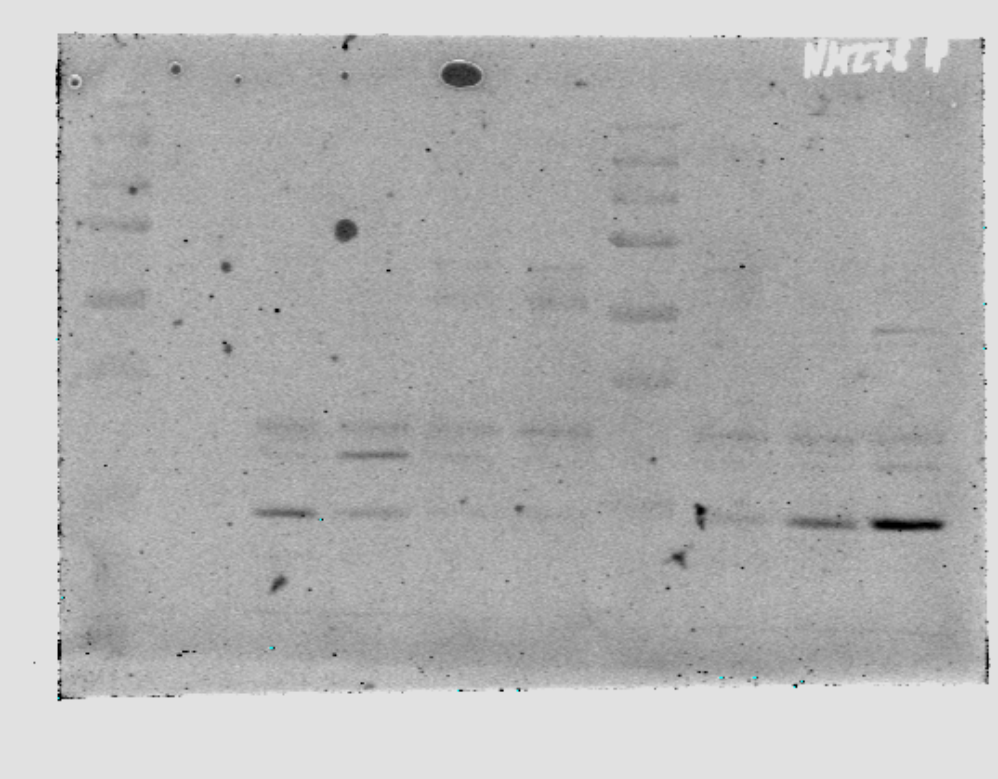

Supplement: Supplementary file 17 — Figure EV7 Source Data [file 44318_2025_642_MOESM17_ESM.zip › EV_7/EV 7G.tif]

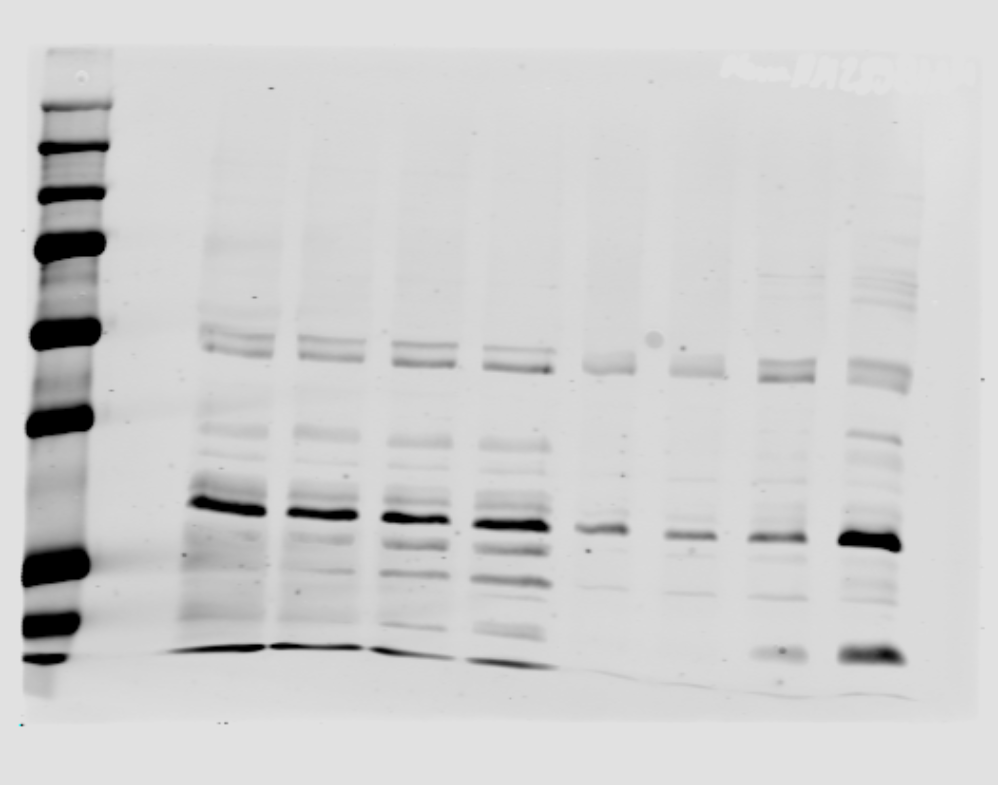

Supplement: Supplementary file 17 — Figure EV7 Source Data [file 44318_2025_642_MOESM17_ESM.zip › EV_7/EV 7H.tif]

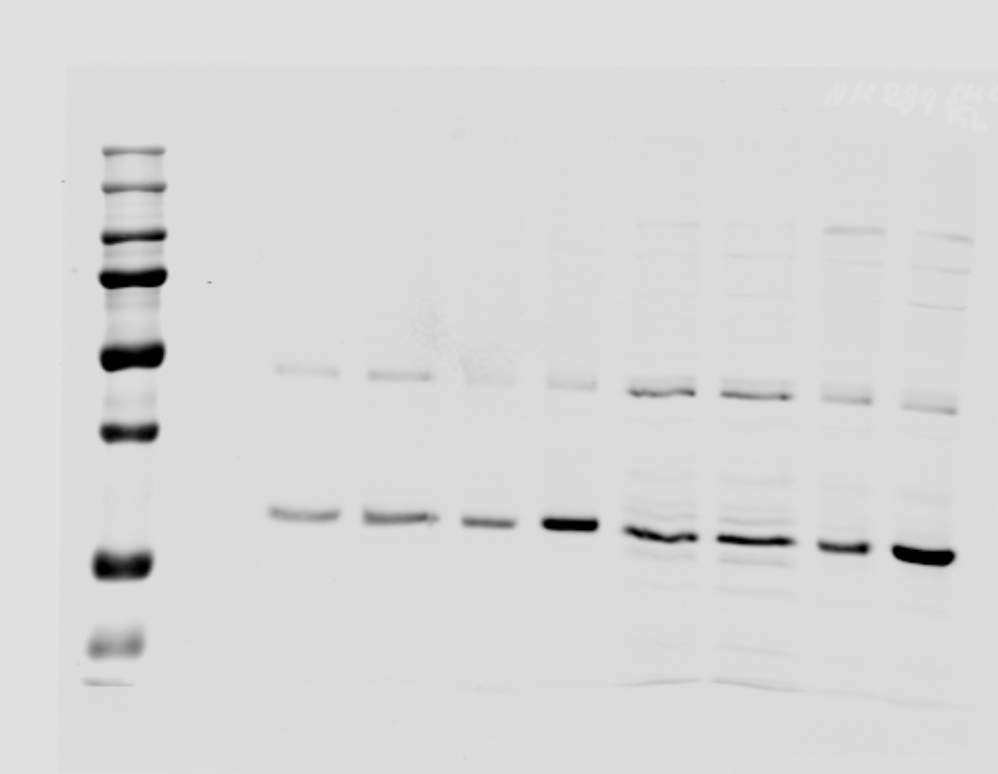

Supplement: Supplementary file 17 — Figure EV7 Source Data [file 44318_2025_642_MOESM17_ESM.zip › EV_7/EV7 D and E_NM284 SMC Fib NIT2.tif]

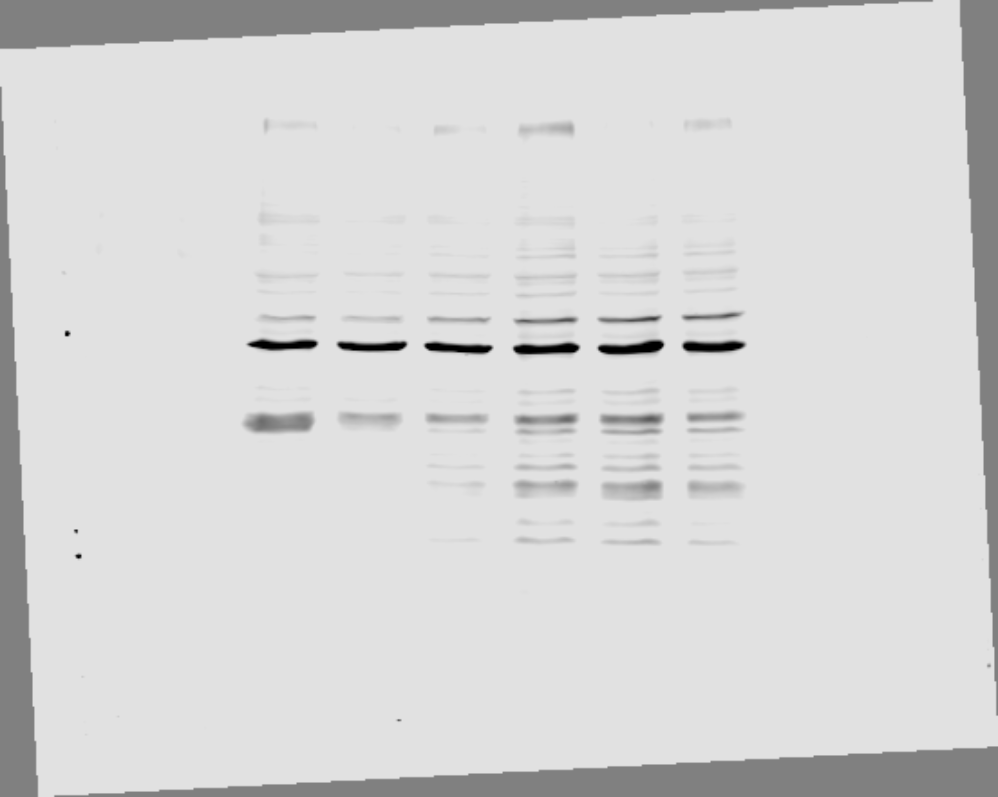

Supplement: Supplementary file 18 — Figure EV9 Source Data [file 44318_2025_642_MOESM18_ESM.zip › EV_9/EV 9A_beta actin_NM237_NIT2 mutants.tif]

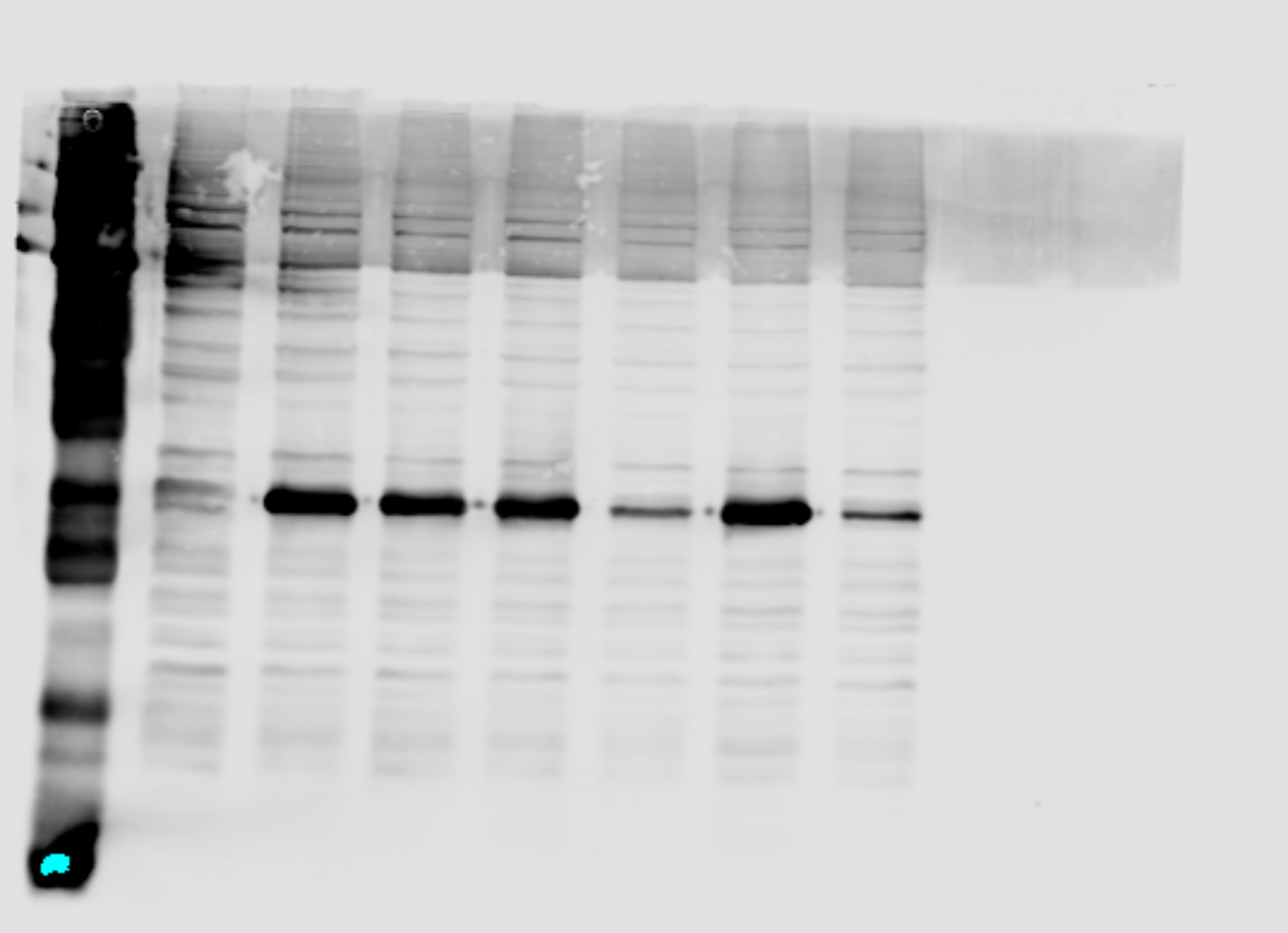

Supplement: Supplementary file 18 — Figure EV9 Source Data [file 44318_2025_642_MOESM18_ESM.zip › EV_9/EV 9A_NM233_his tag AB.tif]
