# Supplementary material for: The transaminase-ω-amidase pathway senses oxidative stress to control glutamine metabolism and α-ketoglutarate levels in endothelial cells
Source: EMBO J. 2025 Dec 17;45(3):820–55. doi: 10.1038/s44318-025-00642-7 (PMC12864753; doi:10.1038/s44318-025-00642-7)
Supplement: Supplementary file 11 — Source data Fig. 4 [file 44318_2025_642_MOESM11_ESM.zip › Figure 4/Fig. 4A.pptx]

## Slide 1
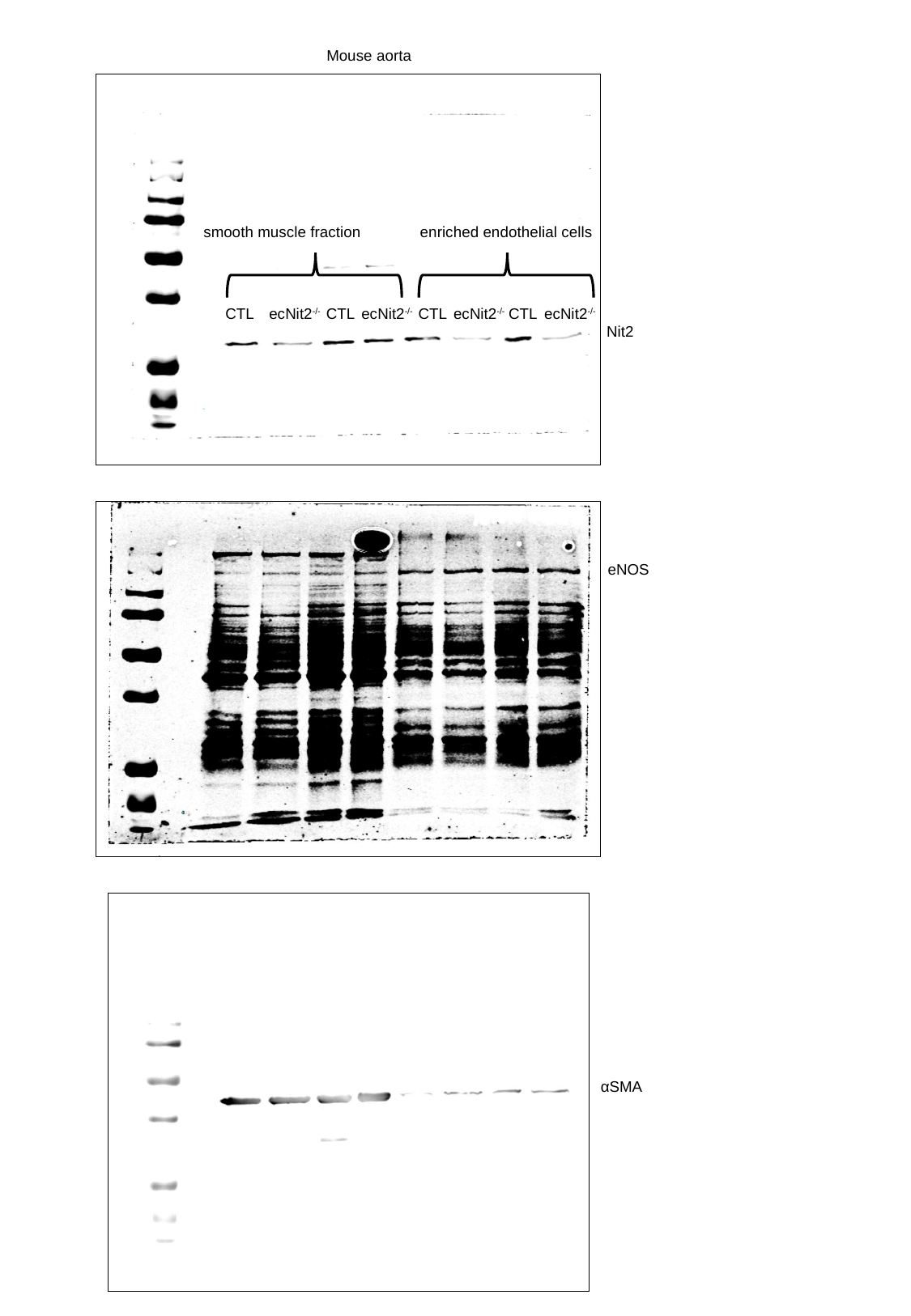

Mouse aorta
smooth muscle fraction
enriched endothelial cells
CTL
ecNit2-/-
CTL
CTL
ecNit2-/-
CTL
ecNit2-/-
ecNit2-/-
Nit2
eNOS
αSMA
