# Supplementary material for: The transaminase-ω-amidase pathway senses oxidative stress to control glutamine metabolism and α-ketoglutarate levels in endothelial cells
Source: EMBO J. 2025 Dec 17;45(3):820–55. doi: 10.1038/s44318-025-00642-7 (PMC12864753; doi:10.1038/s44318-025-00642-7)
Supplement: Supplementary file 11 — Source data Fig. 4 [file 44318_2025_642_MOESM11_ESM.zip › Figure 4/Fig. 4L.pptx]

## Slide 1
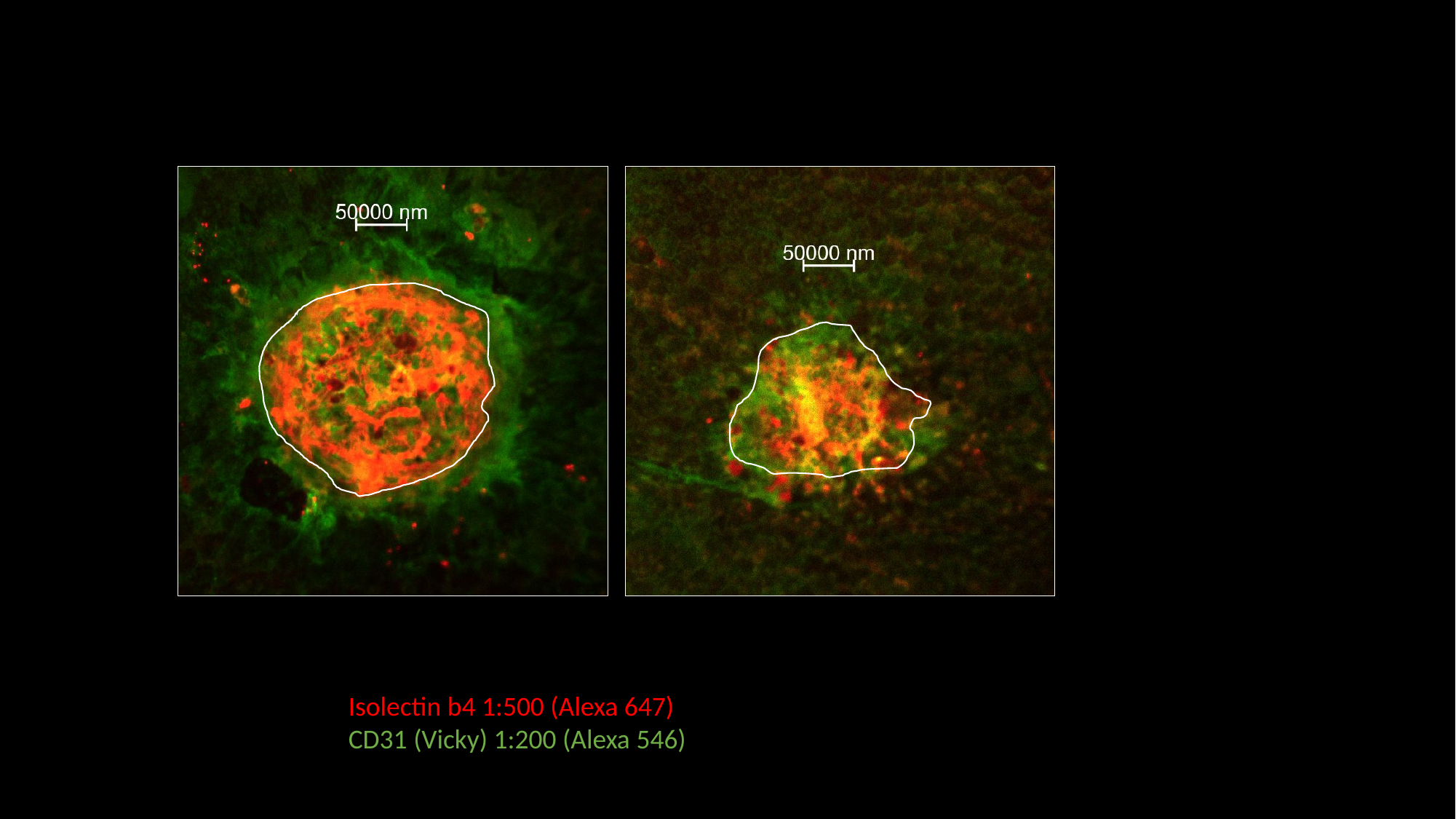

Isolectin b4 1:500 (Alexa 647)
CD31 (Vicky) 1:200 (Alexa 546)

## Slide 2
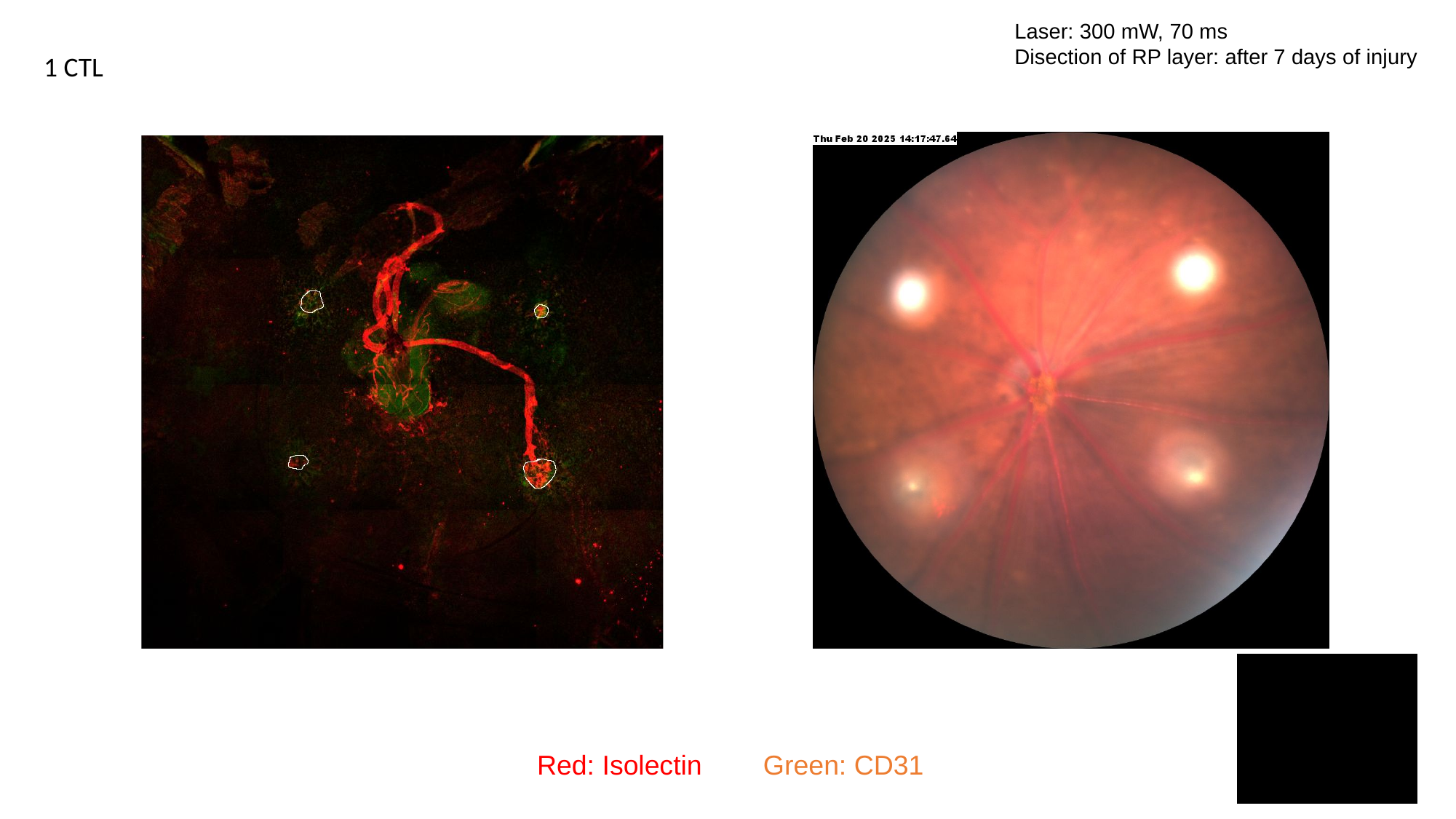

Laser: 300 mW, 70 ms
Disection of RP layer: after 7 days of injury
1 CTL
Red: Isolectin Green: CD31

## Slide 3
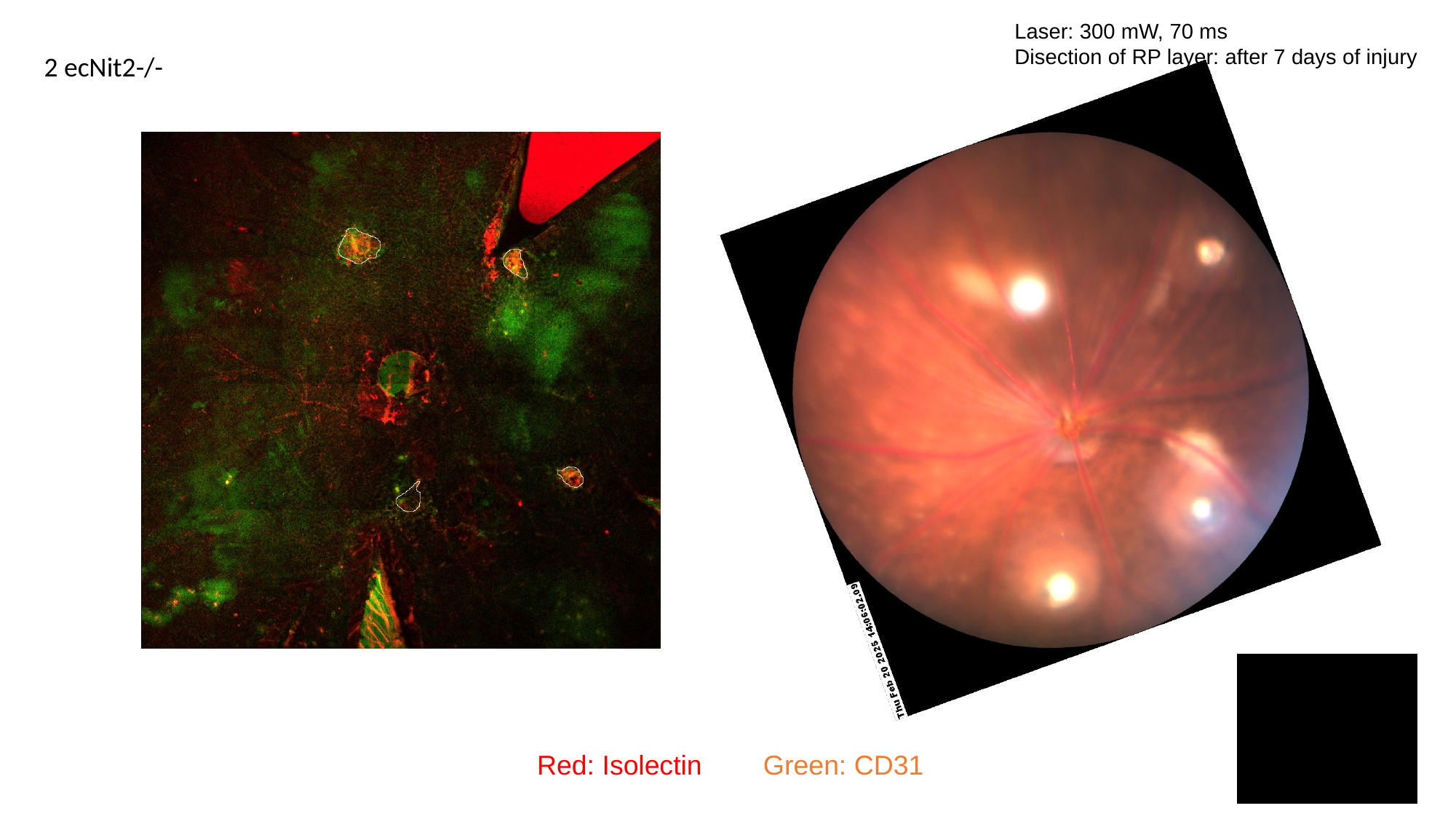

Laser: 300 mW, 70 ms
Disection of RP layer: after 7 days of injury
2 ecNit2-/-
Red: Isolectin Green: CD31

## Slide 4
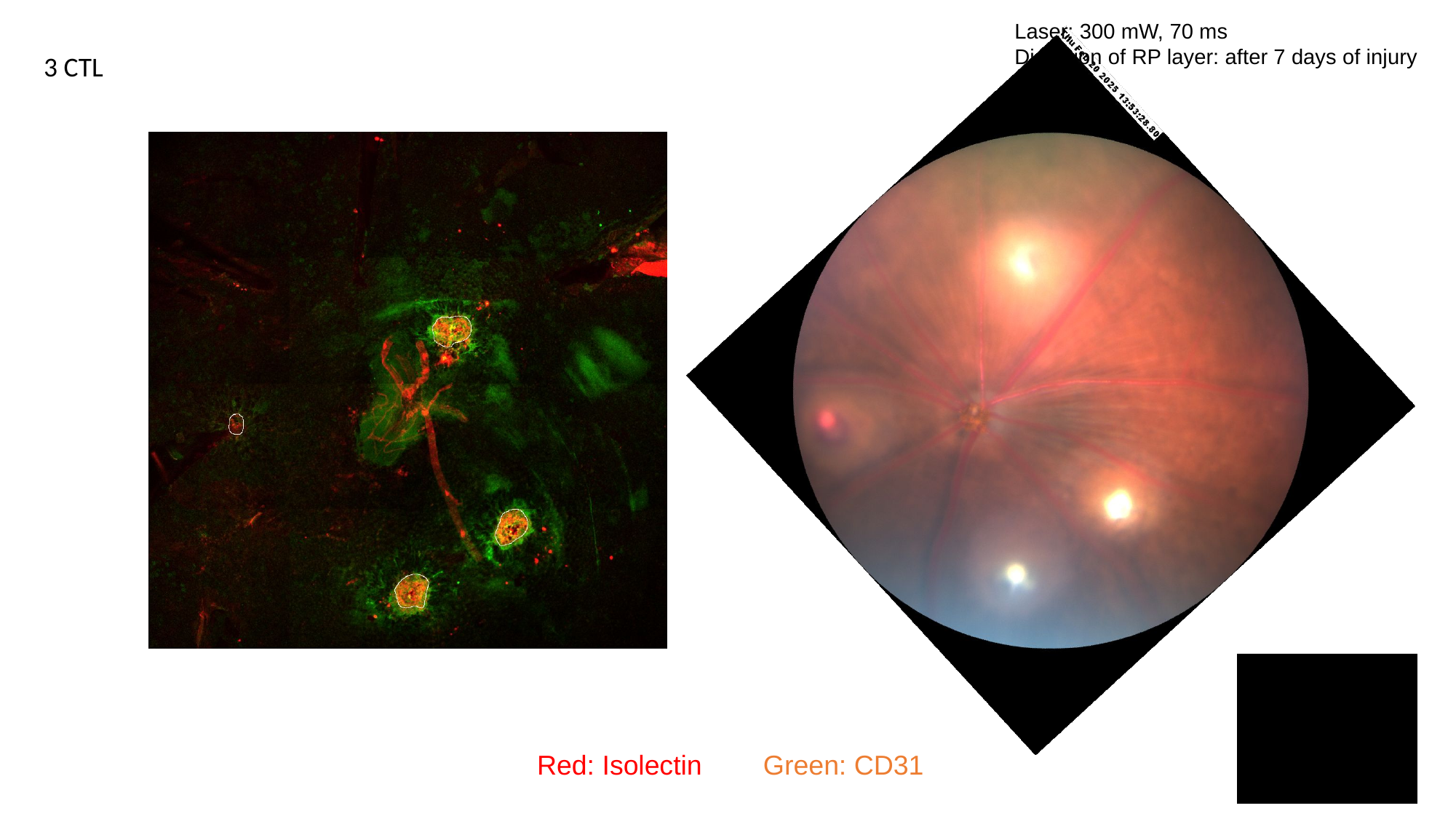

Laser: 300 mW, 70 ms
Disection of RP layer: after 7 days of injury
3 CTL
Red: Isolectin Green: CD31

## Slide 5
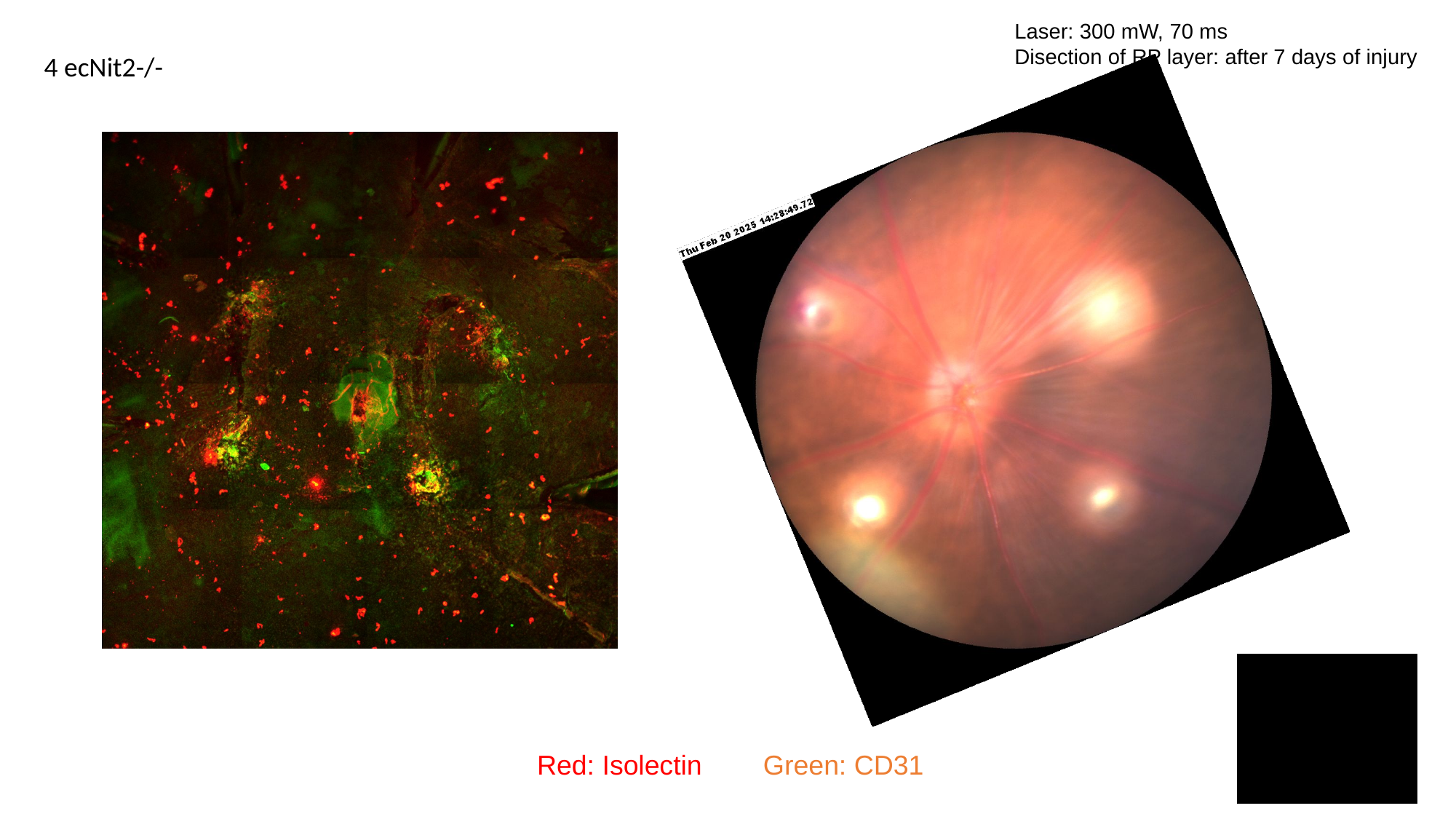

Laser: 300 mW, 70 ms
Disection of RP layer: after 7 days of injury
4 ecNit2-/-
Red: Isolectin Green: CD31

## Slide 6
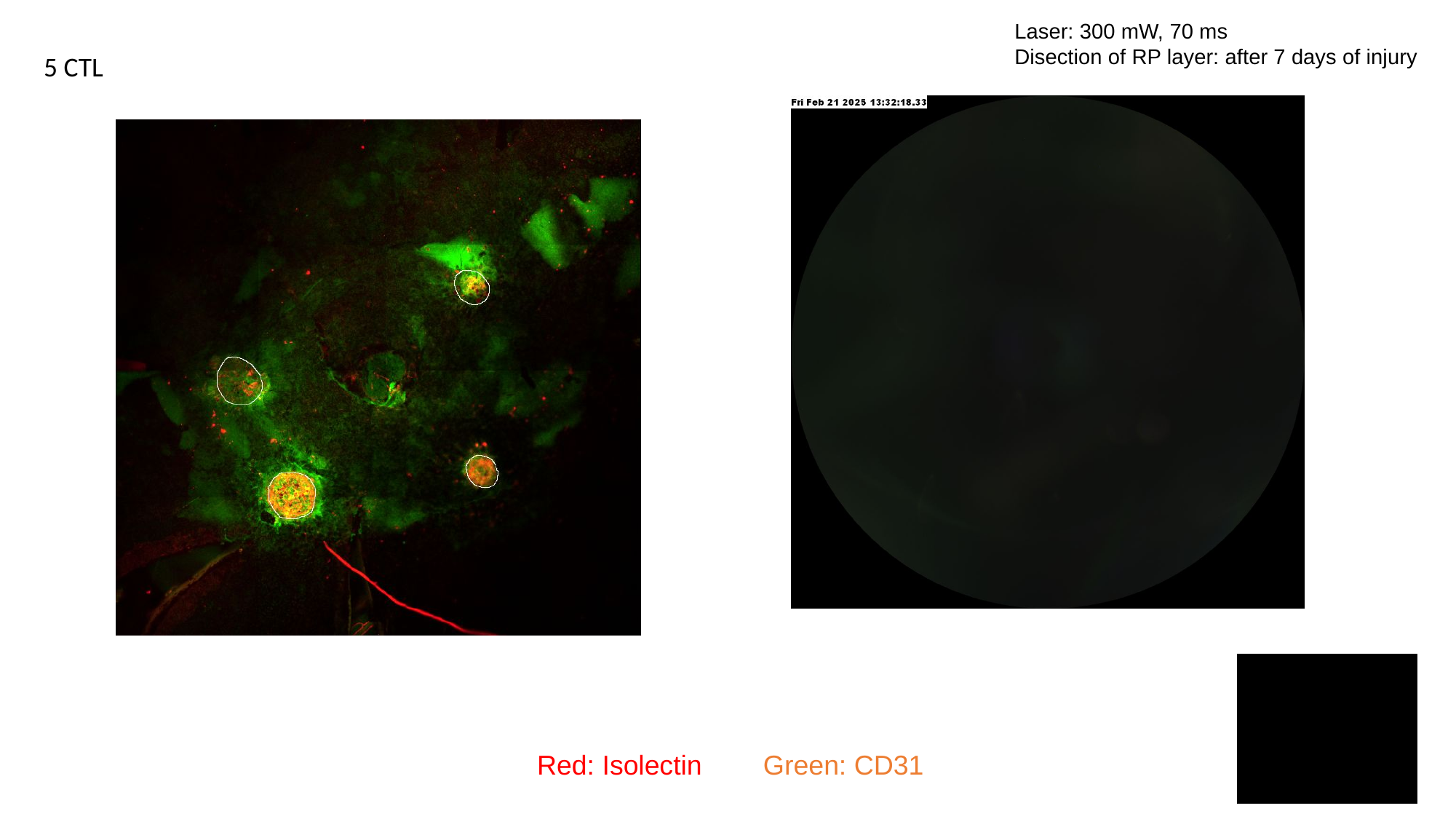

Laser: 300 mW, 70 ms
Disection of RP layer: after 7 days of injury
5 CTL
Red: Isolectin Green: CD31

## Slide 7
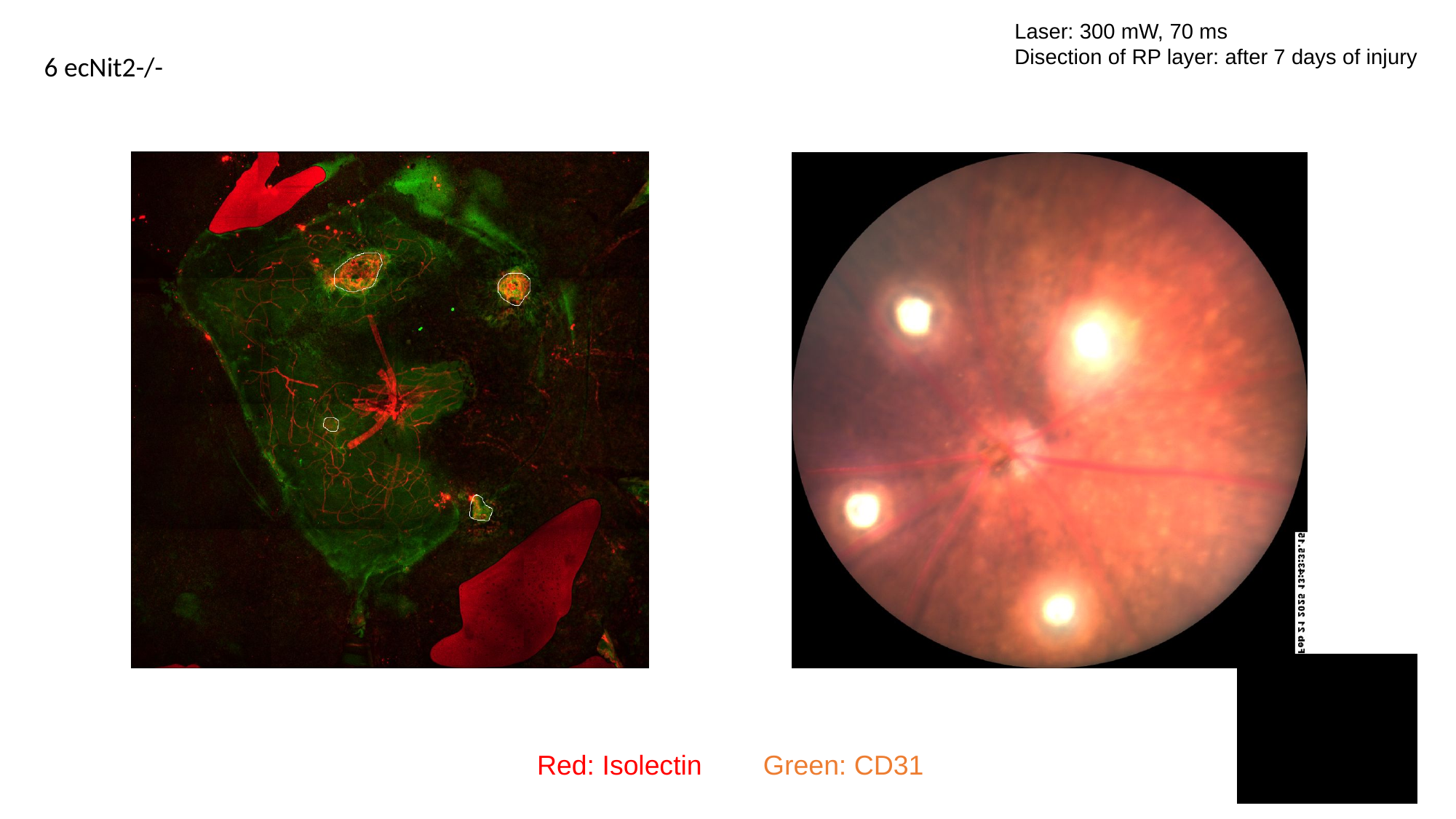

Laser: 300 mW, 70 ms
Disection of RP layer: after 7 days of injury
6 ecNit2-/-
Red: Isolectin Green: CD31

## Slide 8
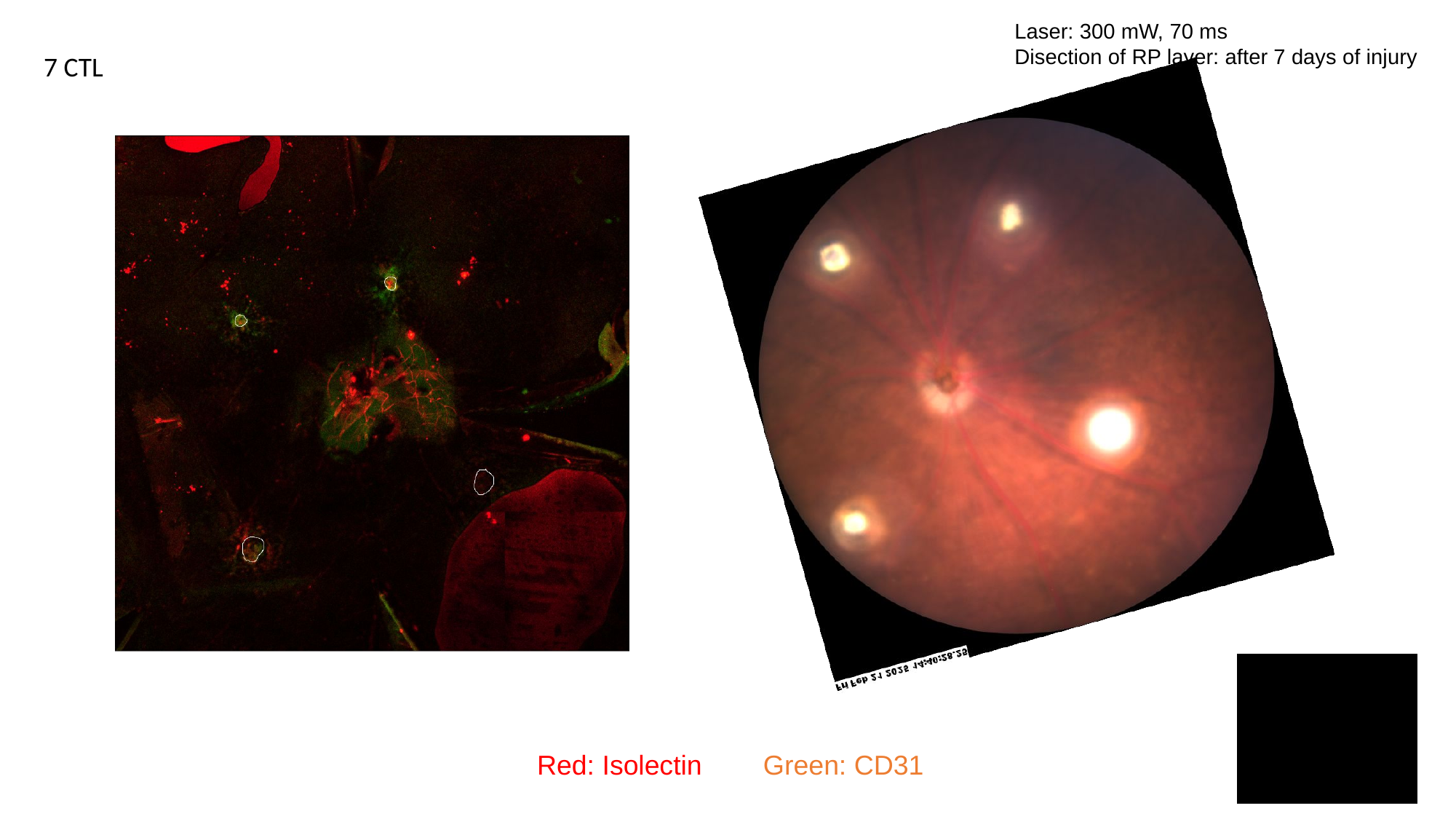

Laser: 300 mW, 70 ms
Disection of RP layer: after 7 days of injury
7 CTL
Red: Isolectin Green: CD31

## Slide 9
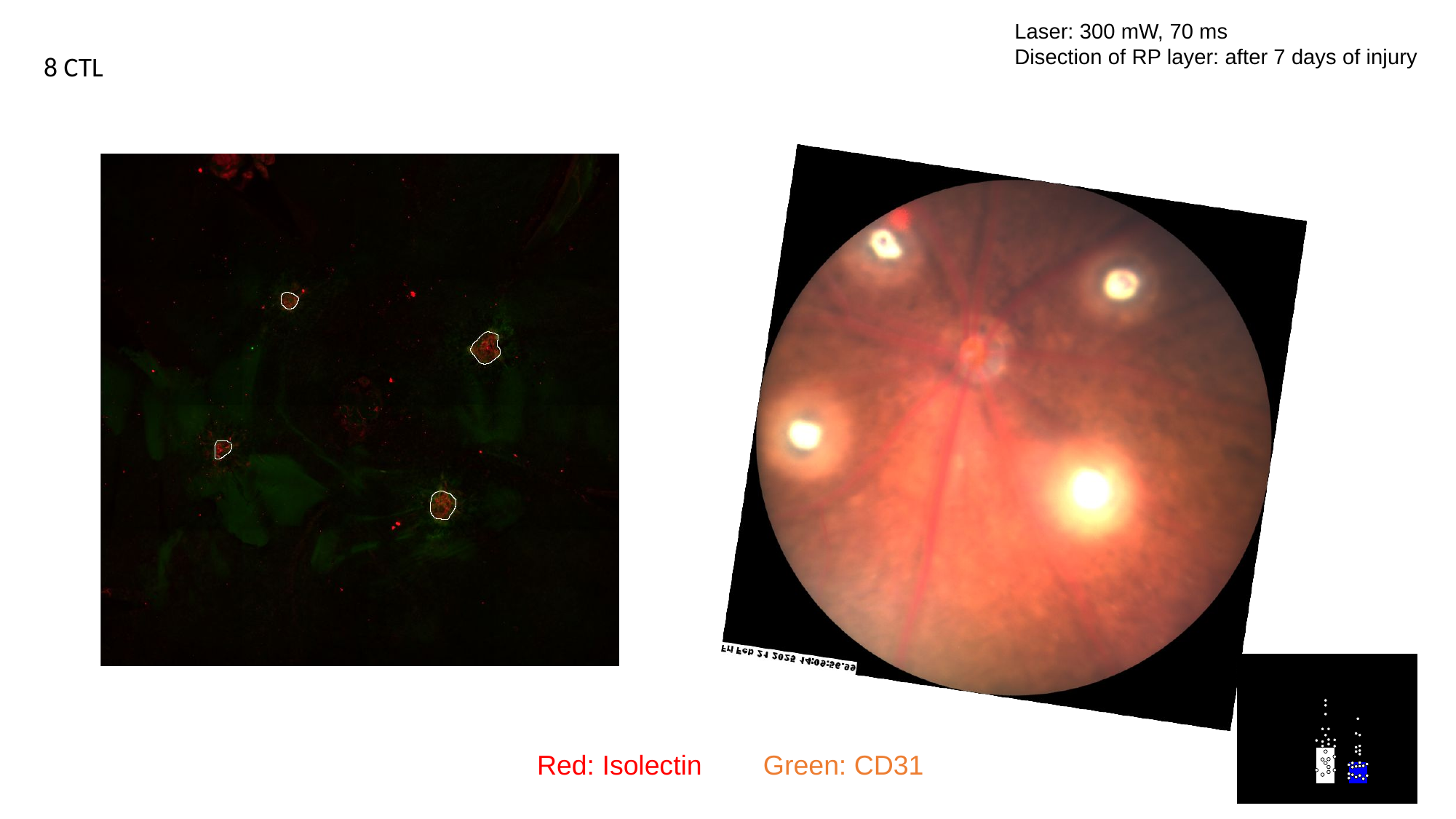

Laser: 300 mW, 70 ms
Disection of RP layer: after 7 days of injury
8 CTL
Red: Isolectin Green: CD31

## Slide 10
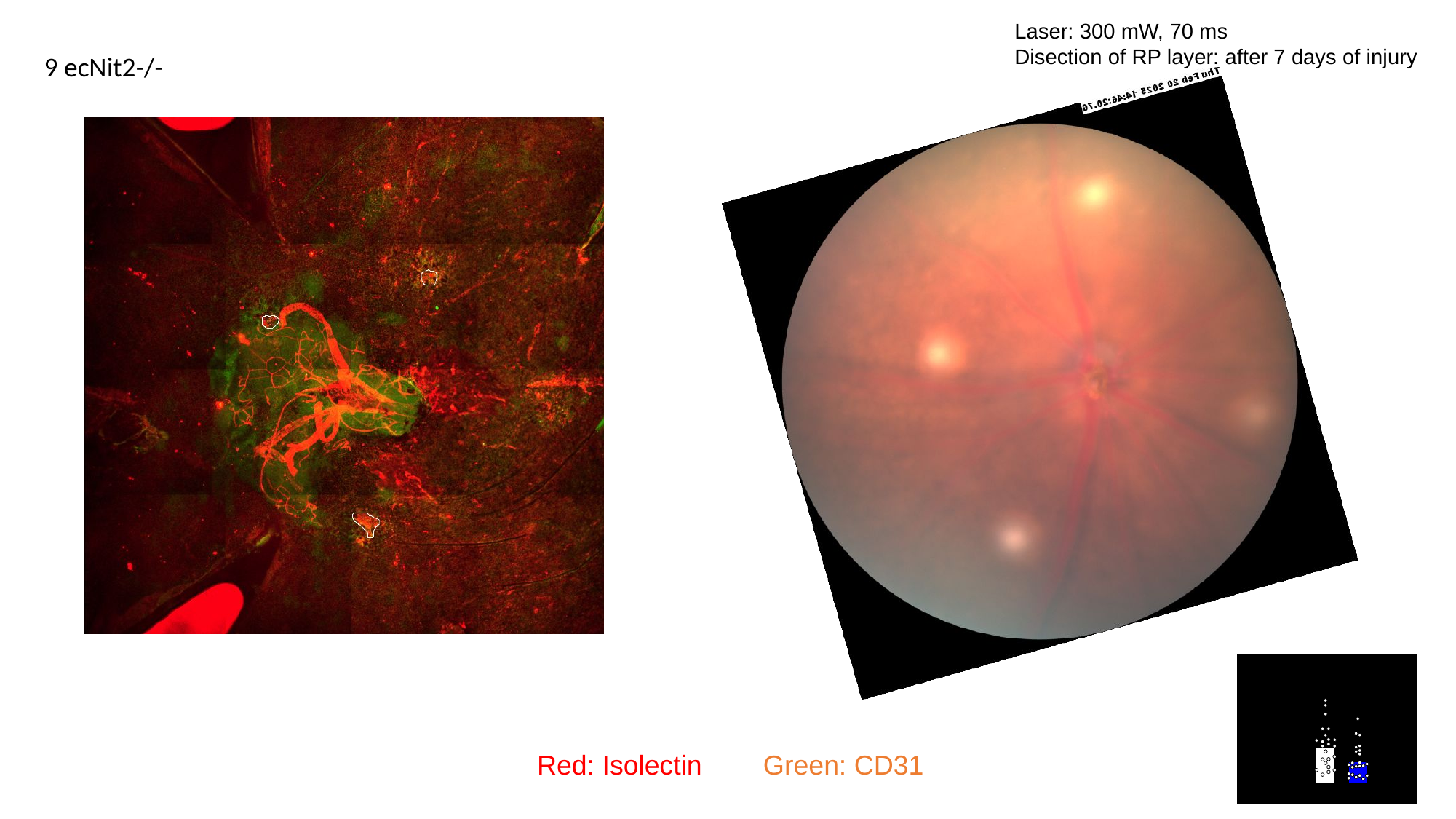

Laser: 300 mW, 70 ms
Disection of RP layer: after 7 days of injury
9 ecNit2-/-
Red: Isolectin Green: CD31

## Slide 11
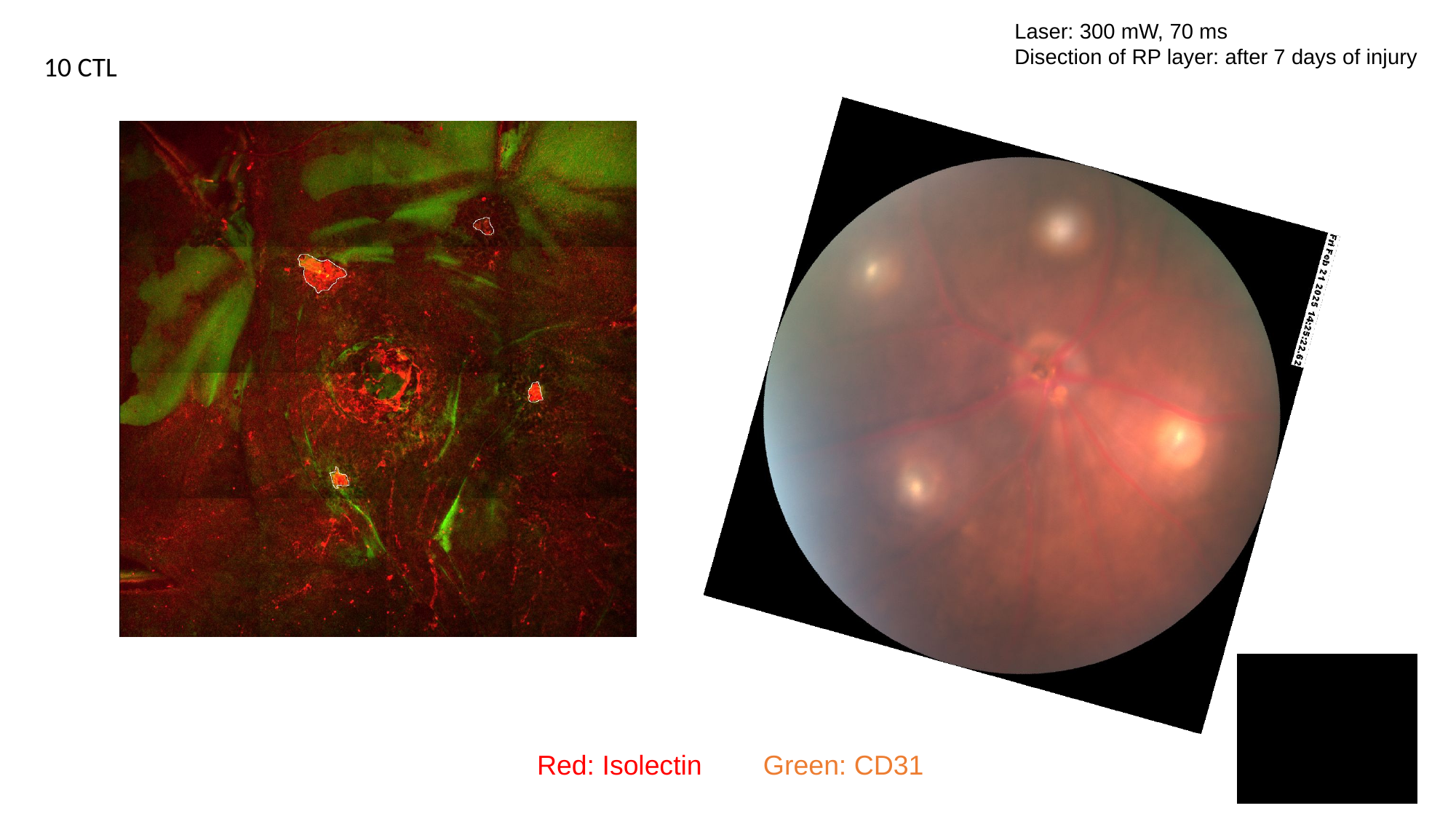

Laser: 300 mW, 70 ms
Disection of RP layer: after 7 days of injury
10 CTL
Red: Isolectin Green: CD31

## Slide 12
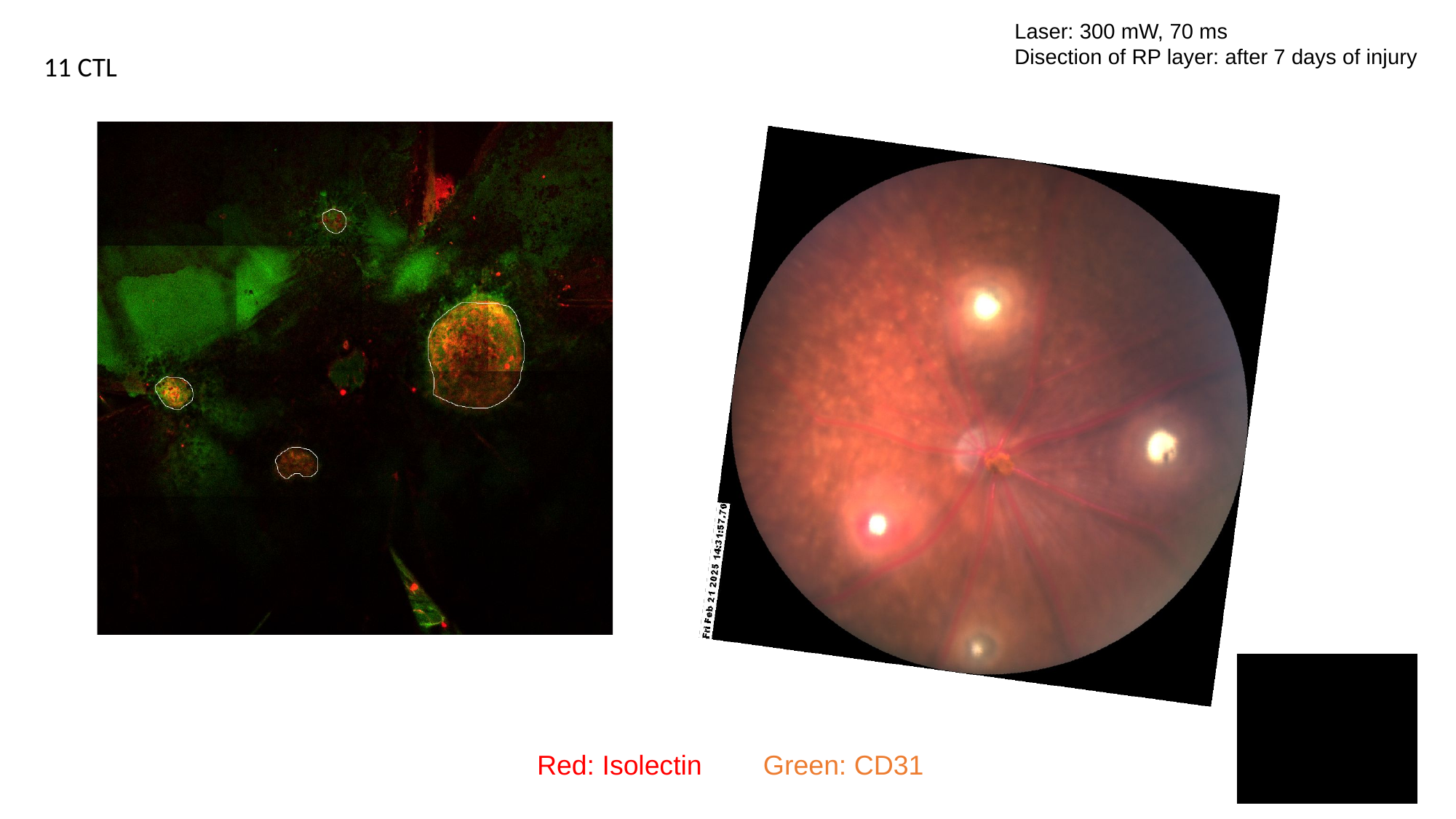

Laser: 300 mW, 70 ms
Disection of RP layer: after 7 days of injury
11 CTL
Red: Isolectin Green: CD31

## Slide 13
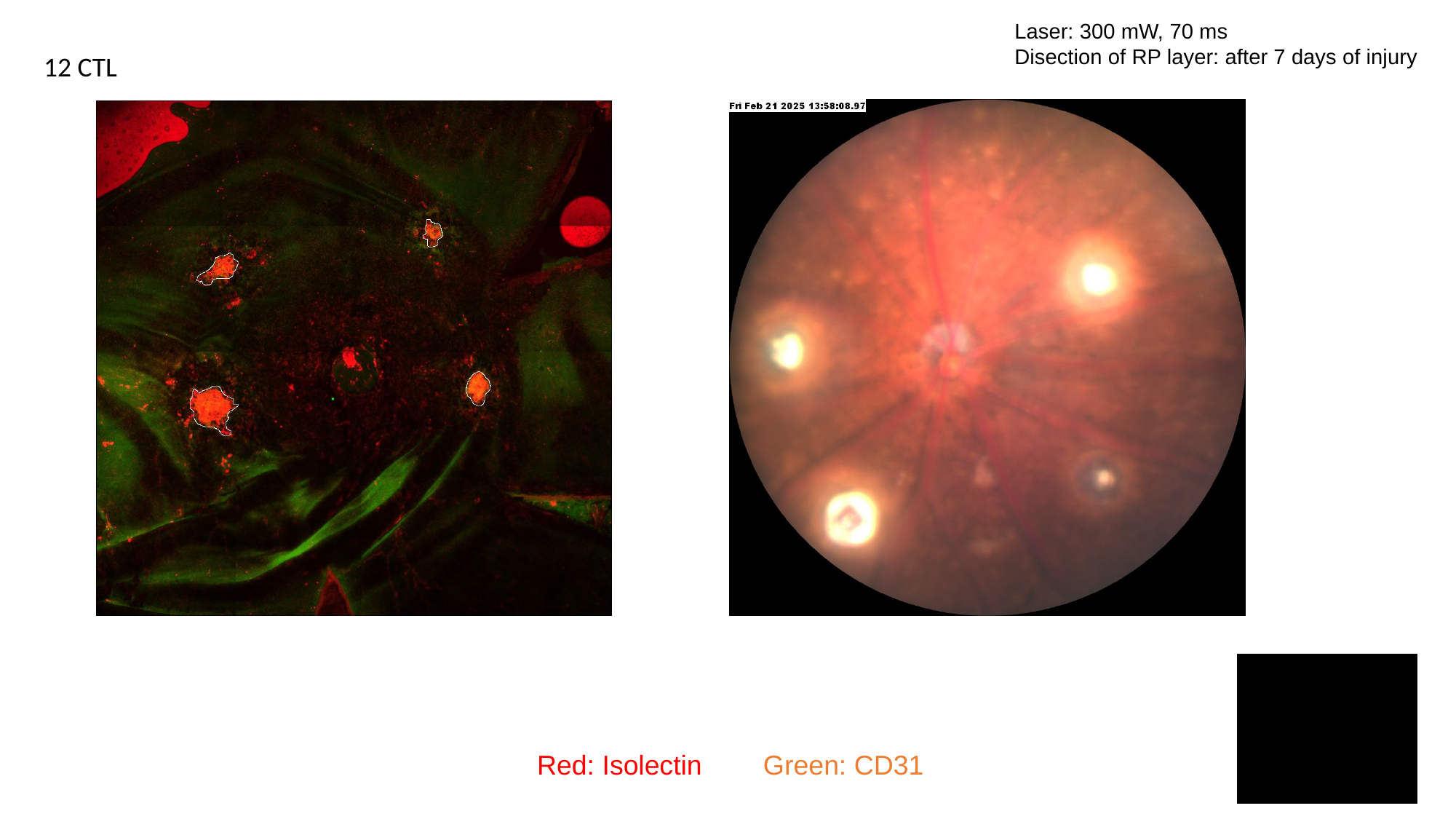

Laser: 300 mW, 70 ms
Disection of RP layer: after 7 days of injury
12 CTL
Red: Isolectin Green: CD31
