# Supplementary material for: The transaminase-ω-amidase pathway senses oxidative stress to control glutamine metabolism and α-ketoglutarate levels in endothelial cells
Source: EMBO J. 2025 Dec 17;45(3):820–55. doi: 10.1038/s44318-025-00642-7 (PMC12864753; doi:10.1038/s44318-025-00642-7)

Quality Control Slides

NTC HUVEC
EBM + 6% FCS
Basal

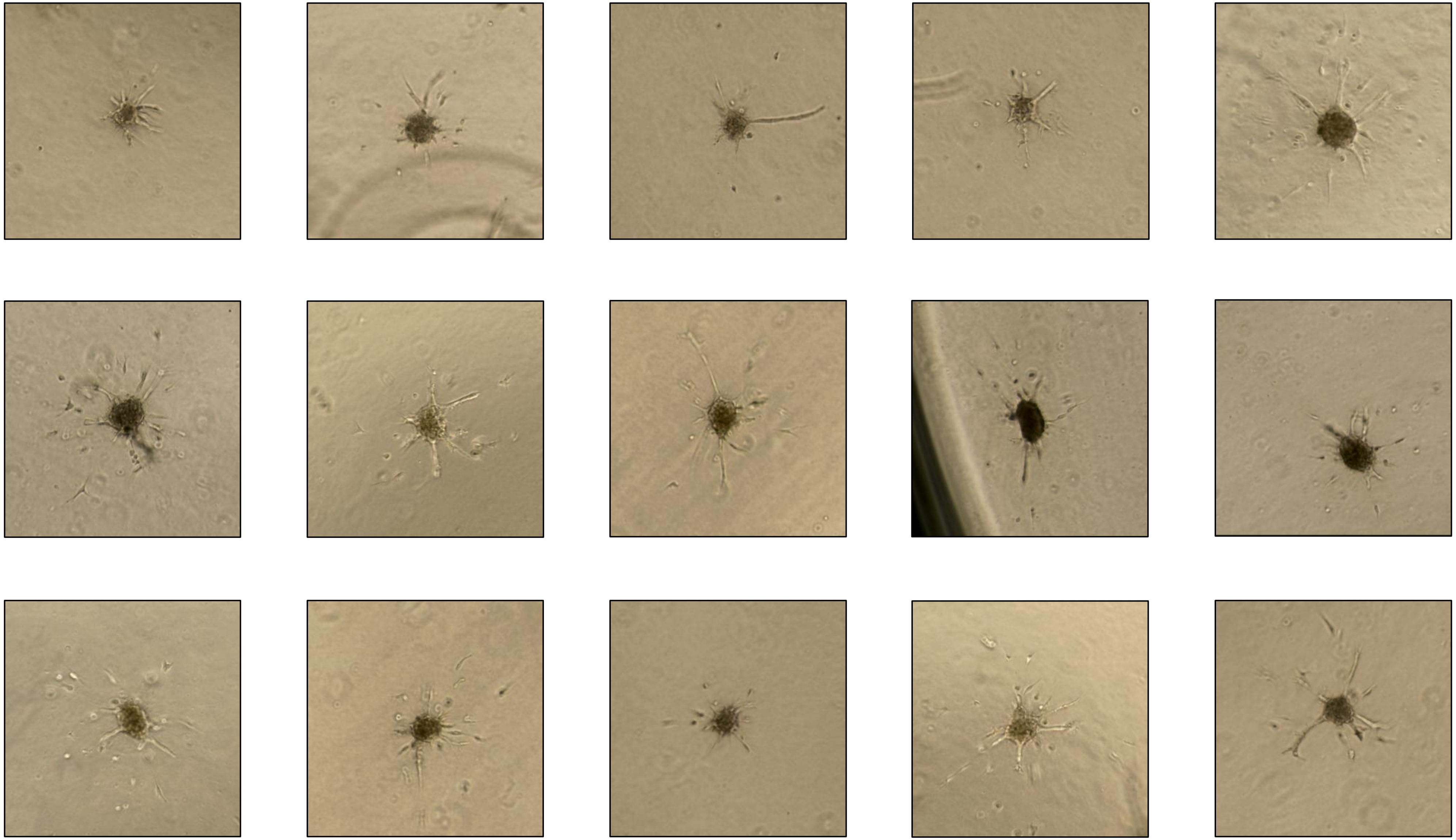

NTC HUVEC
EBM + 6% FCS
VEGF-A

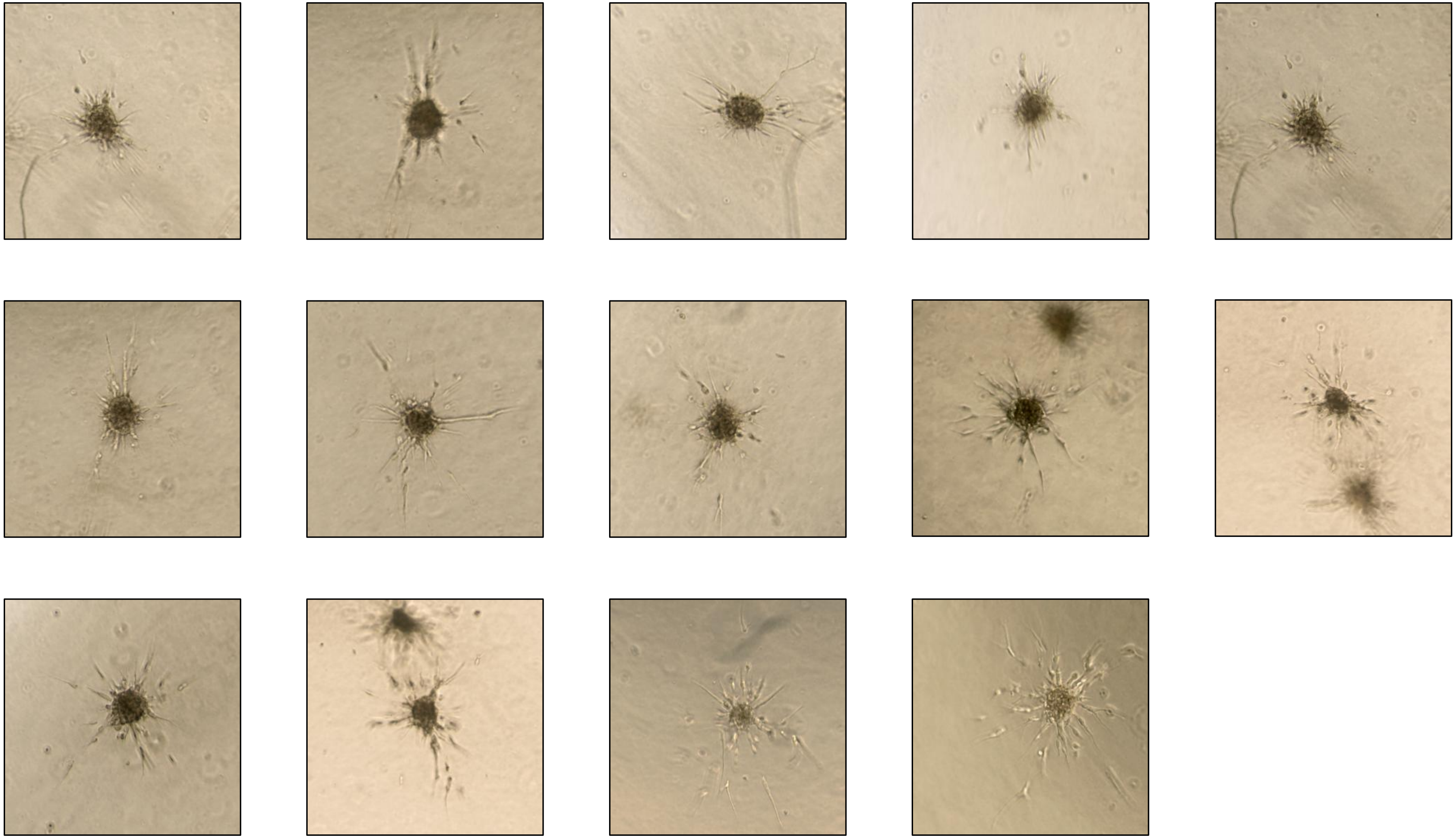

NIT2$^{-/-}$ HUVEC
EBM + 6% FCS
Basal

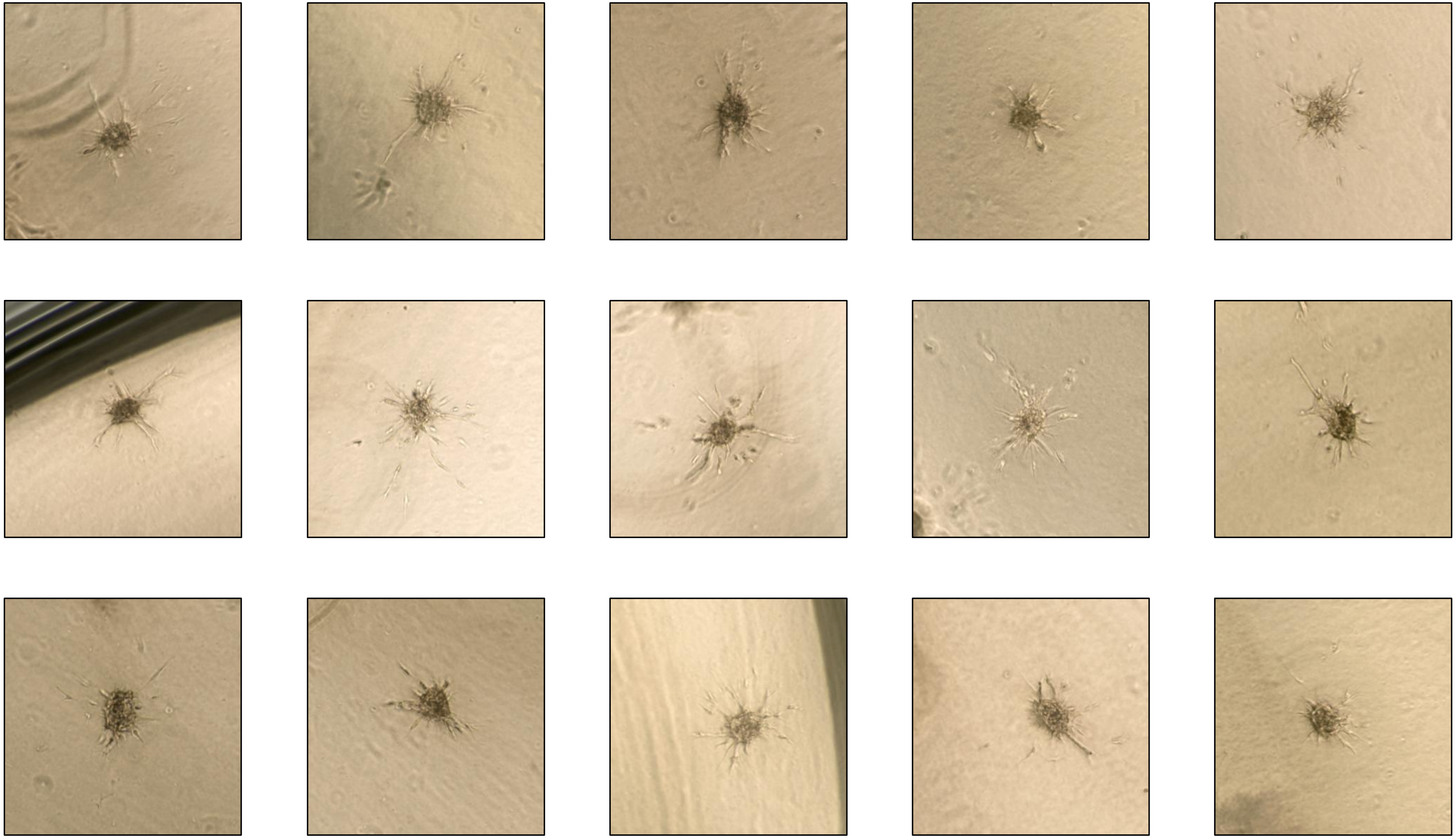

NIT2$^{-/-}$ HUVEC
EBM + 6% FCS
VEGF-A

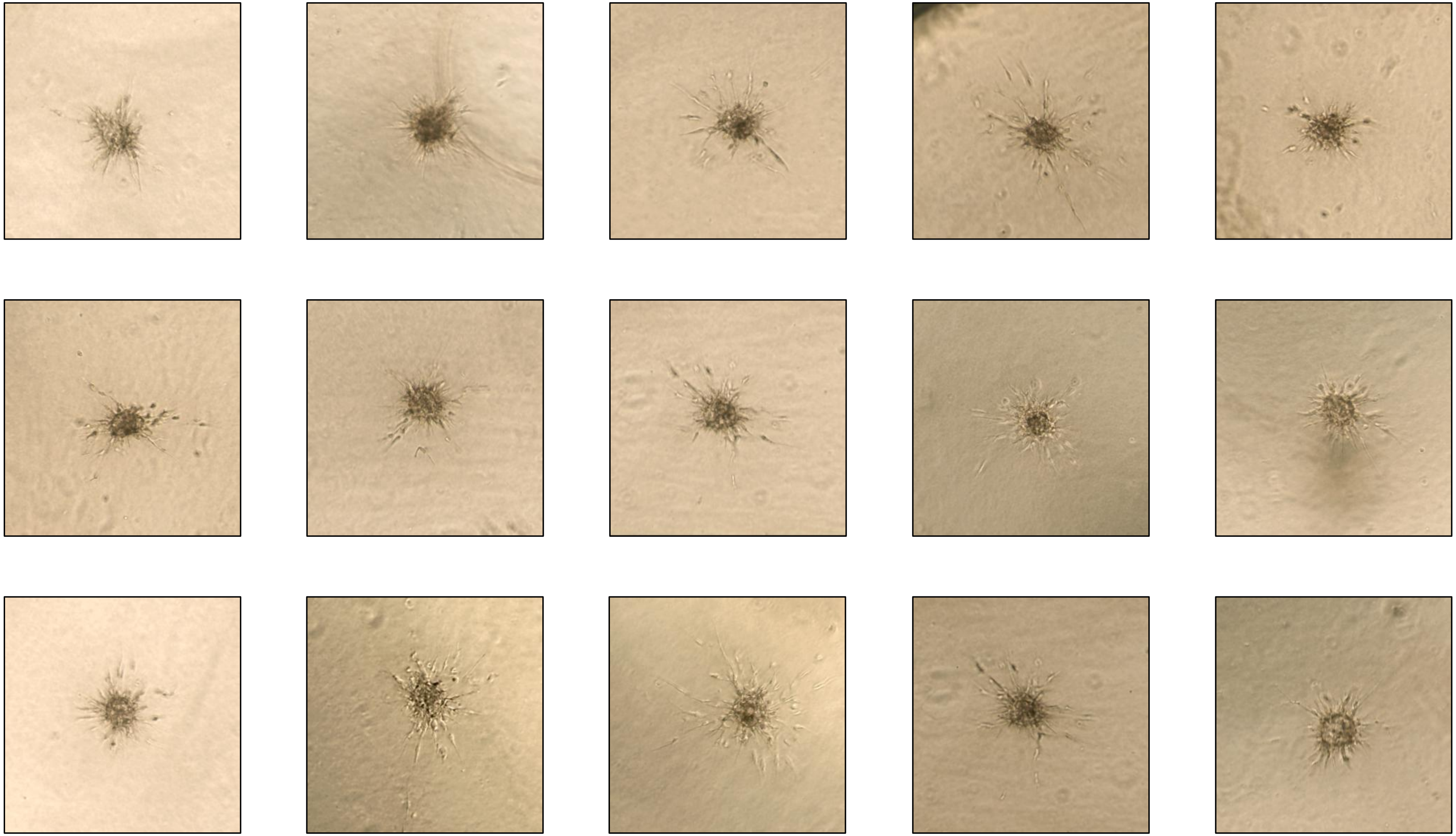

GLS1$^{-/-}$ HUVEC
EBM + 6% FCS
Basal

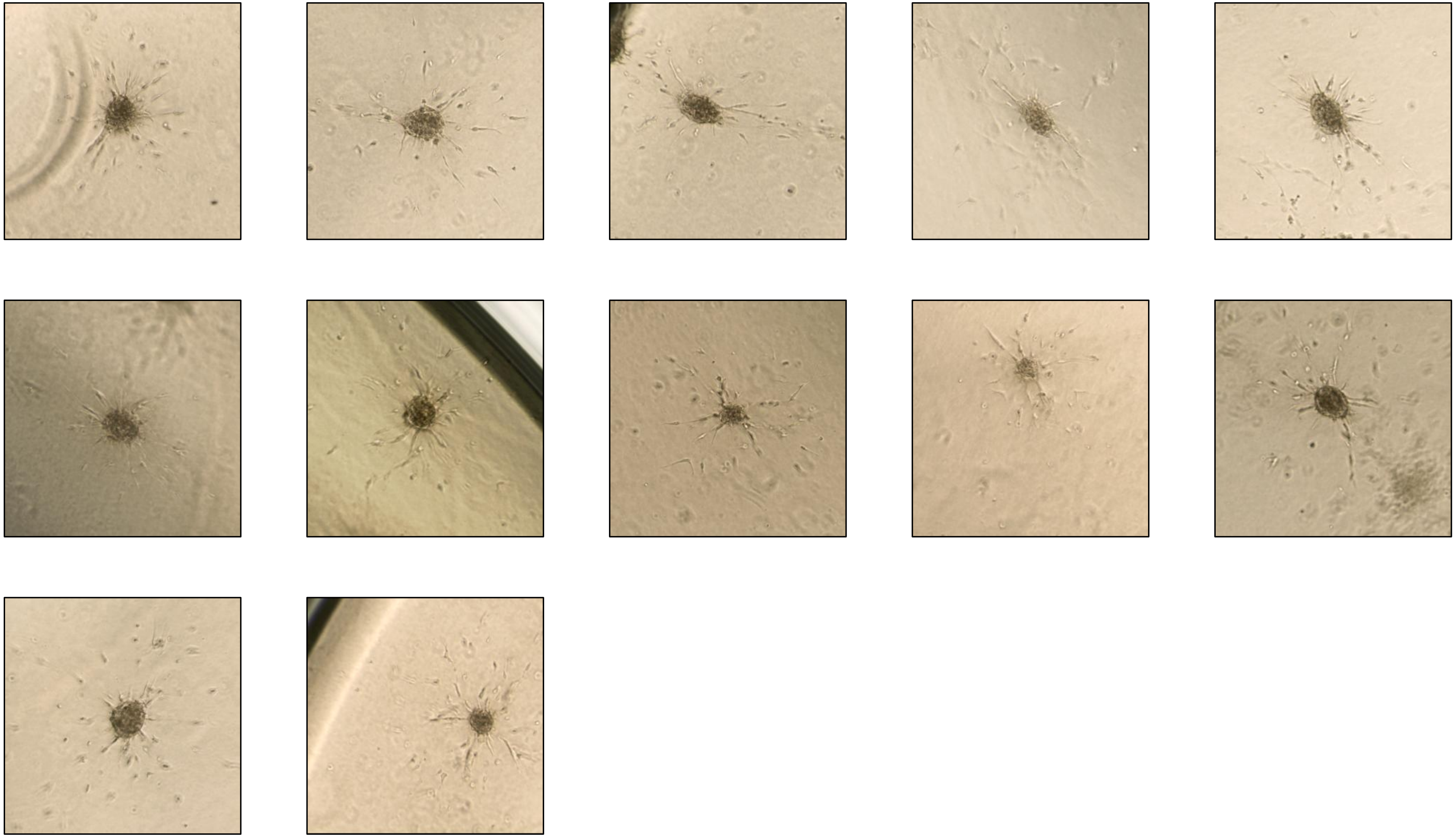

$GLS1^{-/-}$ HUVEC
EBM + 6% FCS
VEGF-A

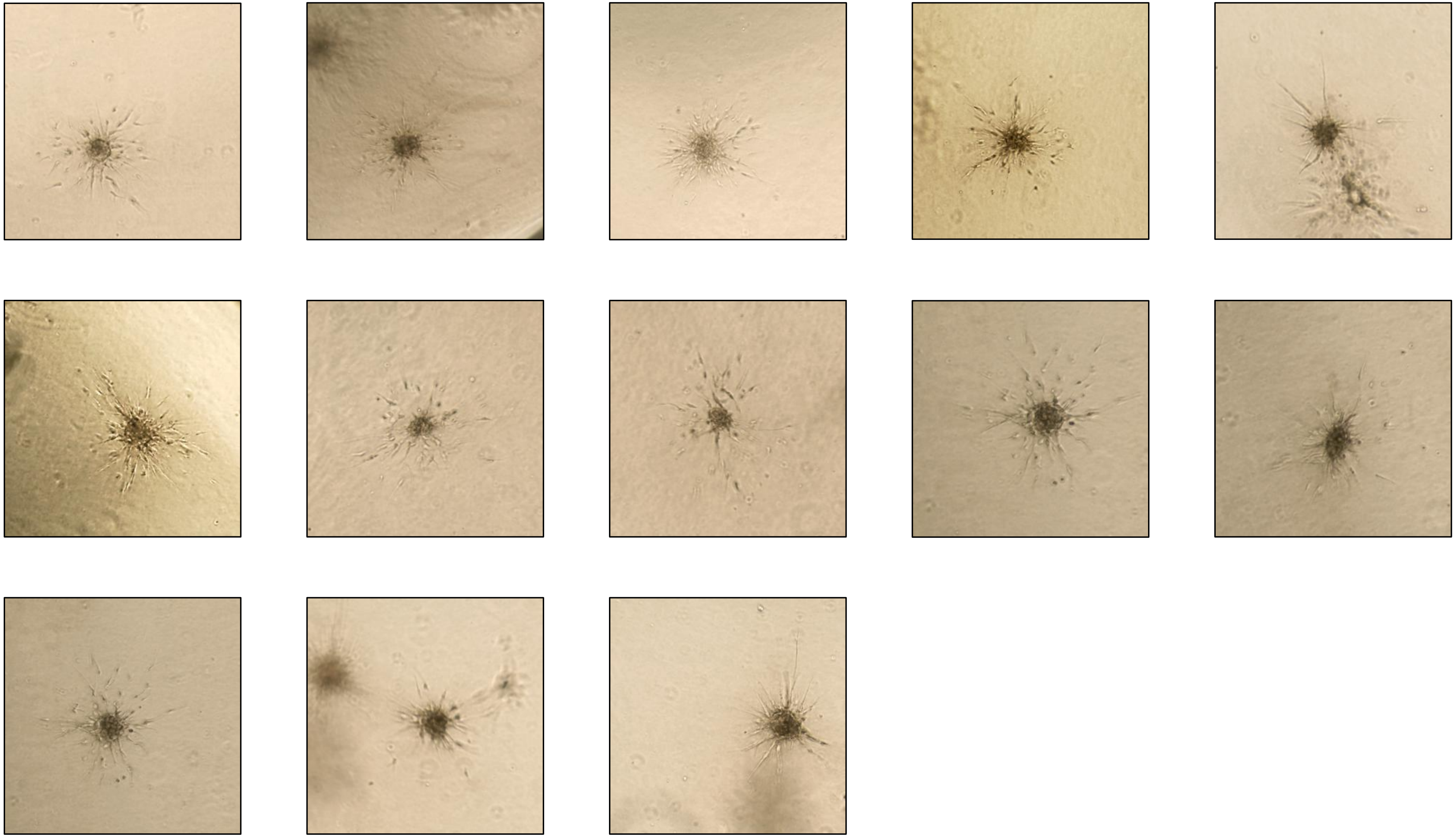

NIT2/GLS1$^{-/-}$ HUVEC
EBM + 6% FCS
Basal

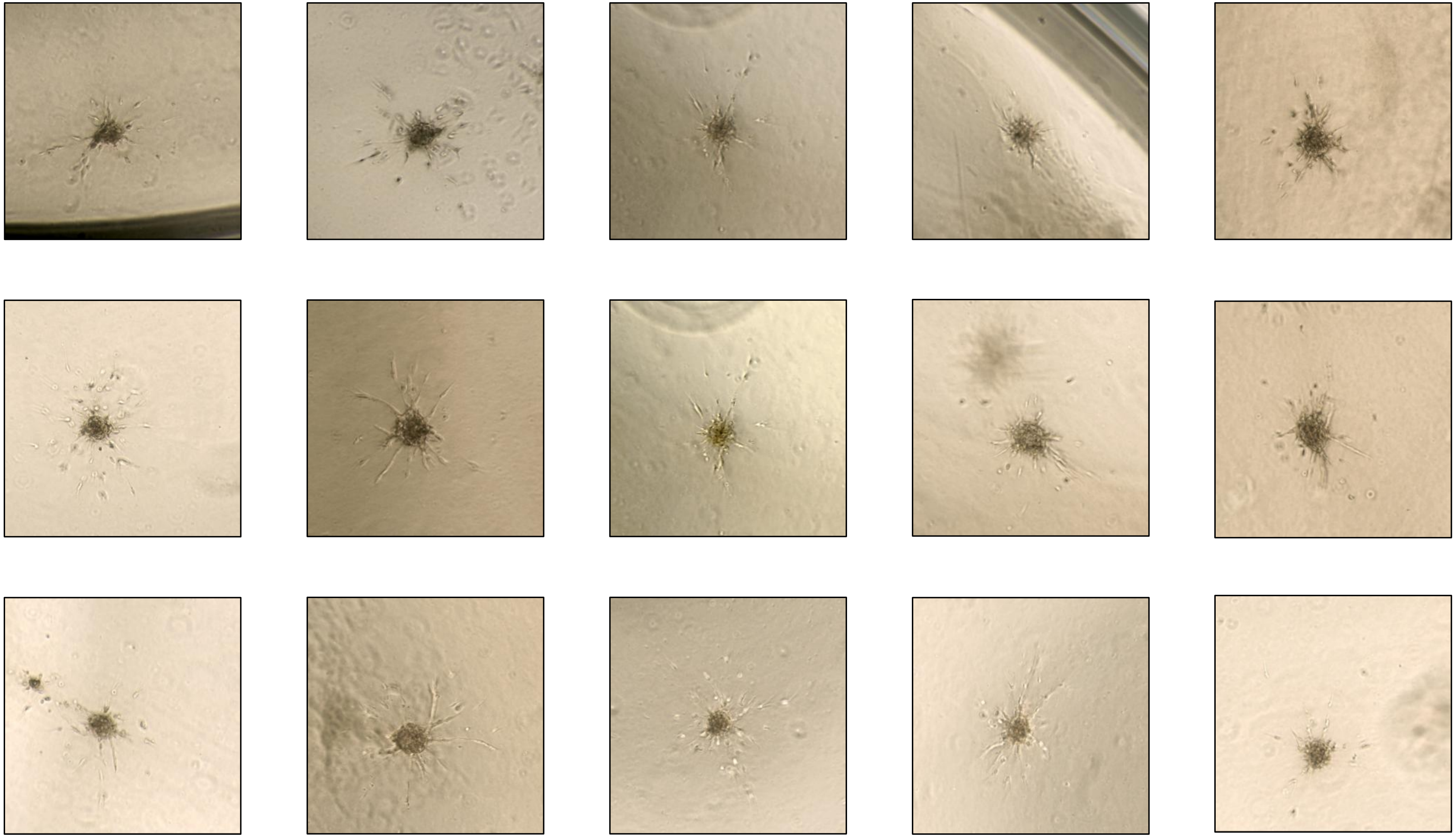

NIT2/GLS1$^{-/-}$ HUVEC
EBM + 6% FCS
VEGF-A

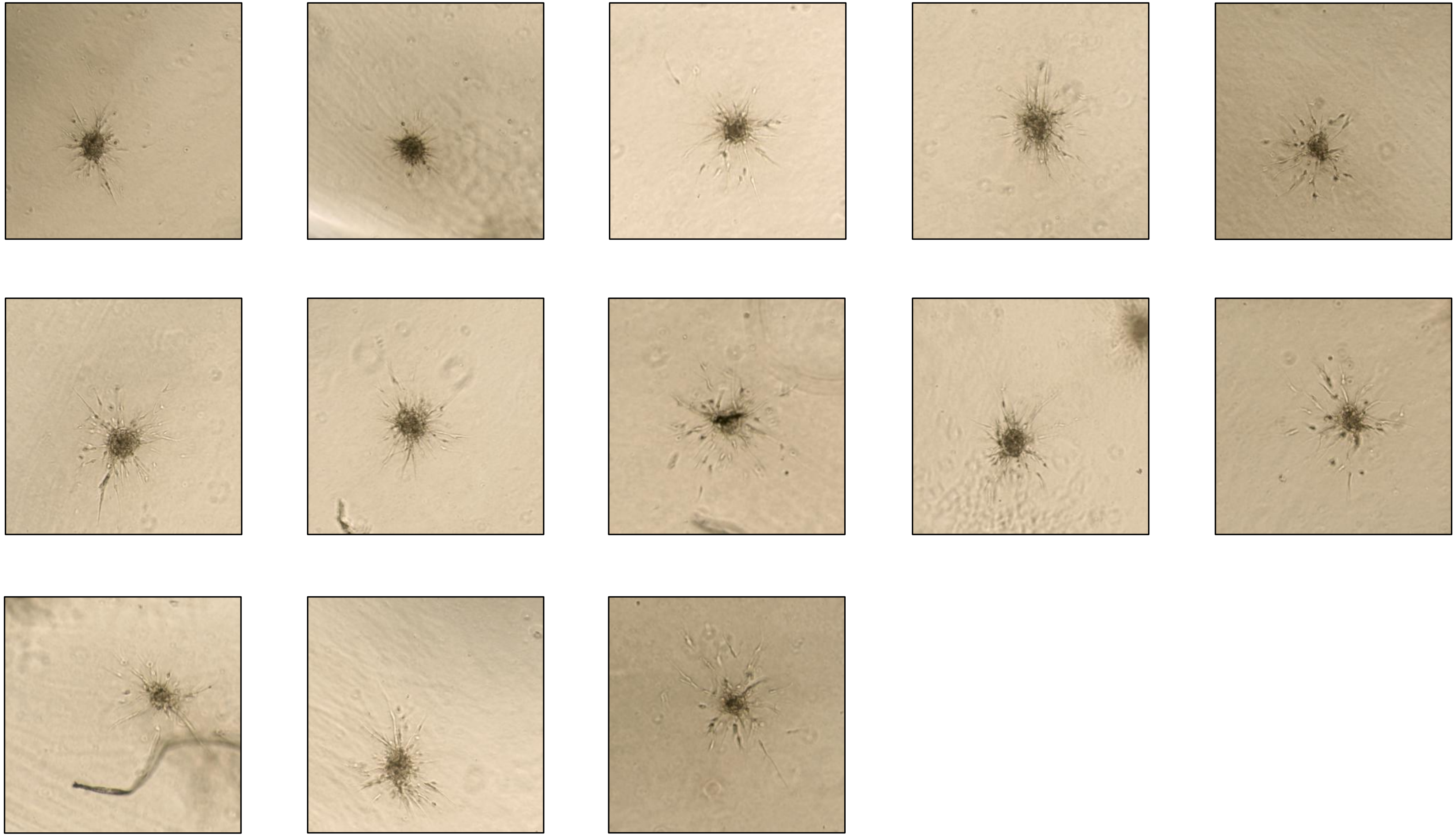

Supplement: Supplementary file 12 — Source data Fig. 5 [file 44318_2025_642_MOESM12_ESM.zip › Figure 5/Fig. 5A.pdf]
