# Supplementary material for: The transaminase-ω-amidase pathway senses oxidative stress to control glutamine metabolism and α-ketoglutarate levels in endothelial cells
Source: EMBO J. 2025 Dec 17;45(3):820–55. doi: 10.1038/s44318-025-00642-7 (PMC12864753; doi:10.1038/s44318-025-00642-7)
Supplement: Supplementary file 13 — Source data Fig. 6 [file 44318_2025_642_MOESM13_ESM.zip › Figure 6/Fig. 6B_J.pptx]

## Slide 1
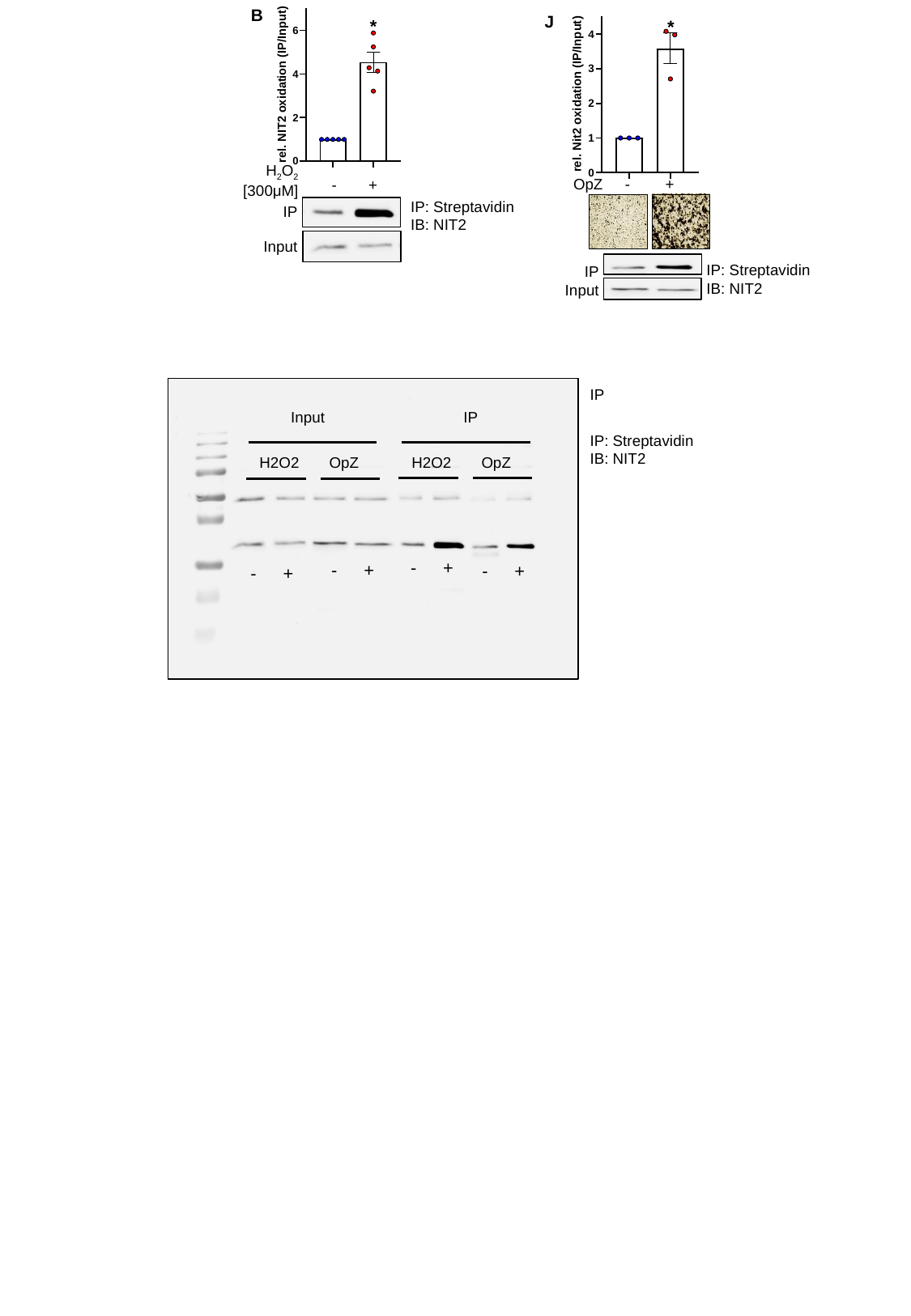

B
J
H2O2
[300μM]
OpZ
-
+
-
+
IP: Streptavidin
IB: NIT2
IP
Input
IP: Streptavidin
IB: NIT2
IP
Input
IP
IP
Input
IP: Streptavidin
IB: NIT2
H2O2
OpZ
H2O2
OpZ
-
+
-
+
-
+
-
+

## Slide 2
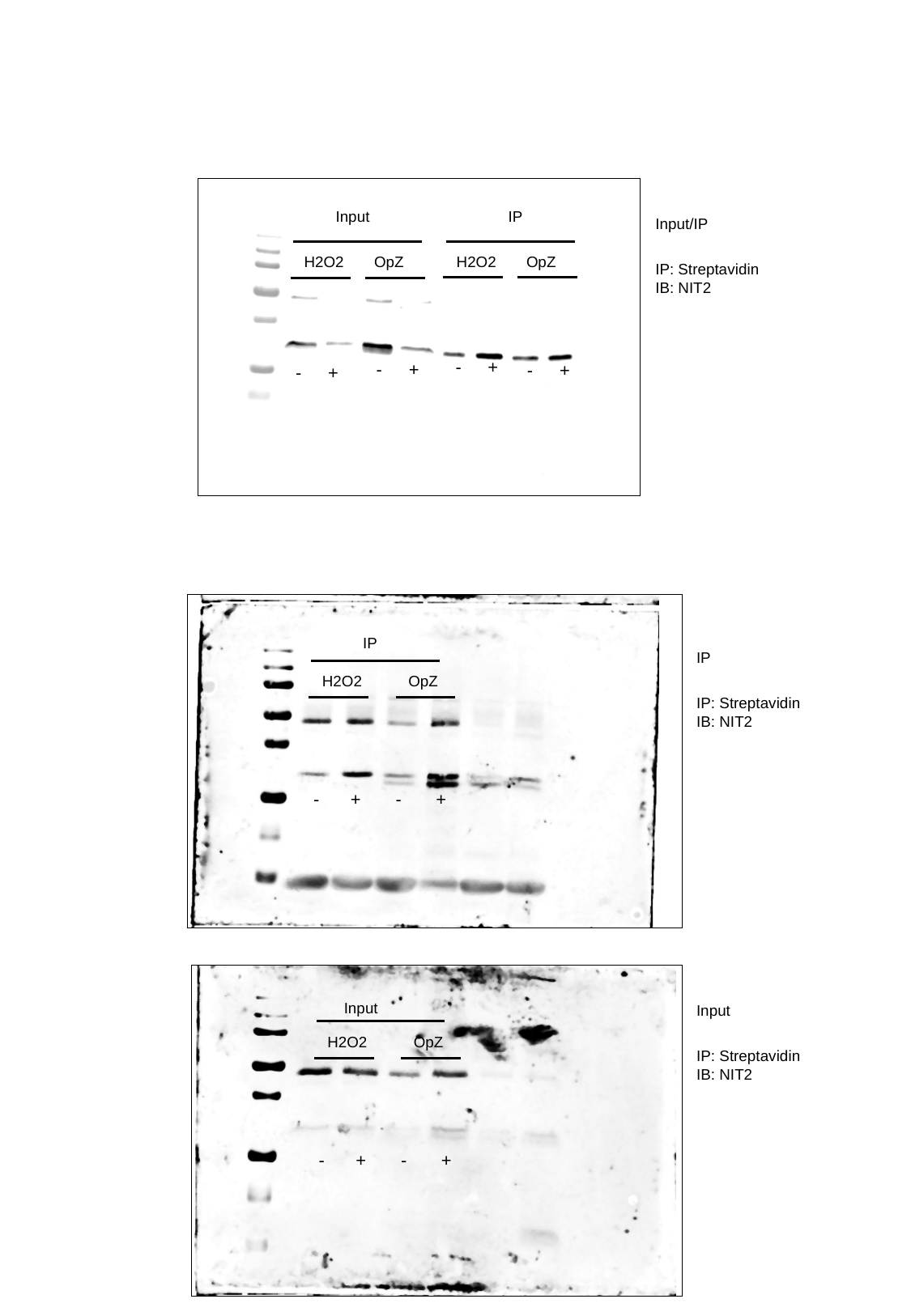

IP
Input
Input/IP
H2O2
OpZ
H2O2
OpZ
IP: Streptavidin
IB: NIT2
-
+
-
+
-
+
-
+
IP
IP
H2O2
OpZ
IP: Streptavidin
IB: NIT2
-
+
-
+
Input
Input
H2O2
OpZ
IP: Streptavidin
IB: NIT2
-
+
-
+
