# Supplementary material for: The transaminase-ω-amidase pathway senses oxidative stress to control glutamine metabolism and α-ketoglutarate levels in endothelial cells
Source: EMBO J. 2025 Dec 17;45(3):820–55. doi: 10.1038/s44318-025-00642-7 (PMC12864753; doi:10.1038/s44318-025-00642-7)
Supplement: Supplementary file 19 — Expanded View Figures [file 44318_2025_642_MOESM19_ESM.pdf]

Expanded View Figures

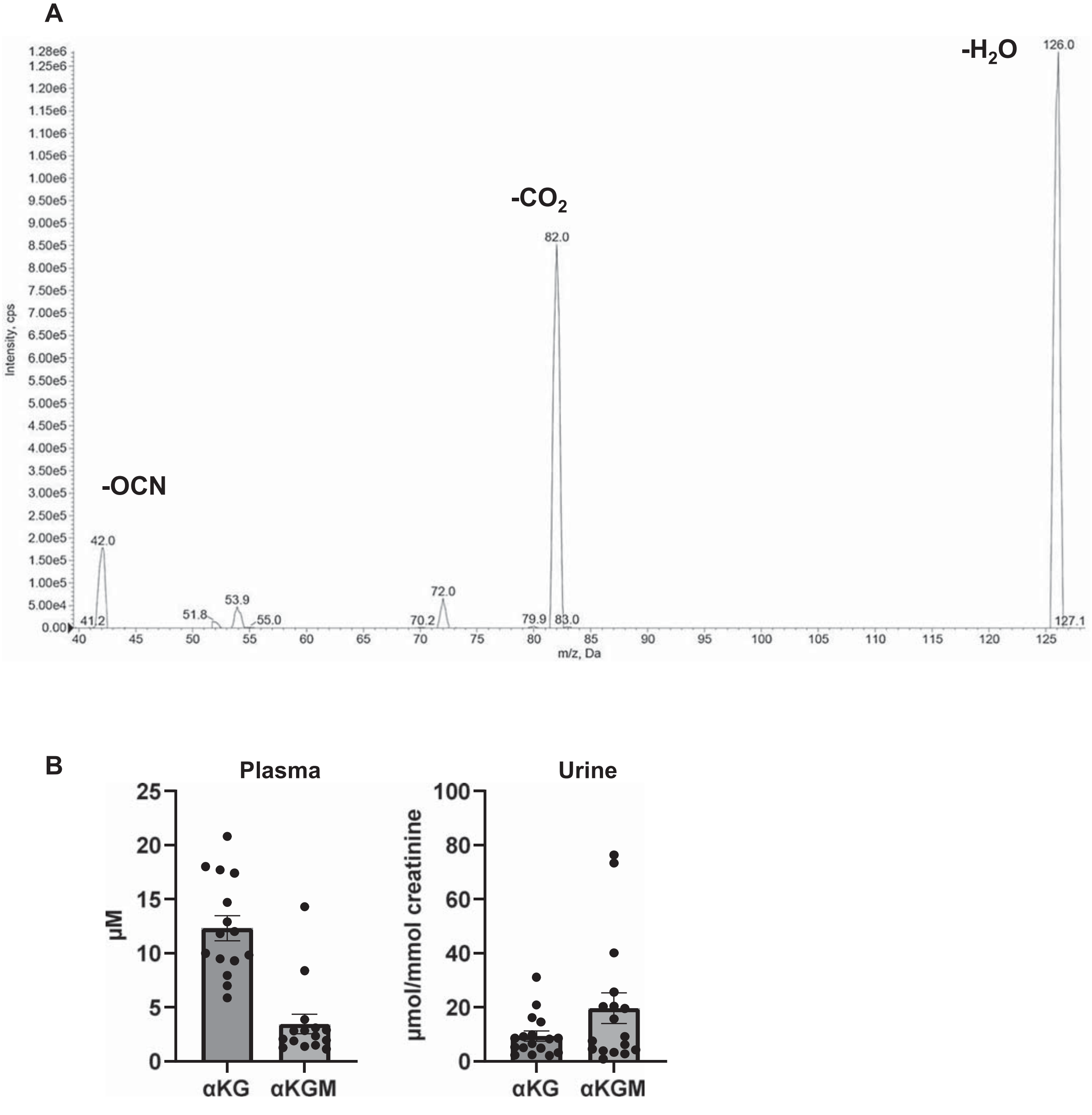

Figure EV1. Targeted LC-MS/MS of αKGM.

(**A**) The mass spectrum of αKGM in its lactam form as detected in negative ionization mode. The spectrum shows three major fragmentation peaks at 126.0 Da (M-1-H2O; -18 Da), 82.0 Da (M-1-CO2-H2O; -62 Da) and 42.0 Da (a CNO- fragment). (**B**) αKG and αKGM concentrations (using calibration curves of known concentrations of each compound) in plasma and urine (normalized to creatinine) of healthy individuals. Source data are available online for this figure.

© The Author(s)

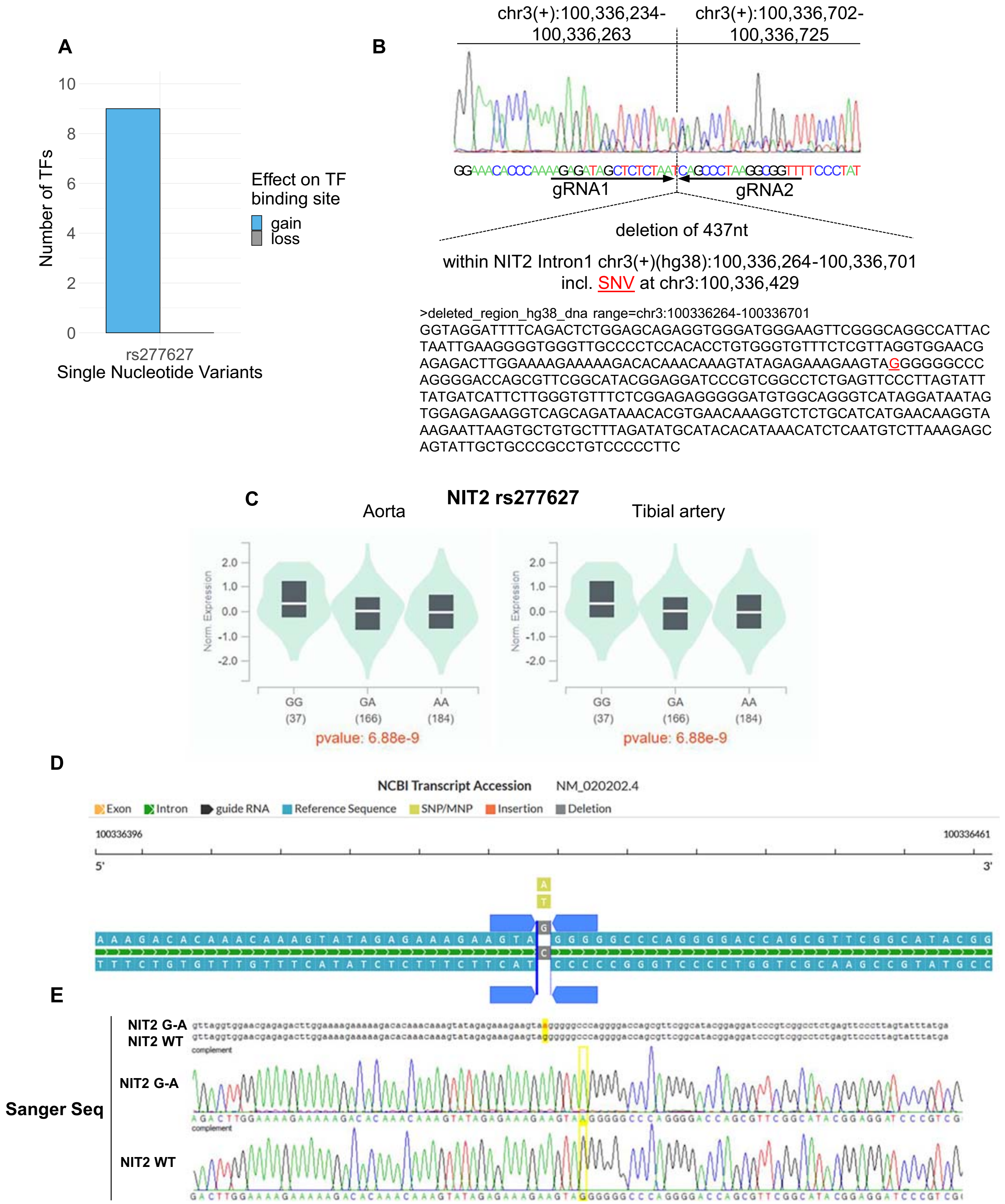
A
10
8
6
4
2
0
Number of TFs
rs277627
Single Nucleotide Variants
Effect on TF binding site
gain
loss
B
chr3(+):100,336,234-100,336,263
chr3(+):100,336,702-100,336,725
gRNA1
gRNA2
deletion of 437nt
within NIT2 Intron1 chr3(+)(hg38):100,336,264-100,336,701
incl. SNV at chr3:100,336,429
>deleted_region_hg38_dna range=chr3:100336264-100336701
GGTAGGATTTTCAGACTCTGGAGCAGAGGTGGGATGGGAAGTTCGGGCAGGCCATTAC
TAATTGAAGGGGTGGGTTGCCCCTCCACACCTGTGGGTGTTTCTCGTTAGGTGGAACG
AGAGACTTGGAAAAGAAAAAGACACAAACAAAGTATAGAGAAAGAAGTAGGGGGGCCC
AGGGGACCAGCGTTCGGCATACGGAGGATCCCGTCGGCCTCTGAGTTCCCTTAGTATT
TATGATCATTCTTGGGTGTTTCTCGGAGAGGGGGATGTGGCAGGGTCATAGGATAATAG
TGGAGAGAAGGTCAGCAGATAAACACGTGAACAAAGGTCTCTGCATCATGAACAAGGTA
AAGAATTAAGTGCTGTGCTTTAGATATGCATACACATAAACATCTCAATGTCTTAAAGAGC
AGTATTGCTGCCCGCCTGTCCCCCTTC
C
NIT2 rs277627
Aorta
Tibial artery
Norm. Expression
GG (37)
GA (166)
AA (184)
pvalue: 6.88e-9
D
NCBI Transcript Accession NM_020202.4
Exon
Intron
guide RNA
Reference Sequence
SNP/MNP
Insertion
Deletion
100336396
100336461
5'
3'
E
NIT2 G-A
NIT2 WT
Sanger Seq

Figure EV2. Regulation of *NIT2* expression by the SNV rs277627.

(**A**) rs277627 is located in a regulatory element (REM, analyzed with EpiRegioDB) with binding sites for the transcription factors ZNF320 VEZF1 KLF16 GLIS2 MAZ ZNF740 ZNF148 ZNF467 GLIS3 that are expressed in HUVEC and have a gain of function and likely repress transcription. (**B**) A two gRNA approach was employed to delete a 437 bp region where the SNV rs277627 (chr3:100336428–100336429) in NIT2 (intron 1) is located. gRNA1 targets the chr3:100,336,234-100,336,263 region whereas gRNA2 the chr3:100,336,702-100,336,725. A successful deletion was obtained between 100,336,264-100,336,701 as shown by Sanger sequencing. The SNV G is labeled in red. (**C**) Data from GTex for the expression of wild-type (GG) rs277627 and its variants GA and AA on NIT2 expression in human aorta and tibial artery. (**D**) Strategy of the CRISPR/Cas9-mediated mutation to insert the NIT2 intron containing relevant SNP rs277627. Genomic locus of the human NIT2 (NM_020202.4) intron and mutation of G (WT) to an A (rs277627). (**E**) Sanger Sequencing of genomic DNA after CRISPR/Cas9-mediated generation of the rs277627.

 © The Author(s)

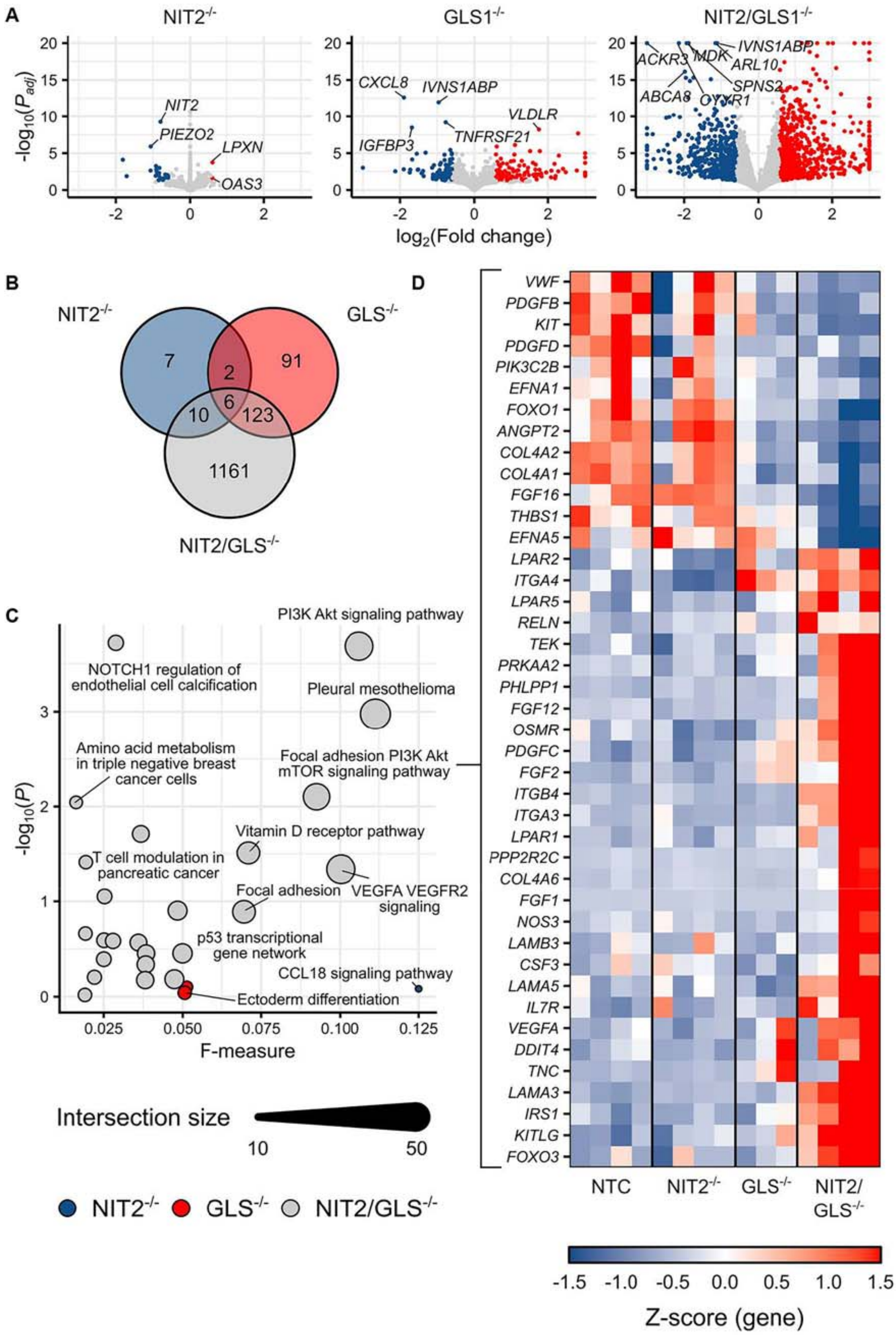
A
NIT2-/-
GLS1-/-
NIT2/GLS1-/-
-log10(Padj)
NIT2
PIEZO2
LPXN
OAS3
CXCL8
IVNS1ABP
IGFBP3
TNFRSF21
VLDLR
ACKR3
MDK
IVNS1ABP
ARL10
SPNS2
ABCA8
OXYR1
log2(Fold change)
B
NIT2-/-
GLS-/-
7
2
91
6
10
123
1161
NIT2/GLS-/-
C
PI3K Akt signaling pathway
NOTCH1 regulation of endothelial cell calcification
Pleural mesothelioma
Amino acid metabolism in triple negative breast cancer cells
Focal adhesion PI3K Akt mTOR signaling pathway
Vitamin D receptor pathway
T cell modulation in pancreatic cancer
Focal adhesion
VEGFA VEGFR2 signaling
p53 transcriptional gene network
CCL18 signaling pathway
Ectoderm differentiation
-log10(P)
F-measure
Intersection size
10
50
NIT2-/- GLS-/- NIT2/GLS-/-
D
VWF
PDGFB
KIT
PDGFD
PIK3C2B
EFNA1
FOXO1
ANGPT2
COL4A2
COL4A1
FGF16
THBS1
EFNA5
LPAR2
ITGA4
LPAR5
RELN
TEK
PRKAA2
PHLPP1
FGF12
OSMR
PDGFC
FGF2
ITGB4
ITGA3
LPAR1
PPP2R2C
COL4A6
FGF1
NOS3
LAMB3
CSF3
LAMA5
IL7R
VEGFA
DDIT4
TNC
LAMA3
IRS1
KITLG
FOXO3
NTC
NIT2-/-
GLS-/-
NIT2/GLS-/-
-1.5 -1.0 -0.5 0.0 0.5 1.0 1.5
Z-score (gene)

Figure EV3. NIT2 and GLS1 sinergistically control gene expression (RNAseq) in HUVEC.

(**A**) Volcano plots for each knockout as indicated. (**B**) Number of differentially expressed genes. (**C**) Annotation pathway. (**D**) Heatmap of top differentially expressed genes in HUVEC NTC, NIT2$^{-/-}$, GLS1$^{-/-}$ and NIT2/GLS1$^{-/-}$.

 © The Author(s)

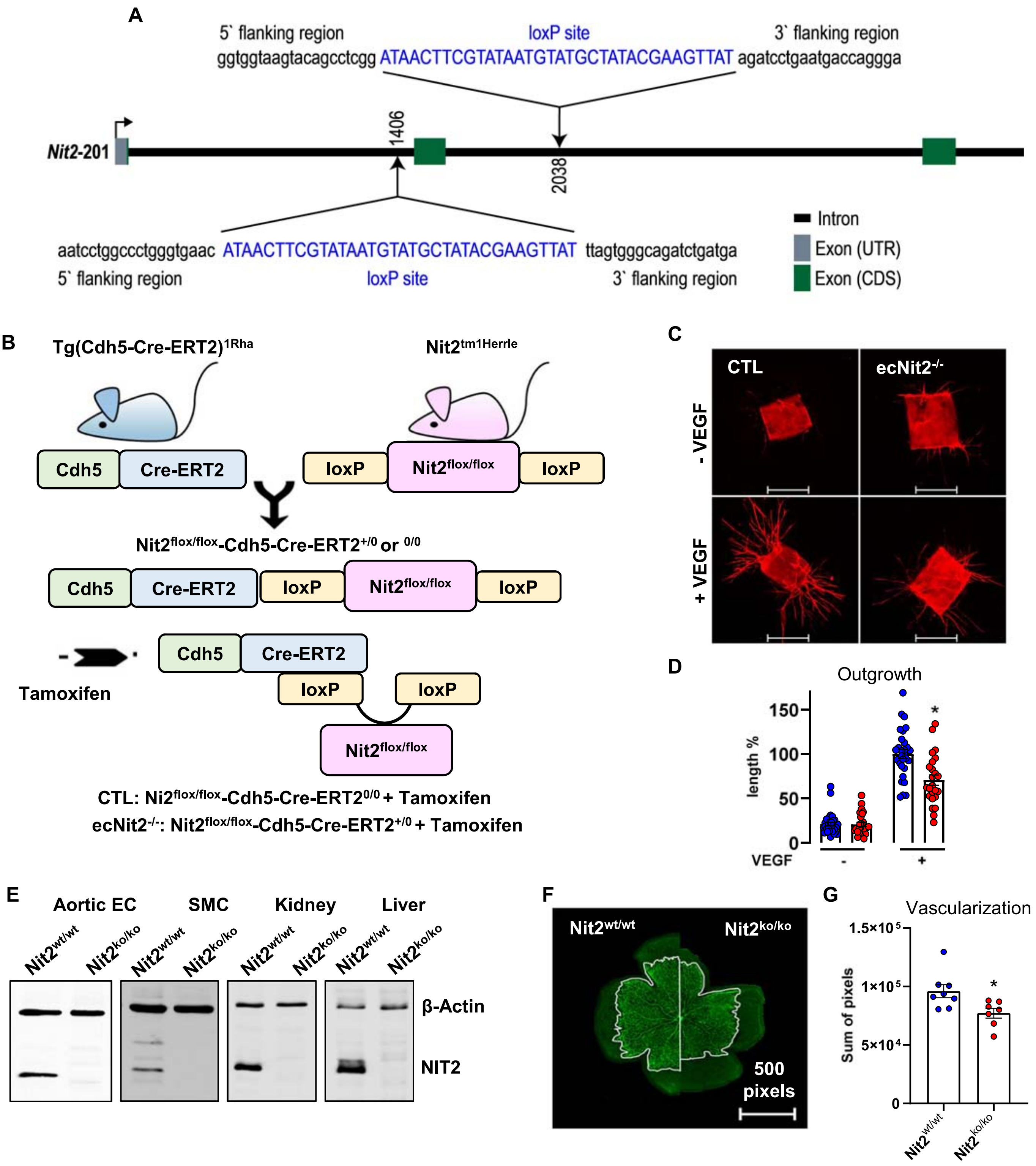
A
5` flanking region
loxP site
3` flanking region
ggtggtaagtacagcctcgg ATAACTTCGTATAATGTATGCTATACGAAGTTAT agatcctgaatgaccaggga
Nit2-201
1406
2038
aatcctggccctgggtgaac ATAACTTCGTATAATGTATGCTATACGAAGTTAT ttagtgggcagatctgatga
5` flanking region
loxP site
3` flanking region
Intron
Exon (UTR)
Exon (CDS)
B
Tg(Cdh5-Cre-ERT2)1Rha
Nit2tm1Herrle
Cdh5
Cre-ERT2
loxP
Nit2flox/flox
loxP
Nit2flox/flox-Cdh5-Cre-ERT2+/0 or 0/0
Tamoxifen
CTL: Ni2flox/flox-Cdh5-Cre-ERT20/0 + Tamoxifen
ecNit2-/-: Nit2flox/flox-Cdh5-Cre-ERT2+/0 + Tamoxifen
C
CTL
ecNit2-/-
- VEGF
+ VEGF
D
Outgrowth
length %
150
100
50
0
VEGF
-
+
*
E
Aortic EC
SMC
Kidney
Liver
Nit2wt/wt
Nit2ko/ko
β-Actin
NIT2
F
Nit2wt/wt
Nit2ko/ko
500 pixels
G
Vascularization
Sum of pixels
1.5×10 5
1×10 5
5×10 4
0
*
Nit2wt/wt
Nit2ko/ko

Figure EV4. Generation of Nit2 knockout mice.

(**A**) LoxP sites flanking exon 2 of the *NIT2* gene were inserted by CRISPR/cas9. (**B**) Nit2$^{flox/flox}$ mice were crossed with Tg(Cdh5-Cre-ERT2)^{1Rha} to generate endothelial-specific, tamoxifen-inducible knockout mice of Nit2. (**C**) Ex vivo endothelial cell outsprouting from aortic segments isolated from CTL and ecNit2$^{-/-}$ mice with quantification, normalized to CTL -VEGF (**D**). **P* < 0.05, Mann–Whitney test (-VEGF versus +VEGF). (**E**) Validation of NIT2 deletion by Western blot in aortic endothelial cells as well as other tissue of a global, constitutive knockout mouse of Nit2 (Nit2$^{wt/wt}$ and Nit2$^{ko/ko}$, generated by CRISPR/cas9 deletion of ~500 bp. Nit2$^{wt/wt}$ and Nit2$^{ko/ko}$ are littermates in a heterozygous breeding of Nit2$^{wt/ko}$ with Nit2$^{wt/ko}$ mice.). (**F**) Retina angiogenesis in neonatal mice with quantification (**G**). **P* < 0.05 Nit2$^{wt/wt}$ as compared to Nit2$^{ko/ko}$, Mann–Whitney test. SMC aortic smooth muscle cells. Source data are available online for this figure.

 © The Author(s)

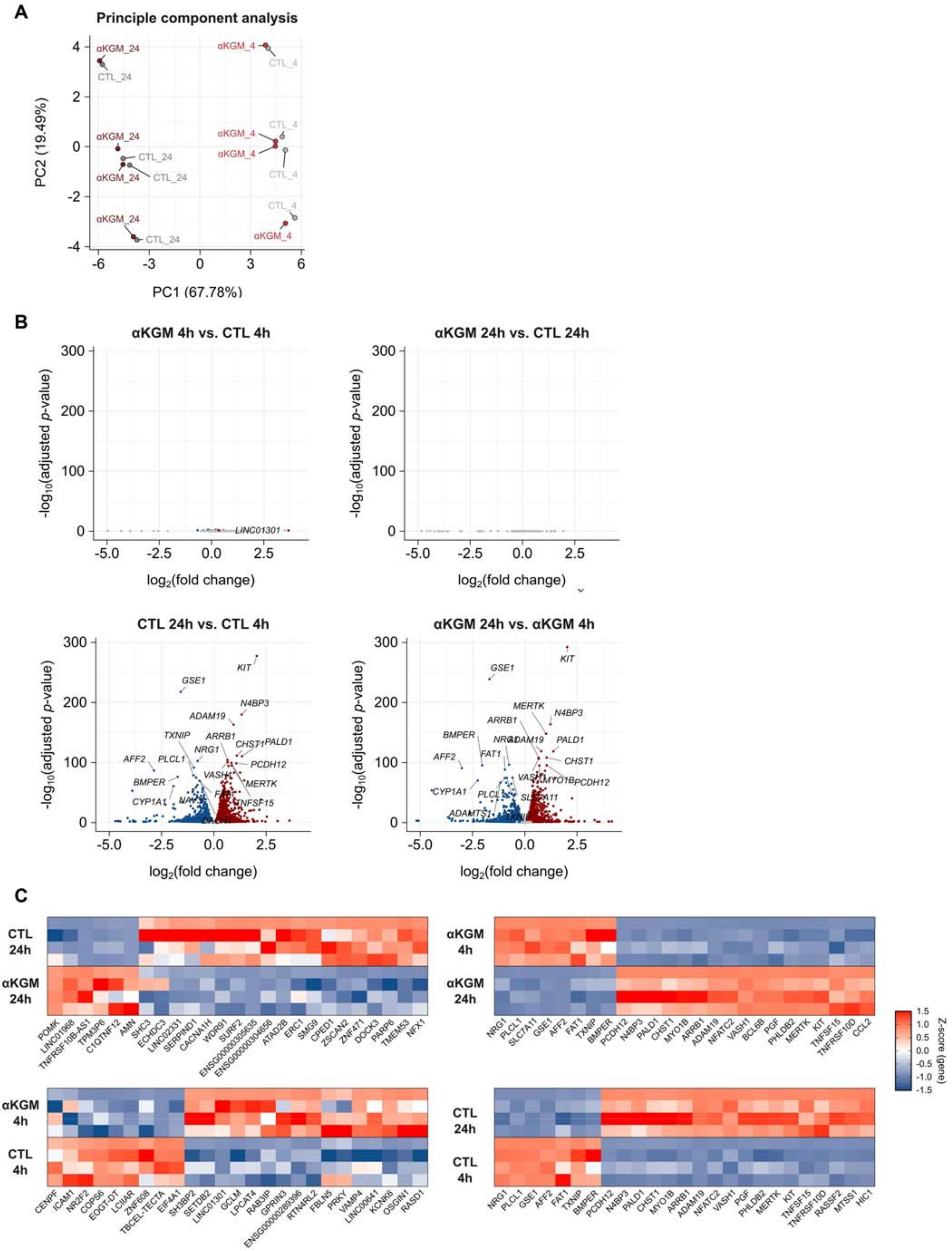
A
Principle component analysis
PC2 (19.49%)
PC1 (67.78%)
αKGM_24
CTL_24
αKGM_4
CTL_4
B
αKGM 4h vs. CTL 4h
αKGM 24h vs. CTL 24h
CTL 24h vs. CTL 4h
αKGM 24h vs. αKGM 4h
-log10(adjusted p-value)
log2(fold change)
LINC01301
KIT
GSE1
N4BP3
ADAM19
MERTK
ARRB1
TXNIP
CHST1
PALD1
NRG1
AFF2
PLCL1
PCDH12
VASH1
BMPER
CYP1A1
TNFSF15
FAT1
MYO1B
SLC7A11
ADAMTS1
C
CTL 24h
αKGM 24h
αKGM 4h
CTL 4h
POMK
LINC01068
TNFRSF10B-AS1
TPM3P6
C1QTNF12
AMN
SHC3
ECHDC3
LINC02331
SERPIND1
CACNA1H
WDR91
SURF2
ENSG00000305635
ENSG00000304656
ATAD2B
ERC1
SMG9
CPED1
ZSCAN2
ZNF471
DOCK3
PARP6
TMEM53
NFX1
NRG1
PLCL1
SLC7A11
GSE1
AFF2
FAT1
TXNIP
BMPER
PCDH12
N4BP3
PALD1
CHST1
MYO1B
ARRB1
ADAM19
NFATC2
VASH1
BCL6B
PGF
PHLDB2
MERTK
KIT
TNFSF15
TNFRSF10D
CCL2
Z-score (gene)
CENPF
ICAM1
NR2F2
COPS6
EOGT-DT
LCIIAR
ZNF608
TBCEL-TECTA
EIF4A1
SH3BP2
SETDB2
LINC01301
GCLM
LPCAT4
RAB3IP
GPRIN3
ENSG00000289396
RTN4RL2
FBLN5
PRKY
VAMP4
LINC00641
KCNK6
OSGIN1
RASD1
RASSF2
MTSS1
HIC1

Figure EV5. Effect of αKGM on gene expression in endothelial cells.

(**A**) PCA analysis, volcano plots (**B**) and heat maps (**C**) of gene expression (RNAseq) in HUVEC without (CTL) or with 2-hydroxy-5-oxo-proline (300 μM, αKGM) after 4 or 24 h of exposure to the compound. In this analysis, only time segregates the sample groups but not the treatment with αKGM suggesting that the compound is inert once it is secreted by cells.

© The Author(s)

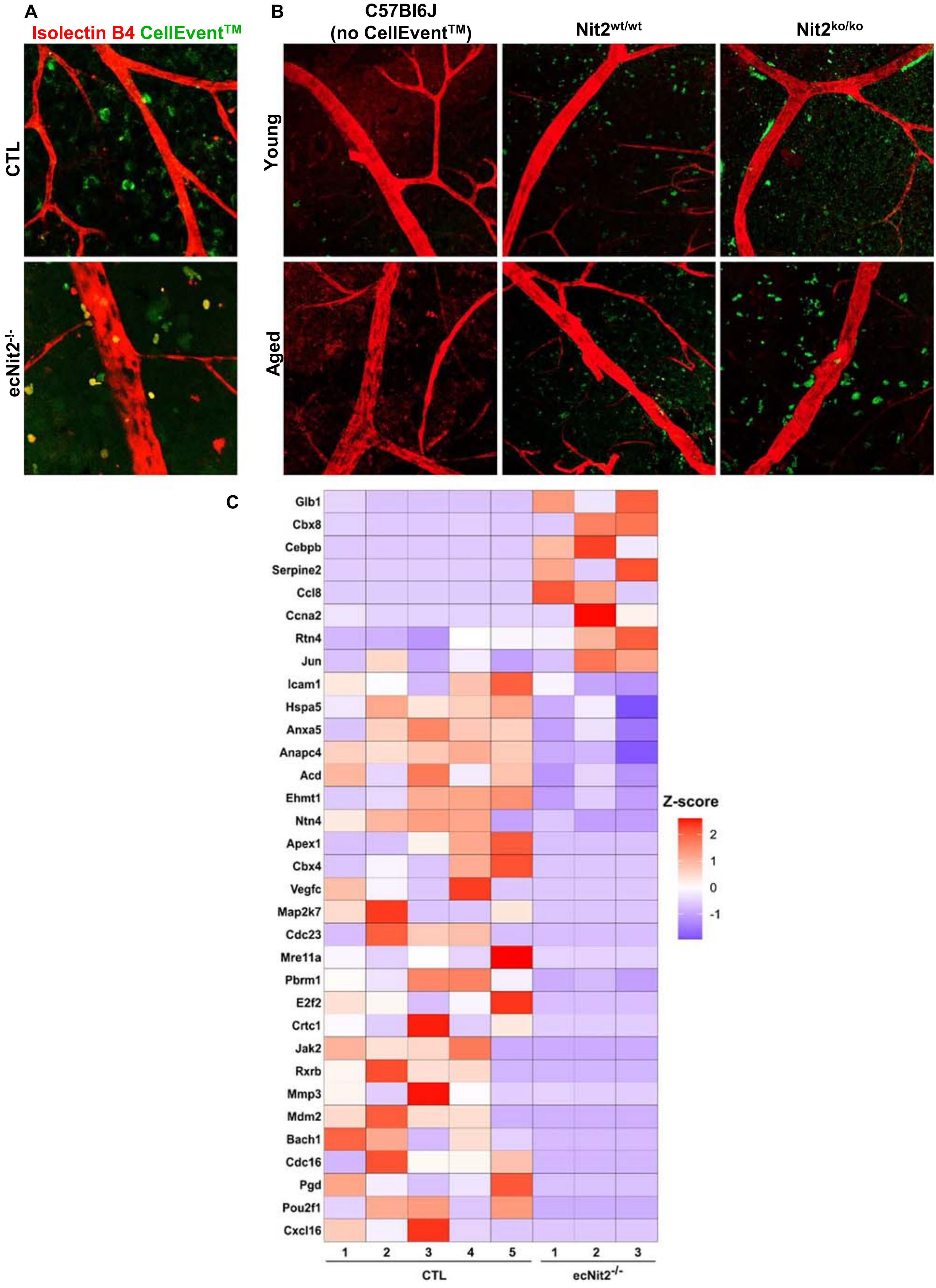
A
Isolectin B4 CellEvent™
CTL
ecNit2-/-
B
C57Bl6J (no CellEvent™)
Nit2wt/wt
Nit2ko/ko
Young
Aged
C
Glb1
Cbx8
Cebpb
Serpine2
Ccl8
Ccna2
Rtn4
Jun
Icam1
Hspa5
Anxa5
Anapc4
Acd
Ehmt1
Ntn4
Apex1
Cbx4
Vegfc
Map2k7
Cdc23
Mre11a
Pbrm1
E2f2
Crtc1
Jak2
Rxrb
Mmp3
Mdm2
Bach1
Cdc16
Pgd
Pou2f1
Cxcl16
1 2 3 4 5 1 2 3
CTL
ecNit2-/-
Z-score
2
1
0
-1

Figure EV6. Cellular senescence in knockout mice of NIT2.

CellEvent^{TM} and isolectin B4 staining in retinae of CTL and ecNit2$^{-/-}$ mice (**A**) or Nit2$^{wt/wt}$ and Nit2$^{ko/ko}$ (**B**) young (3 months) and aged (9 months). (**C**) Differentially and significantly expressed genes (RNAseq with MACE) annotated to senescence in endothelial cells enriched from carotid arteries of CTL and ecNit2$^{-/-}$ mice. Source data are available online for this figure.

 © The Author(s)

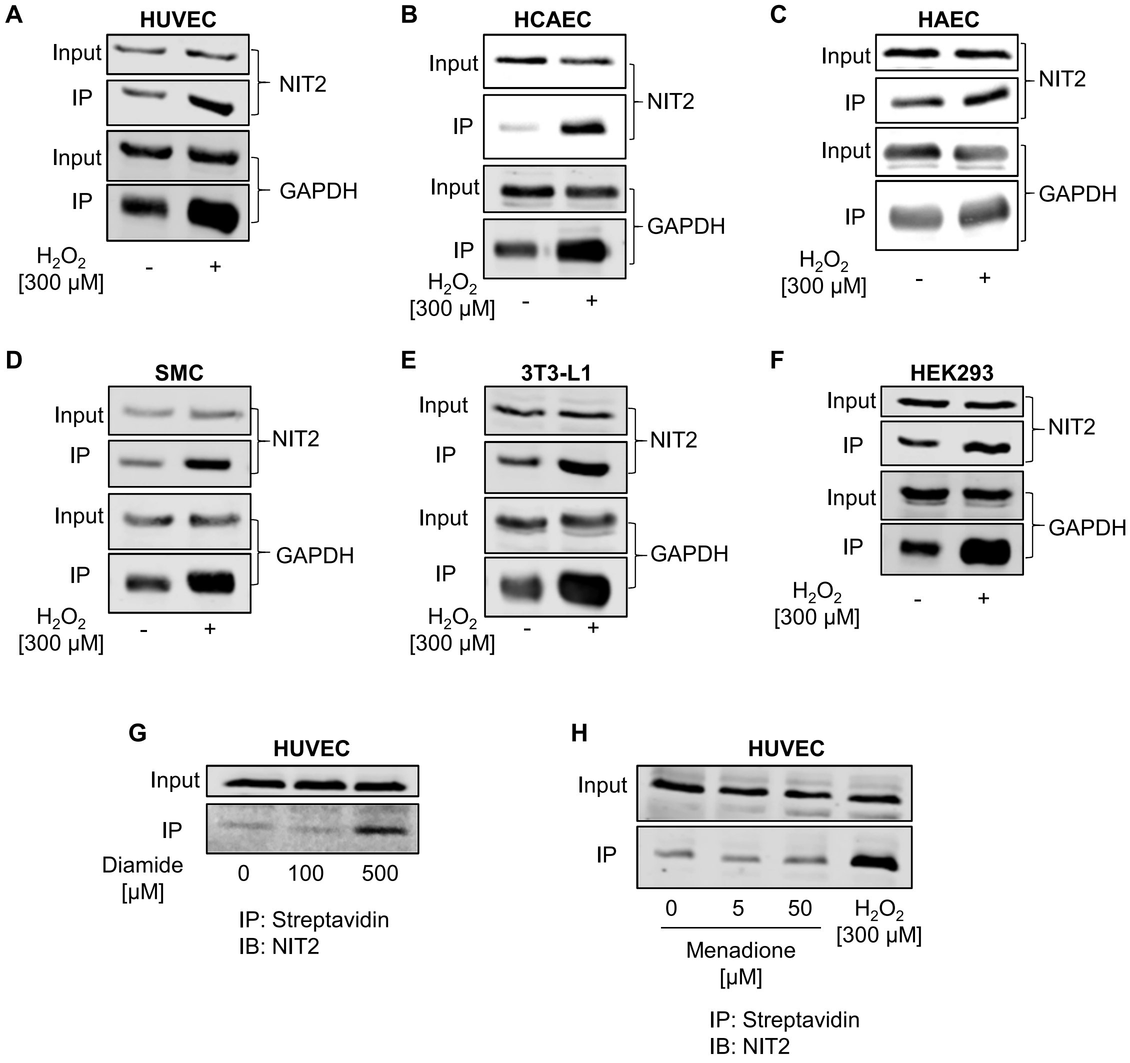

Figure EV7. Cysteine oxidation in NIT2.

Biotinylated iodoacetamide (BIAM) switch assay in different cell types exposed or not to 300 µM H_2O_2, 15 min. (**A**) HUVEC, (**B**) HCAEC, (**C**) HAEC, (**D**) SMC, (**E**) mouse fibroblasts 3T3-L1, (**F**) HEK 293. BIAM switch assay in HUVEC exposed to diamide (**G**) or menadione (**H**). HUVEC human umbilical vein endothelial cells, HCAEC human coronary artery endothelial cells, HAEC human aortic endothelial cells, SMC smooth muscle cells, IP immunoprecipitation, IB immunoblotting, GAPDH Glyceraldehyde-3-Phosphate Dehydrogenase. Source data are available online for this figure.

© The Author(s)

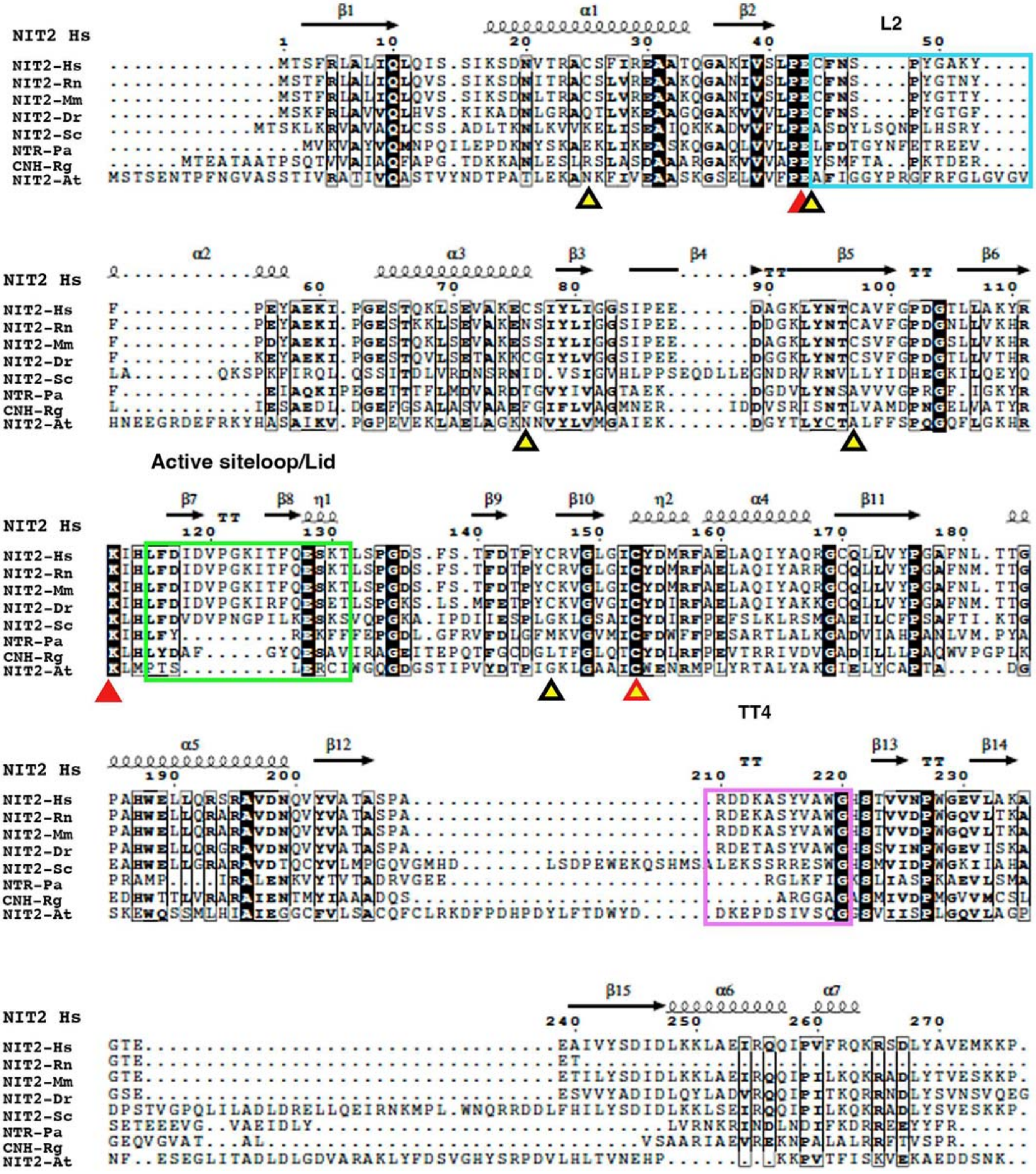

Figure EV8. Animal NIT2 contains more cysteine residues than that of other organisms or plants.

Multiple sequence alignment and structural features of human NIT2 predicted by AlphaFold2. Abbreviations: Hs, *Homo sapiens* (Q9NQR4); Rn, *Rattus norvegicus* (Q497B0); Mm, *Mouse musculus* (Q9JHW2); Dr, *Danio rerio* (Q4VBV9); Sc, *Saccharomyces cerevisiae* (P47016); Pa, *Pyrococcus abyssi* (Q9UYV8); Rg, *Rhodococcus qingshengii* (A0AA46MND0); At, *Arabidopsis thaliana* (P32962). The human cysteine residues are indicated with yellow triangles, the catalytic residues as red triangles, and the loops involved in the formation of the substrate channel and active site are colored in blue, green, and pink squares.

 © The Author(s)

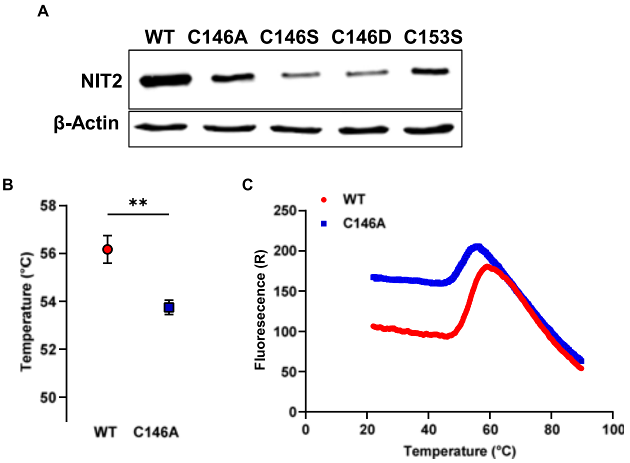

Figure EV9. Expression and stability of NIT2 C146 mutants.

(**A**) Western blot analysis (using an anti-His-tag antibody) for the His-tagged mutants of NIT2 C146 expressed in HEK 293 cells. (**B**) Aggregation points of NIT2 and NIT2 C146A as determined by thermal shift assay. $n = 3$; **$P < 0.01$, Welchs' correction. (**C**) Melting curve of purified NIT2 and NIT2 C146A protein. Source data are available online for this figure.

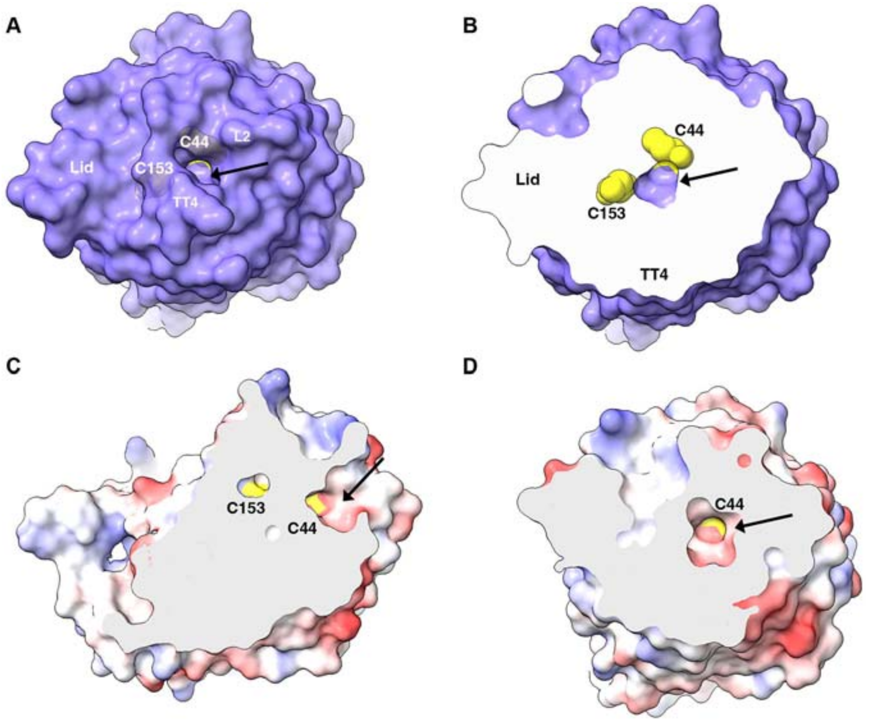

Figure EV10. Cysteine 44 forms the substrate channel and is prone to oxidation.

The AlfaFold2 structure prediction of NIT2 is depicted as the solvent-exposed surface in purple, and the substrate channel entry is shown from the top view (**A**) and the cut view to the bottom (**B**). The surface charge representation (red negative, white hydrophobic and blue positive) is shown on the side cut view (**C**) and top cut (**D**). Cys153 and Cys44 depicted as yellow spheres. The black arrow indicates the channel entry.

 © The Author(s)
